# Network Pharmacology-Guided Identification of *Candida albicans* Secondary Metabolites as Modulators of HIV Latency via Oncogenic Signaling Pathways

**DOI:** 10.3390/ijms27073125

**Published:** 2026-03-30

**Authors:** Ernest Oduro-Kwateng, Ugochukwu J. Anyaneji, Asiphe Fanele, Ntokozo Ntanzi, Mahmoud E. Soliman, Nompumelelo P. Mkhwanazi

**Affiliations:** 1 HIV Pathogenesis Programme, School of Laboratory Medicine and Medical Sciences, College of Health Science, University of KwaZulu-Natal, Durban 4013, South Africa; 223149553@stu.ukzn.ac.za (E.O.-K.); 217080488@stu.ukzn.ac.za (U.J.A.); 219087073@stu.ukzn.ac.za (A.F.); 221021741@stu.ukzn.ac.za (N.N.); 2 Molecular Bio-Computation and Drug Design Research Group, School of Health Sciences, College of Health Science, University of KwaZulu-Natal, Durban 4000, South Africa; soliman@ukzn.ac.za

**Keywords:** HIV latency, *Candida albicans*, secondary metabolites, network pharmacology, molecular modelling, natural products

## Abstract

HIV latency, driven by a complex interplay of host factors, remains a key barrier to viral clearance. Current latency-reversing agents (LRAs) demonstrate limited efficacy and specificity, and none have been approved for clinical use. Although natural products have shown promise as LRAs, the therapeutic potential of fungal metabolites remains underexplored. *Candida albicans*, a prevalent human commensal and opportunistic pathogen, produces diverse secondary metabolites that can influence host pathways, affecting latency dynamics. This study aimed to investigate the latency-modulating potential of secondary metabolites of *C. albicans* using an integrative network pharmacology and computational pipeline. *C. albicans* secondary metabolites were retrieved from the literature, screened for drug-likeness, and mapped to human targets and biological pathways annotated in HIV latency. Key metabolites, hub genes, and pathways were systematically characterized through network and computational analyses. Six drug-like candidates, identified from 185 absorption, distribution, metabolism, excretion, and toxicity (ADMET)-screened metabolites, collectively mapped to 369 human genes with a 6.5% overlap in HIV latency (176 shared and 20 hub genes). These overlapping genes were significantly enriched for signal transduction, membrane localization, and adaptive responses to chemical stimuli. Kyoto encyclopedia of genes and genomes (KEGG) enrichment revealed oncogenic diseases (non-small cell lung, pancreatic, and prostate cancers) and latency-associated cascades, including PD-L1/PD-1, HIF-1, Ras, PI3K-Akt, calcium, and cAMP signaling. Six hub targets (MAPK1, PIK3CA, MAPK3, EGFR, MTOR, and AKT1) were consistently annotated within the top 30 KEGG pathways and displayed strong binding affinities for MET 15 and MET 119. Molecular dynamics (MD) simulations confirmed favorable binding free energies (BFEs) and stable conformational dynamics for the top-ranked metabolite MET 15. *C. albicans* secondary metabolites preferentially target oncogenic signaling networks central to HIV latency maintenance, notably PI3K/AKT/MTOR and MAPK/ERK, which regulate cell survival, metabolic homeostasis, and viral transcriptional repression. MET 15 is a top-ranked candidate metabolite for HIV latency-reversing therapeutics and warrants experimental validation in established latency models.

## 1. Introduction

Eradication of human immunodeficiency virus type 1 (HIV-1) remains an unsolved global health challenge, primarily due to the persistence of latent viral reservoirs that evade clearance by combination antiretroviral therapy (cART). These reservoirs, predominantly housed within resting CD4^+^ T cells, lymphoid tissues, gut-associated lymphoid tissue (GALT), and anatomical sanctuaries such as the central nervous system (CNS), persist in a transcriptionally silent state, rendering them refractory to immune surveillance and pharmacological intervention [1,2]. Notably, the CNS poses a unique obstacle due to limited drug penetrance, immune privilege, and the presence of specialized reservoir cell types, necessitating novel therapeutic strategies capable of targeting latency across compartmentalized reservoirs [3]. Current latency-modulating strategies are primarily centred on the “shock and kill” and “block and lock” paradigms. The former seeks to reactivate latent proviruses using latency-reversing agents (LRAs), such as histone deacetylase inhibitors (HDACis), protein kinase C (PKC) agonists, toll-like receptor (TLR) agonists, and bromodomain and extraterminal domain (BET) inhibitors, followed by immune-mediated or cytopathic clearance [4]. In contrast, “block and lock” approaches aim to suppress viral transcription, thereby locking HIV into deep latency [5]. Although clinical trials of LRAs such as romidepsin, bryostatin-1, and JQ1 have demonstrated mechanistic activity, their clinical success has been hindered by insufficient latency reversal, immunotoxicity, and heterogeneity of latent reservoirs [6,7]. This underscores the ongoing need for novel and safer LRAs, particularly those with microbiome-derived origin, which may offer unique therapeutic advantages in HIV-candidiasis co-infection.

Opportunistic infections are a hallmark of HIV disease progression, with *Candida albicans* emerging as one of the most prevalent fungal pathogens in people living with HIV (PLWH) [8]. *C. albicans* frequently colonizes mucosal surfaces and not only breaches epithelial barriers but also influences immune homeostasis by modulating host signaling pathways, such as NF-κB, IL-17, and MAPK signaling [9,10]. These immunomodulatory effects may influence the activation state of CD4^+^ T cells and cytokine environment, contributing to the establishment or reversal of HIV latency. Among the secreted metabolites, candidalysin and farnesol have been characterized for their immunological activity. Candidalysin is a pore-forming peptide toxin secreted during hyphal growth that disrupts epithelial membranes and activates innate immune signaling pathways, including the MAPK and NF-κB pathways. It also induces ligand shedding, primarily via epiregulin and epigen, which activates EGFR signaling and amplifies innate immune responses. Additionally, candidalysin activates the NLRP3 inflammasome in immune cells, such as macrophages and dendritic cells, leading to IL-1β secretion and promoting inflammatory cascades [11]. Farnesol, a sesquiterpene alcohol, functions as a quorum-sensing molecule that regulates fungal morphogenesis and virulence and modulates host immune signaling pathways. Farnesol activates innate immune cells by inducing pro-inflammatory signaling through the MAPK and NF-κB pathways, resulting in cytokine release and oxidative burst responses. At the same time, it has been reported to impair dendritic cell maturation by downregulating the granulocyte-macrophage colony-stimulating factor (GM-CSF) receptor, thereby reducing T-cell activation. Farnesol may also alter immune polarization by promoting inflammatory responses and suppressing Th1-mediated immunity [12]. Despite these known host–pathogen interactions, the potential of *C. albicans* secondary metabolites to directly target human proteins involved in HIV latency regulation has not been systematically explored, representing an untapped chemical space for latency-modulating therapeutic discovery. Importantly, fungal metabolites from other species have shown potential to modulate HIV latency. For instance, gliotoxin from *Aspergillus fumigatus* disrupts the 7SK small nuclear ribonucleoprotein (snRNP) complex, thereby releasing positive transcription elongation factor b (P-TEFb) and promoting HIV reactivation [13]. Chaetocin from *Chaetomium minutum* acts as a Suv39H1 histone methyltransferase inhibitor (HMTi) and has been shown to reverse HIV latency by demethylating repressive histone marks, thereby enhancing transcription at the HIV long terminal repeat (LTR) region [14]. Harziachalasin D, derived from the endophytic fungus *Trichoderma harzianum* MLJ-4, reverses HIV latency by activating the NF-κB pathway. This activation leads to the phosphorylation and degradation of inhibitor of nuclear factor kappa B alpha (IκBα), facilitating NF-κB nuclear translocation and HIV transcriptional activation [15]. These findings underscore fungal species as a largely unexplored source of bioactive small molecules with potential as LRAs.

Given the immunological relevance of *C. albicans* in HIV co-infection and the precedent set by other fungal metabolites, we hypothesized that certain *C. albicans*-derived secondary metabolites could modulate key host pathways involved in HIV-1 latency. To investigate this, we employed an integrative network pharmacology and *in silico* approach. Network pharmacology provides a holistic framework for characterizing the multi-target effects of bioactive compounds by integrating pharmacokinetic profiling, target prediction, protein–protein interaction (PPI) analysis, and pathway enrichment within the context of systems biology [16,17]. Although emerging in HIV cure research, this approach has begun to elucidate host dependency pathways and potential therapeutic targets involved in viral persistence, as well as the repurposing of drugs for HIV cure [18,19,20]. In this study, *C. albicans* metabolites were curated from the literature and screened for drug-likeness, oral bioavailability (OB), blood–brain barrier (BBB) permeability, and toxicity. Potential human protein targets were predicted and cross-referenced with the HIV latency-associated proteins. Subsequently, PPI network construction, topological analysis, and functional enrichment analyses were performed to reveal key biological processes and signaling pathways. Finally, molecular docking, molecular dynamics (MD) simulations, and binding free energy (BFE) calculations were performed to evaluate metabolite–target (MET–TAR) binding affinities.

**Figure 1:**
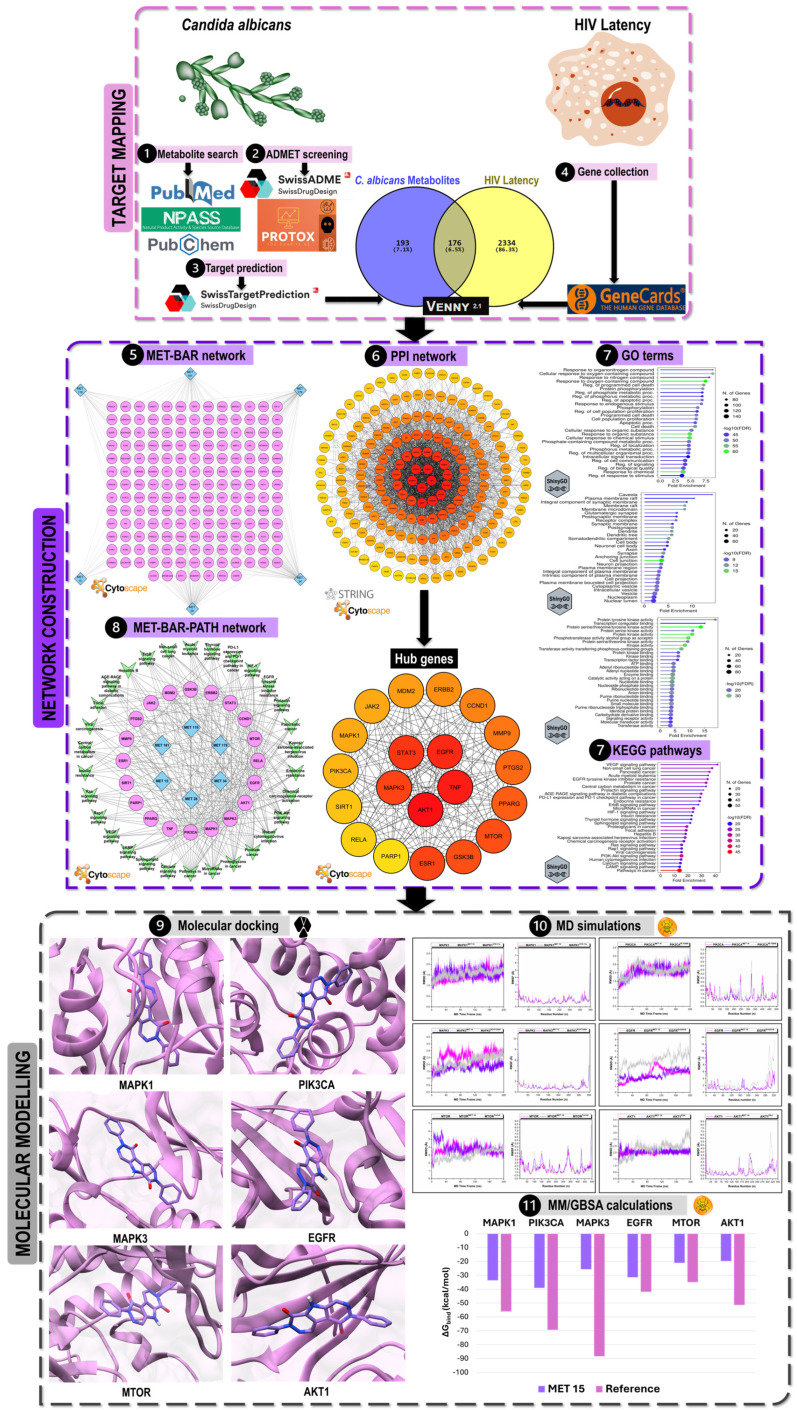
Graphical framework of the methodology used in investigating the HIV latency-modulation potential of *Candida albicans* secondary metabolites.

Figure 1 shows the methodological framework used in this study. Our findings highlight that the enrichment of signaling cascades, while predominantly oncogenic, are critical host dependency factors for HIV-1 transcriptional regulation. To the best of our knowledge, this is the first comprehensive investigation on the role of *C. albicans* secondary metabolites as modulators of HIV-1 latency. These findings suggest a previously underexplored microbial metabolite space with potential relevance to HIV latency modulation.

## 2. Results

### 2.1. Drug-likeness and Absorption, Distribution, Metabolism, Excretion, and Toxicity (ADMET) Screening for Therapeutic Metabolites

Six candidate metabolites were identified as described in Section 4. All six metabolites complied with Lipinski’s Rule of Five (Ro5) with no observed violations, supporting their drug-like properties and OB. The results are presented in Table 1. Molecular weights (MWs) ranged from 222.37 g/mol (MET 181) to 411.43 g/mol (MET 119), and all compounds had favorable bioavailability score (BS) of 0.55 and 0.85 (MET 176). The topological polar surface area (TPSA) values ranged from 20.23 to 138.51 Å^2^. Gastrointestinal (GI) absorption was predicted to be high for five compounds, with MET 119 being the only exception (low absorption). Moreover, all compounds are non-substrates of P-glycoprotein (Pgp). Three metabolites (MET 28, MET 176, and MET 181) were predicted to cross the BBB. Cytochrome P450 3A4 (CYP3A4) inhibition was predicted for MET 28 and MET 119. Solubility predictions showed moderate solubility for most compounds, with MET 34 classified as very soluble and MET 119 as soluble. None of the compounds contain pan-assay interference compounds (PAINS) alerts, and the synthetic accessibility (SA) scores ranged from 2.51 to 3.26. The median lethal dose (LD_50_) values ranged from extremely low in MET 34 (41 mg/kg, Class II) and MET 176 (48 mg/kg, Class II) to exceptionally high in MET 119 (25,000 mg/kg, Class VI). LD_50_ values were moderately high for MET 15 (1500 mg/kg, Class IV) and MET 181 (5000 mg/kg, Class V). All the metabolites were inactive with respect to nephrotoxicity, cardiotoxicity, immunotoxicity, and cytotoxicity. However, only one metabolite flagged specific concerns based on high-confidence predictions (confidence score ≥ 0.70). MET 15 was predicted to be neurotoxic (active: 0.88). Other endpoints such as hepatotoxicity, mutagenicity, and respiratory toxicity were either inactive or predicted with low confidence (confidence score < 0.70). The predicted toxicity profiles of the metabolites are illustrated in the radar chart in Supplementary Figure S1.

### 2.2. Target Prediction and HIV Latency Relevance Mapping of Candida albicans Secondary Metabolites

**Figure 2:**
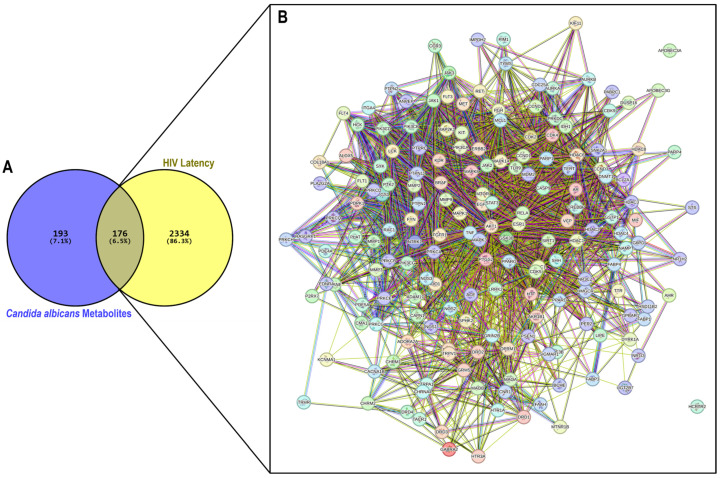
Overlapping target analysis and PPI network of *Candida albicans* secondary metabolites in the context of HIV latency. (**A**) Venn diagram illustrates the intersection between the predicted human targets of *C. albicans* metabolites (369 targets) and HIV latency-associated human genes (2510 genes), identifying 176 overlapping targets (6.5%) as potential modulators of HIV latency. (**B**) PPI network of 176 shared targets constructed using STRING database v12.0 (confidence score ≥ 0.400). Nodes represent proteins, and edges indicate known or predicted functional associations, including co-expression (black), gene neighborhood (green), gene fusions (red), gene co-occurrence (dark blue), text mining (yellow), protein homology (light blue) and experimental (purple) or database evidence (cyan).

A total of 369 non-redundant targets were identified for the six selected *Candida albicans* metabolites, spanning diverse functional classes, including enzymes, kinases, lyases, and proteases (Supplementary Figure S2). The predicted targets were 39, 127, 115, 49, 124, and 97 for MET 15, MET 28, MET 34, MET 119, MET 176, and MET 181, respectively. To assess their relevance in HIV latency, a curated list of 2510 human genes associated with HIV latency was obtained from the GeneCards database using the keyword “HIV latency.” A cross-comparison of the predicted targets with the latency-associated gene set using Venny 2.1.0 revealed 176 overlapping genes (Figure 2A). The PPI network comprised 176 nodes and 2464 edges, exhibiting a significantly enriched interaction pattern (PPI enrichment *p* < 1.0 × 10^−16^), far above what would be expected for a random set of proteins (expected edges = 1009). The average node degree was 28 and the average local clustering coefficient was 0.54 (Figure 2B).

### 2.3. Construction and Topological Analysis of MET–TAR Network

**Figure 3:**
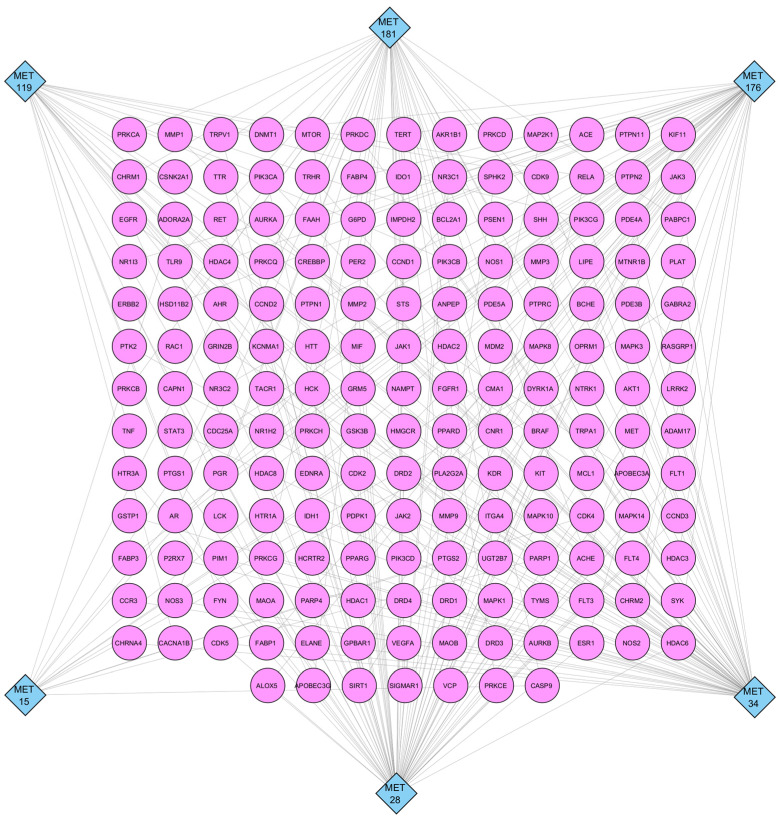
MET–TAR interaction network of *Candida albicans* metabolites and predicted human protein targets. The network comprises six key metabolites (blue diamond nodes: MET 15, MET 28, MET 34, MET 119, MET 176, and MET 181) connected to their respective protein targets (pink circular nodes). The network consists of 182 nodes and 263 edges (gray), highlighting the polypharmacological potential of *C. albicans* metabolites in modulating diverse human targets relevant to HIV latency.

The MET–TAR network comprised 182 nodes (six metabolites and 176 target proteins) and 263 edges, representing the predicted interactions between each metabolite and its associated target(s) (Figure 3). Topological analysis was performed using the NetworkAnalyzer plugin v4.5.0 in Cytoscape v3.10.3 to assess the degree centrality (DC), betweenness centrality (BC), and closeness centrality (CC) of individual nodes, which reflects the number of direct connections of a node within the network, the relative extent to which a node is positioned on the shortest paths between other nodes, and the relative proximity of a node to all other nodes in the network, respectively. MET 28 recorded the highest DC of 69, followed by MET 34, MET 176, MET 181, MET 119, and MET 15 with DC of 65, 49, 42, 24, and 14, respectively. Similarly, MET 28 showed the highest BC (0.454) and CC (0.452), followed by MET 34 (BC = 0.427, CC = 0.444), MET 176 (BC = 0.305, CC = 0.411), MET 181 (BC = 0.229, CC = 0.399), MET 119 (BC = 0.093, CC = 0.369), and MET 15 (BC = 0.040, CC = 0.355) (Table 2). Among the protein targets, PTGS1 and PTGS2 both had a DC of 5, followed by AR with a DC of 4. Other highly connected proteins included GSK3B, CASP9, HDAC6, PIK3CA, TLR9, and HDAC4 with a DC of 3, as well as CDK2, PARP1, CDK4, MIF, EGFR, AKT1, HDAC8, ESR1, CDK5, SIRT1, RELA, JAK1, JAK2, PIK3CD, and MDM2 with a DC of two (Supplementary Table S2).

### 2.4. PPI Network Analysis and Hub Target Identification

**Figure 4:**
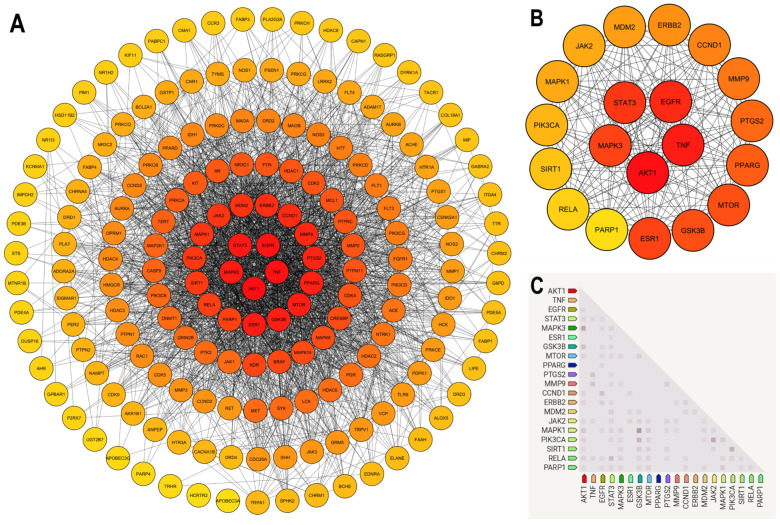
PPI network and hub gene analysis of *Candida albicans* metabolite targets relevant to HIV latency. (**A**) PPI network of 176 overlapping targets between *C. albicans* metabolites and HIV latency-related genes, visualized using Cytoscape v3.10.3. Nodes represent protein targets and edges indicate the predicted functional associations. Node colors range from yellow (low) to red (high), scaled by DC values calculated using the CytoHubba plugin v0.1, highlighting the topological significance of each node. (**B**) Subnetwork of the top 20 hub genes identified by DC ranking, including AKT1, TNF, EGFR, STAT3, MAPK3, etc., suggesting central regulatory roles in HIV latency modulation. (**C**) Co-expression heatmap of the top 20 hub genes illustrating pairwise gene correlations using ProteomeHD on STRING database v12.0. Darker tiles indicate stronger co-expression relationships, supporting functional interactions among these key latency-related targets.

The PPI network was imported into Cytoscape v3.10.3 for advanced visualization and topological analyses. The network had a diameter of 4, density of 0.164, characteristic path length of 1.988, and centralization value of 0.542. Notably, 174 nodes were part of a single connected component, suggesting robust molecular interplay among the targets. To prioritize the key regulatory targets within the network, CytoHubba v0.1 was used to calculate the DC score for each node (Figure 4A). The nodes were visualized using a red–yellow color gradient corresponding to a decreasing DC score and highlighted in a focused subnetwork (Figure 4B). The top 20 hub genes identified were AKT1, TNF, EGFR, STAT3, MAPK3, ESR1, GSK3B, MTOR, PPARG, PTGS2, MMP9, CCND1, ERBB2, MDM2, JAK2, MAPK1, PIK3CA, SIRT1, RELA, and PARP1. Furthermore, the co-expression relationships among these genes were analyzed using ProteomeHD on STRING database v12.0, with edge coloration in the network indicating the confidence level of gene–gene associations. Darker edges represent higher confidence scores, suggesting robust co-regulation or functional interdependence among several hub genes involved in HIV latency (Figure 4C). A detailed summary of the hub genes, including their Ensembl identifiers, target families, DC scores, GeneCards inferred functionality scores (GIFTS), and HIV latency relevance scores, is provided in Table 3.

### 2.5. Functional Annotation and Pathway Enrichment Analysis of Overlapping Targets

**Figure 5:**
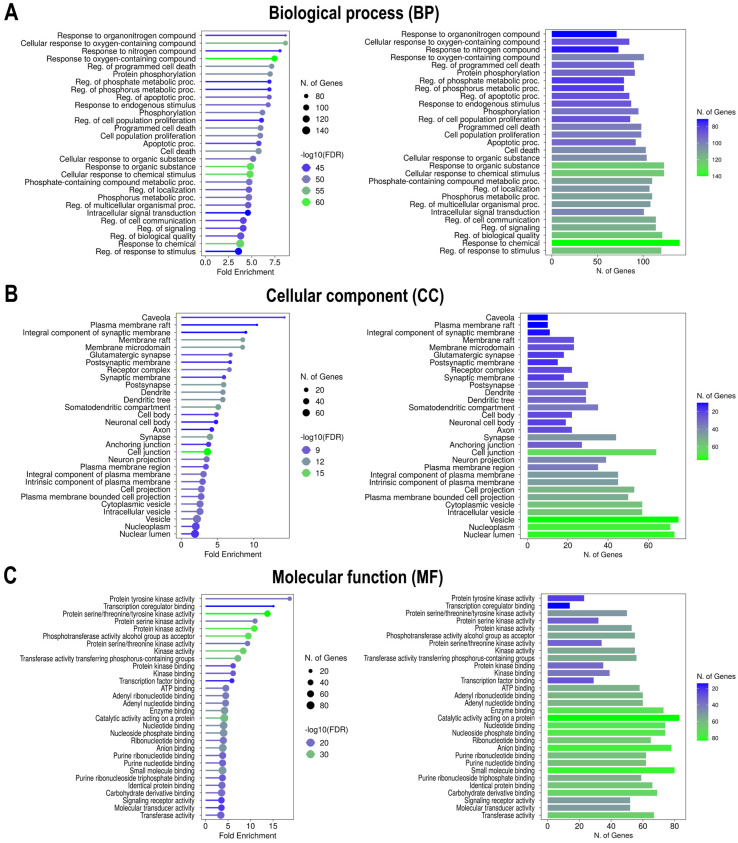
GO enrichment analysis of overlapping targets between *Candida albicans* metabolites and HIV latency-associated genes. Top 30 significantly enriched (**A**) BP terms, (**B**) CC terms, and (**C**) MF terms. The bubble size and color reflect the number of genes involved and significance level (−log_10_[FDR]), respectively, with blue indicating lower significance and green indicating higher significance, while the x-axis represents the FE values (**left** panel). Horizontal bar plots (**right** panel) indicate the number of genes annotated per term to provide additional visualization of enrichment significance.

**Figure 6:**
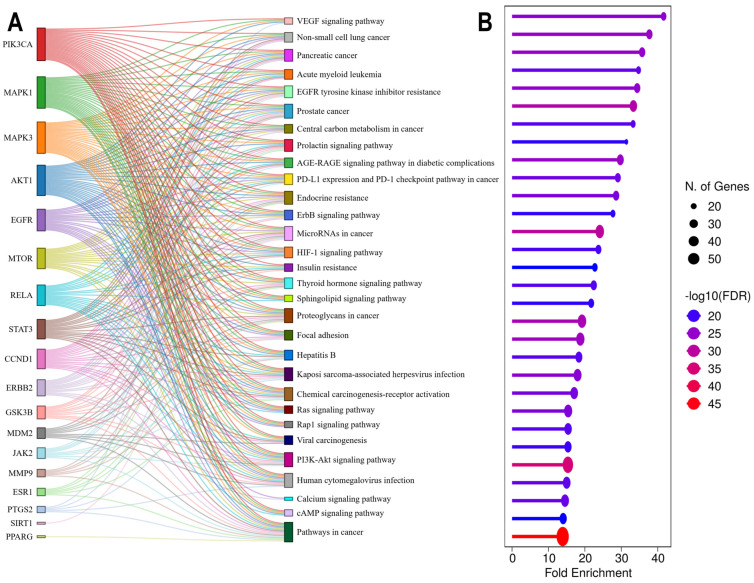
KEGG pathway enrichment analysis of overlapping targets of *Candida albicans* metabolites and HIV latency-associated genes. (**A**) Sankey diagram showing interactions between hub genes and enriched KEGG pathways. Colored flows indicate shared genes contributing to multiple pathways, highlighting the multifunctionality and potential regulatory roles of hub genes such as PIK3CA, MAPK3, MAPK1, AKT1, EGFR, and MTOR in HIV-associated mechanisms. (**B**) Bubble plot of the top 30 significantly enriched KEGG pathways based on FE and −log_10_(FDR) values. The dot size represents the number of enriched genes per pathway, whereas the dot color corresponds to the significance level, with blue indicating lower significance and red indicating higher significance. The significantly enriched pathways include VEGF signaling pathway, pathways in cancer, prostate cancer, microRNAs in cancer, and PI3K-Akt signaling pathway.

The top 30 enriched gene ontology (GO) terms in the biological process (BP), cellular component (CC), and molecular function (MF) categories, as well as the Kyoto encyclopedia of genes and genomes (KEGG) pathways, are illustrated in Figure 5A–C and Figure 6, respectively.

#### 2.5.1. GO Term Enrichment Analysis

A total of 1000 GO BP terms were identified. Among these, response to organonitrogen compound (GO:0010243), cellular response to oxygen-containing compound (GO:1901701), and response to nitrogen compound (GO:1901698) emerged as the top three enriched processes, with false discovery rate (FDR) values as low as 1.72 × 10^−45^, 1.26 × 10^−55^, and 7.81 × 10^−45^ respectively, and fold enrichment (FE) scores > 8.0. Notably, these terms also had high gene counts (71–85 genes). Although regulation of biological quality (GO:0065008) and response to chemical (GO:0042221) had slightly lower FE (<4.0), they were associated with substantially larger gene sets (121 and 140 genes, respectively) (Figure 5A and Supplementary Table S3). CC category yielded 317 enriched terms. The top-ranking terms included caveola (GO:0005901), plasma membrane raft (GO:0044853), and integral component of synaptic membrane (GO:0099699), with FE scores of 14.21, 10.43, and 8.89, respectively. However, these terms included relatively small gene sets (10–11 genes). In contrast, broader components, such as vesicle (GO:0031982), nuclear lumen (GO:0031981), and nucleoplasm (GO:0005654), overlapped with high gene sets of 75, 73, and 71, albeit at lower FE scores of 2.17, 1.90, and 2.00, respectively (Figure 5B and Supplementary Table S4). Among the 546 MF terms, the most significantly enriched were various kinase-related activities, including protein tyrosine kinase activity (GO:0004713), transcription coregulator binding (GO:0001221), protein serine/threonine/tyrosine kinase activity (GO:0004712), and protein serine kinase activity (GO:0106310), with FE scores of 18.70, 15.08, 13.75, and 11.00, respectively. These high-FE terms were complemented by broad-spectrum functions, such as catalytic activity acting on a protein (GO:0140096), small molecule binding (GO:0036094), anion binding (GO:0043168), and nucleotide binding (GO:0000166), with lower FE scores of 4.16, 3.77, 3.83, and 4.02, but high gene counts of 83, 80, 78, and 74, respectively (Figure 5C and Supplementary Table S5).

#### 2.5.2. KEGG Pathway Enrichment Analysis

**Figure 7:**
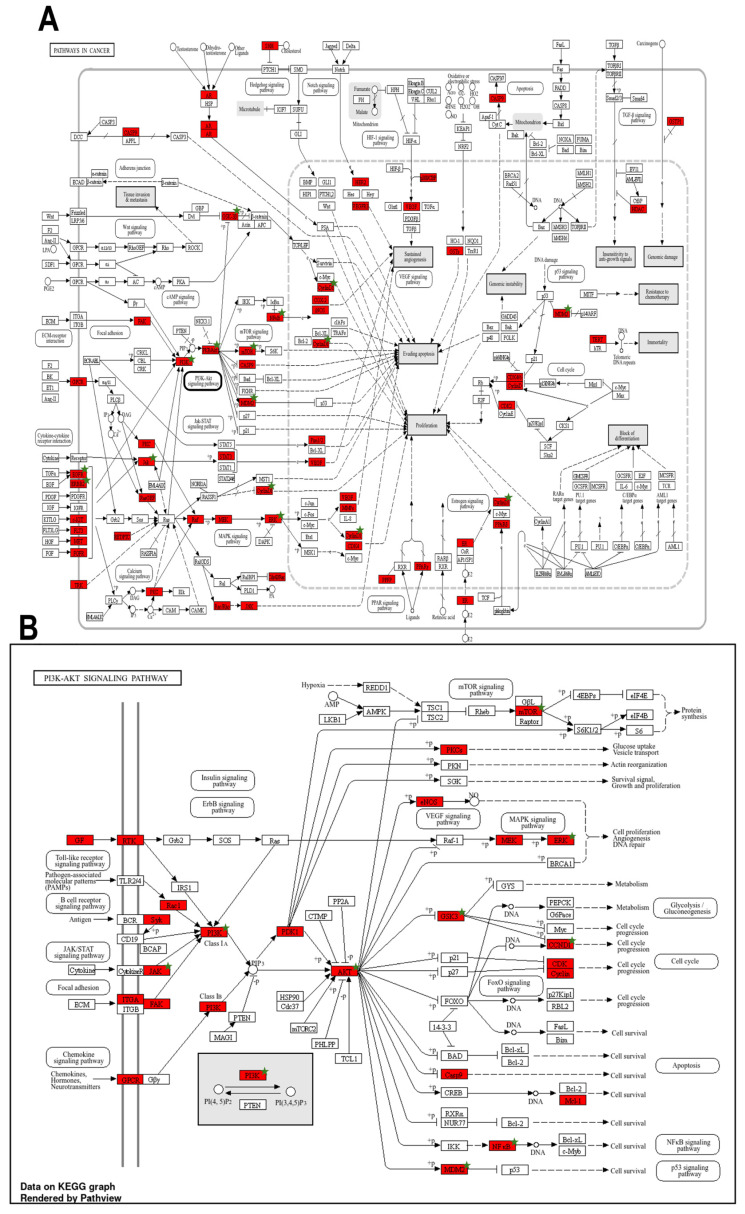
Integrated KEGG map summarizing the convergence of (**A**) pathways in cancer (hsa05200) and (**B**) PI3K-Akt signaling pathway (hsa04151) enriched by the targets of *Candida albicans* secondary metabolites overlapping with HIV latency-associated genes. The pathways were visualized using Pathview (v1.50.0) to illustrate the localization of the overlapping genes (red nodes). Hub targets common to both pathways are highlighted by green stars. Solid arrows represent direct signaling interactions and dashed arrows indicate indirect or inferred regulatory connections. Notably, 12 hub genes, AKT1, CCND1, EGFR, ERBB2, GSK3B, JAK2, MAPK1, MAPK3, MDM2, MTOR, PIK3CA, and RELA are expressed in both axes. This integrative map underscores the therapeutic potential of *C. albicans* secondary metabolites in influencing key oncogenic nodes related to cell survival, metabolism, proliferation, and apoptosis, in the context of HIV latency modulation.

Of the 217 KEGG pathways identified, VEGF signaling pathway (hsa04370) emerged as the most overrepresented, with an FE of 41.63, overlapping with 19 genes. Other strongly enriched pathways included cancer-related pathways, including non-small cell lung cancer (hsa05223; FE = 37.70), pancreatic cancer (hsa05212; FE = 35.72), acute myeloid leukemia (hsa05221; FE = 34.73), EGFR tyrosine kinase inhibitor resistance (hsa01521; FE = 34.36), prostate cancer (hsa05215; FE = 33.32), and central carbon metabolism in cancer (hsa05230; FE = 33.24). In contrast, broader signaling mechanisms, such as microRNAs in cancer (hsa05206; 30 genes), PI3K-Akt signaling (hsa04151; 42 genes), and pathways in cancer (hsa05200; 57 genes), demonstrated extensive gene participation but slightly lower FE scores of 24.09, 15.34, and 13.90, respectively (Figure 6B and Supplementary Table S6). A Sankey diagram was generated to visualize the interplay between prioritized genes and pathways (Figure 6A). The visualization revealed functional pleiotropy for several hub genes, including PIK3CA, MAPK3, MAPK1, AKT1, EGFR, MTOR, RELA, and STAT3, which mapped to multiple key pathways, such as pathways in cancer (hsa05200), PI3K-Akt signaling pathway (hsa04151), proteoglycans in cancer (hsa05205), microRNAs in cancer (hsa05206), and prostate cancer (hsa05215). Interestingly, TNF and PARP1, despite being high-ranking genes, did not map to any of the top 30 enriched KEGG pathways, suggesting a potentially distinct mechanism of action outside this oncogenic-rich canonical pathway enrichment. Furthermore, based on their extremely low FDR values, high FE scores, and dense number of associated overlapping and hub genes, pathways in cancer (hsa05200) and PI3K-Akt signaling pathway (hsa04151) emerged as the most significantly enriched pathways in our analysis. Notably, both pathways have established roles in the regulation of HIV latency (Figure 7). The enriched KEGG pathways from our analysis were systematically compared with those in published literature on the mechanisms underlying HIV latency. We identified ten pathways with established or emerging evidence of involvement in HIV latency (Table 4).

### 2.6. Topological Analysis of the Metabolite–Target–Pathway (MET–TAR–PATH) Network

**Figure 8:**
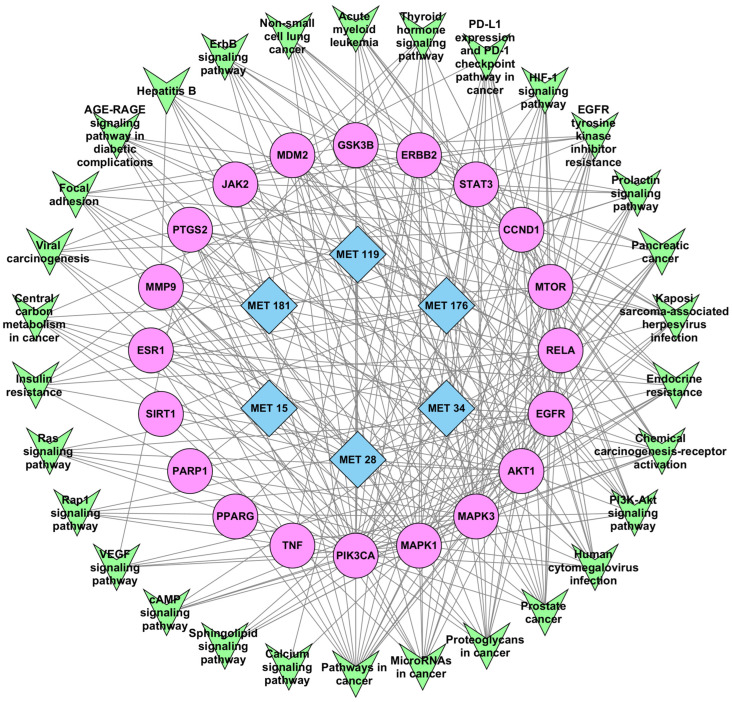
MET–TAR–PATH network integrates the six candidate *Candida albicans* secondary metabolites (blue diamonds), 20 hub targets (pink circles), and the top 30 enriched KEGG pathways (green arrows). Key hub targets PIK3CA, MAPK1, AKT1, EGFR, RELA, MTOR, CCND1, ERBB2, and GSK3B are potentially regulated by MET 28 and participate in multiple pathways, including pathways in cancer, PI3K-Akt signaling, and prostate cancer. The dense interconnectivity underscores the potential of MET 28 as a multitarget modulator with implications for HIV latency dynamics.

To investigate the integrative relationships among *Candida albicans* metabolites, their associated biological targets, and enriched signaling pathways, a MET–TAR–PATH network was constructed using Cytoscape v3.10.3. The merged network comprised 56 nodes, including six metabolites, 20 hub gene targets, and 30 KEGG pathways interconnected by 301 edges (Figure 8). Topological analysis involved DC calculations to identify several key nodes. Among the metabolites, MET 28 exhibited the most extensive interactions, mapping to 13 hub targets. MET 34, MET 176, MET 119, MET 181, and MET 15 mapped to eight, six, five, three, and three hub genes, respectively. Among the hub targets, PIK3CA exhibited the highest DC (32), followed by MAPK3 (29), MAPK1 (29), AKT1 (29), EGFR (21), RELA (20), and MTOR (19). This makes PIK3CA the “central bridge” of the tripartite network. In contrast, less connected targets included SIRT1 (3), PPARG (2), PARP1 (2), and TNF (1). Among the enriched pathways, pathways in cancer (hsa05200) mapped to 17 hub targets, human cytomegalovirus infection (hsa05163), proteoglycans in cancer (hsa05205), PI3K-Akt signaling (hsa04151), microRNAs in cancer (hsa05206), and prostate cancer (hsa05215) each mapped to 12 hub targets. The lowest hub target-expressed pathways were cAMP signaling pathway (hsa04024), Rap1 signaling pathway (hsa04015), sphingolipid signaling pathway (hsa04071), and VEGF signaling pathway (hsa04370), each with five connections. (Supplementary Table S8). Collectively, these findings underscore the potential multitarget therapeutic relevance of *C. albicans* metabolites in addressing HIV latency through the coordinated modulation of interconnected signaling networks that govern both cell cycle/oncogenic processes and viral latency.

### 2.7. Binding Affinity Calculations

**Figure 9:**
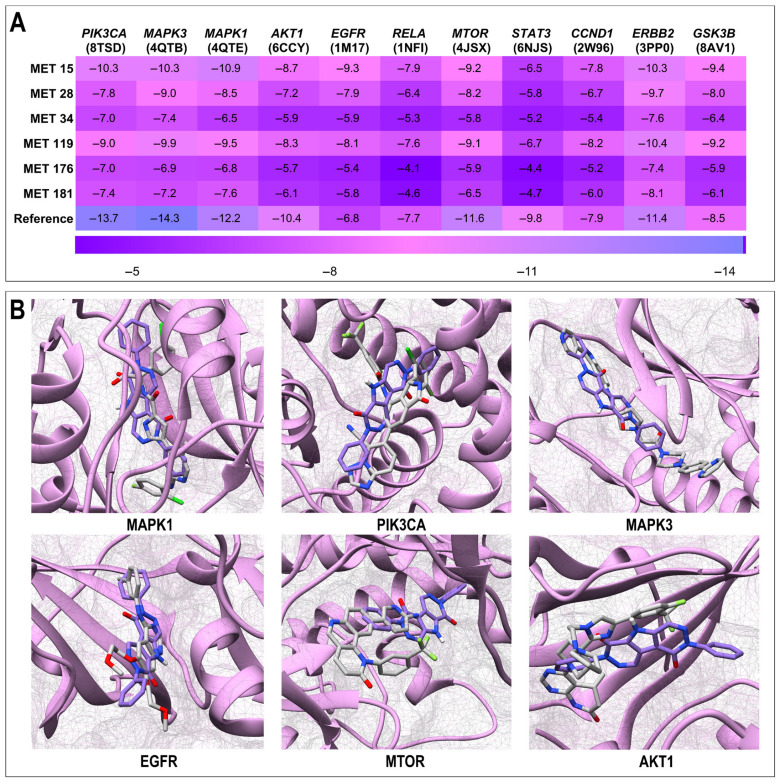
Binding affinity and docking landscape of *Candida albicans* secondary metabolites with key HIV latency-related targets. (**A**) Heatmap of the molecular docking scores (kcal/mol) between *C. albicans* metabolites and prioritized HIV latency-associated hub gene targets. The columns represent the 11 key proteins and the rows represent the six metabolites. Docking scores are color-coded from cornflower blue (weaker binding) to pink and purple (stronger binding), with more negative values indicating a higher binding affinity. (**B**) Comparative molecular docking poses of the top six protein–ligand complexes, showing MET15 bound to MAPK1, PIK3CA, MAPK3, EGFR, MTOR, and AKT1, along with their respective reference inhibitors VTX-11e, RLY2608, SCH772984, Erlotinib, Torin2, and EX4. Ligands are displayed in stick representation—medium purple for the metabolites and grey for the reference inhibitors—within the binding pockets of the target proteins, shown as mesh surface ribbons in plum. The visual comparison underscores the binding site overlap and potential mimicry of reference ligand interactions by the metabolites.

To evaluate the binding affinity of the prioritized *Candida albicans* secondary metabolites with key HIV latency-related targets, molecular docking simulations were performed for 16 hub proteins identified through integrated MET–TAR–PATH analyses. The docking scores (binding energies in kcal/mol) are summarized in Supplementary Table S7 and are visualized as a heatmap (Figure 9A). Docking scores below −5.0 kcal/mol are generally indicative of favorable interactions and stable binding conformations. Across all six metabolites (MET 15, MET 28, MET 34, MET 119, MET 176, and MET 181), the binding scores ranged from −11.6 to −4.1 kcal/mol, confirming appreciable interactions with multiple hubs. Notably, MET 15 consistently exhibited strong binding, particularly with MMP9 (−11.6 kcal/mol), MAPK1 (−10.9 kcal/mol), ERBB2 (−10.4 kcal/mol), and both MAPK3 and PIK3CA (−10.3 kcal/mol), in some cases approaching those of the reference ligands. MET 119 also demonstrated broad and favorable activity, with binding energies ≤ −9.0 kcal/mol against MMP9, ERBB2, MAPK3, JAK2, MAPK1, GSK3B, MTOR, and PIK3CA. MET 28 displayed selective high-affinity interactions, particularly with ERBB2 (−9.7 kcal/mol) and MAPK3 (−9.0 kcal/mol), whereas MET 181 exhibited moderate but consistent interactions across several targets (−8.1 to −4.6 kcal/mol). In contrast, MET 34 and MET 176 generally showed weaker docking scores (−7.6 to −4.1 kcal/mol). The reference ligands displayed the expected high binding affinities, including SCH772984 for MAPK3 (−14.3 kcal/mol), RLY2608 for PIK3CA (−13.7 kcal/mol), VTX-11e for MAPK1 (−12.2 kcal/mol), Torin2 for MTOR (−11.6 kcal/mol), SYR127063 for ERBB2 (−11.4 kcal/mol), and BE4 for MMP9 (−10.6 kcal/mol). Although the reference ligands consistently outperformed the metabolites, except for MMP9 (−10.6 kcal/mol), several metabolites, particularly MET15, MET28, and MET119, achieved competitive docking scores. Collectively, MET 15 and MET 119 are multi-target binders because of their engagement with several high-priority latency-related hubs, whereas MET 28 and MET 181 retain selective potential. In contrast, MET 34 and MET 176 show weaker binding affinities. Hub targets with higher binding affinities and topological importance in the MET–TAR–PATH network were considered as key targets. The comparative binding poses of the selected complexes are shown in Figure 9B.

### 2.8. Protein–Ligand Interaction Analysis

**Figure 10:**
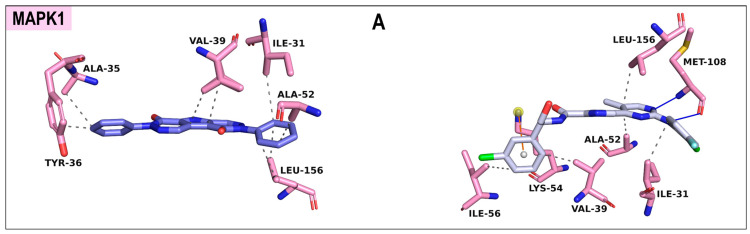

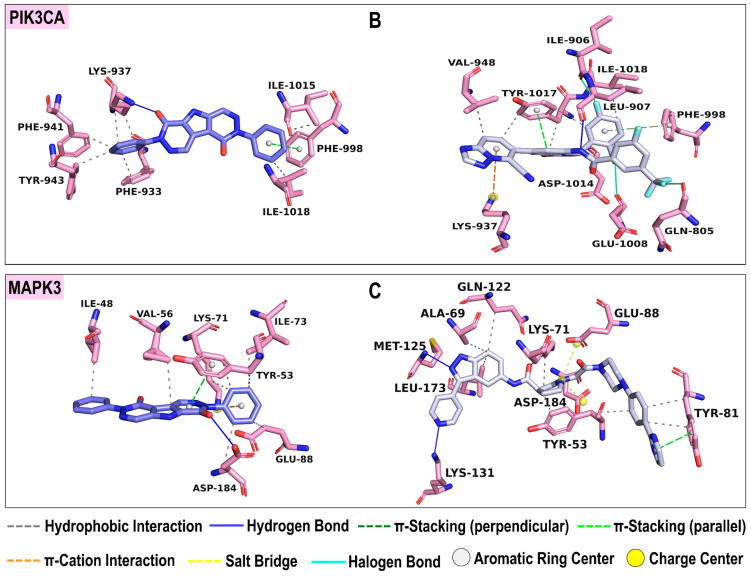

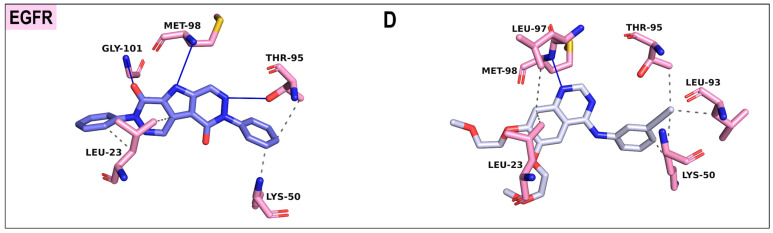

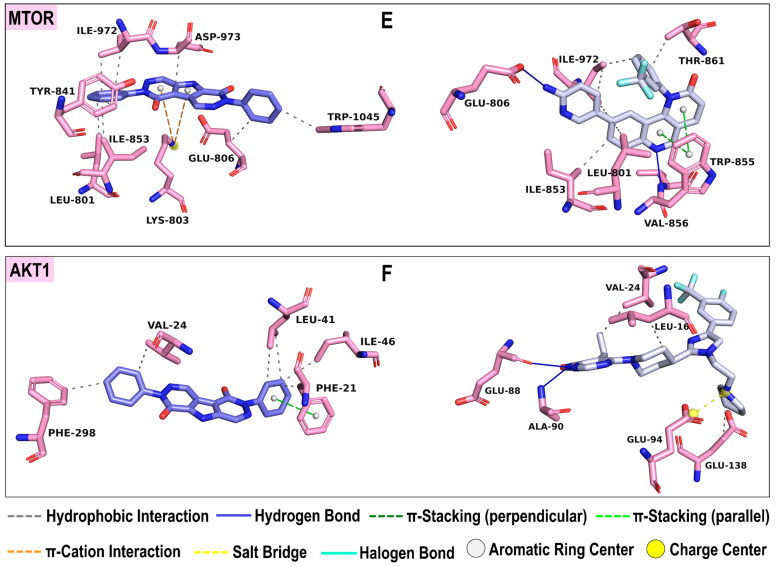
Comparative protein–ligand interaction profiles for MET 15 (**left** panel) and corresponding reference inhibitors (**right** panel) bound to hub proteins. (**A**) MAPK1 complexed with MET 15 and VTX-11e; (**B**) PIK3CA with MET 15 and RLY2608; (**C**) MAPK3 with MET 15 and SCH772984; (**D**) EGFR with MET 15 and Erlotinib; (**E**) MTOR with MET 15 and Torin2; and (**F**) AKT1 with MET 15 and EX4. Each panel shows the key molecular interactions, including hydrophobic contacts (gray dashed lines), hydrogen bonds (blue solid lines), π-stacking (green dashed lines), π-cation interactions (orange dashed lines), halogen bonds (cyan solid lines), and salt bridges (yellow dashed lines). Ligands are shown in stick format, with fungal metabolites in purple and reference drugs in gray. The protein residues are shown as pink sticks. The figures highlight the conserved binding residues and comparable interaction patterns across both the metabolite and reference complexes.

To delineate the molecular underpinnings of ligand binding, detailed interaction profiling was performed (Figure 10). In the MAPK1^MET 15^ complex, hydrophobic interactions were dominant, involving ILE-31, ALA-35, TYR-36, VAL-39, ALA-52, and LEU-156. In comparison, VTX-11e shared hydrophobic contacts (ILE-31, VAL-39, ALA-52, ILE-56, and LEU-156) but extended the binding profile with two hydrogen bonds to MET-108 and a π-cation interaction with LYS-54 (Figure 10A). For PIK3CA binding, MET 15 engaged the hydrophobic residues PHE-933, LYS-937, PHE-941, TYR-943, ILE-1015, and ILE-1018, supported by hydrogen bonding to LYS-937 and π-stacking with PHE-998. RLY2608 displayed a more complex profile with hydrophobic contacts (VAL-948, TYR-1017, and ILE-1018), hydrogen bonds (LEU-907 and ASP-1014), π-stacking (PHE-998 and TYR-1017), a π-cation interaction with LYS-937, and halogen bonds (GLN-805, ILE-906, and GLU-1008) (Figure 10B). For MAPK3 binding, MET 15 formed hydrophobic contacts with ILE-48, TYR-53, VAL-56, LYS-71, ILE-73, GLU-88, and ASP-184, complemented by a hydrogen bond with ASP-184, π-stacking with TYR-53, and a π-cation interaction with LYS-71. SCH772984, in contrast, exhibited a more extensive network comprising hydrophobic interactions (TYR-53, ALA-69, LYS-71, TYR-81, GLN-122, and LEU-173), multiple hydrogen bonds (LYS-71, MET-125, and LYS-131), π-stacking with TYR-81, and salt bridges with GLU-88 and ASP-184 (Figure 10C). For EGFR binding, MET 15 interacted through hydrophobic contacts with LEU-23, LYS-50, and THR-95 and established hydrogen bonds with THR-95, MET-98, and GLY-101. Erlotinib displayed an expanded hydrophobic network (LEU-23, LYS-50, LEU-93, THR-95, and LEU-97) while retaining a hydrogen bond with MET-98, suggesting a broader binding stability (Figure 10D). Furthermore, in the MTOR^MET 15^ complex, there were extensive hydrophobic contacts involving LEU-801, GLU-806, TYR-841, ILE-853, ILE-972, ASP-973, and TRP-1045, with two additional π-cation interactions contributed by LYS-803. Torin 2 displayed a comparable but distinct profile, involving hydrophobic residues (LEU-801, ILE-853, THR-861, ILE-972), hydrogen bonds with GLU-806 and VAL-856, and two π-stacking interactions with TRP-855 (Figure 10E). Finally, for AKT1 binding, MET 15 engaged in hydrophobic interactions with PHE-21, VAL-24, LEU-41, ILE-46, and PHE-298, supported by a π-stacking interaction with PHE-21. In contrast, EX4 formed a more diverse interaction profile, comprising hydrophobic contacts (LEU-16, VAL-24, GLU-138), hydrogen bonds (GLU-88, ALA-90), and a salt bridge with GLU-94 (Figure 10F).

### 2.9. Time-Resolved Thermodynamic Assessment: Structural Conformational Changes and BFE Thermodynamic Profiling

#### 2.9.1. Structural Conformational Changes of Hub Targets

**Figure 11:**
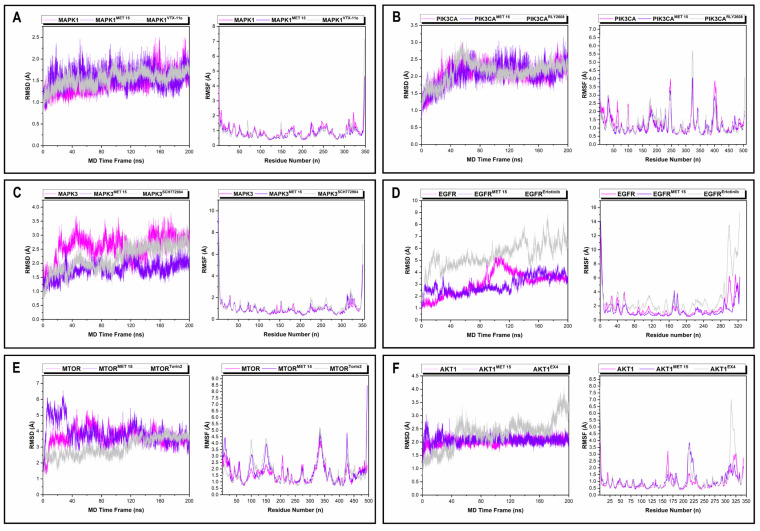
Comparative MD trajectory profiles showing RMSD (**left** panel) and RMSF (**right** panel) of hub proteins (light grey): (**A**) MAPK1, (**B**) PIK3CA, (**C**) MAPK3, (**D**) EGFR, (**E**) MTOR, and (**F**) AKT1 bound to MET 15 (violet) and corresponding reference ligand (magenta) over 200 ns simulation time frame.

Backbone root mean square deviation (RMSD) and per-residue root mean square fluctuation (RMSF) analyses were used to quantify the conformational stability of MAPK1, PIK3CA, MAPK3, EGFR, MTOR, and AKT1 in their apo, MET 15-bound, and reference inhibitor–bound states over the 200 ns MD simulations (Figure 11). The average RMSD values for MAPK1, MAPK1^MET 15^, and MAPK1^VTX−11e^ were 1.47 Å, 1.56 Å, and 1.53 Å, respectively. All three systems equilibrated rapidly within the initial simulation window (~40 ns) and remained stable thereafter. MAPK1^MET 15^ exhibited a compact RMSD distribution comparable to that of MAPK1^VTX−11e^. The average RMSF values for MAPK1, MAPK1^MET 15^, and MAPK1^VTX−11e^ were 0.94 Å, 0.82 Å, and 0.83 Å, respectively. The RMSF profiles of the bound systems showed reduced fluctuations across most residues relative to the apo system, particularly outside the terminal regions (Figure 11A). The average RMSD values for PIK3CA, PIK3CA^MET 15^, and PIK3CA^RLY2608^ was 2.08 Å, 2.08 Å, and 2.12 Å, respectively. The RMSD trajectories of all the complexes overlapped closely throughout the simulation. The average RMSF values of PIK3CA, PIK3CA^MET 15^, and PIK3CA^RLY2608^ were 1.27 Å, 1.16 Å, and 1.25 Å, respectively. Moderate fluctuations were localized to the loop regions, with PIK3CA^MET 15^ exhibiting slightly restricted residue mobility compared to the apo and PIK3CA^RLY2608^ systems (Figure 11B). The average RMSD of MAPK3, MAPK3^MET 15^, and MAPK3^SCH772984^ was 2.57 Å, 1.78 Å, and 2.18 Å, respectively. MAPK3^MET 15^ showed consistently lower RMSD trajectories than both the apo and MAPK3^SCH772984^ systems throughout the simulation. The average RMSF values for MAPK3, MAPK3^MET 15^, and MAPK3^SCH772984^ were 1.01 Å, 1.01 Å, and 1.13 Å, respectively. The RMSF profiles were generally uniform among the three systems, with apo maintaining slightly restrained flexibility across the catalytic domain (Figure 11C). The average RMSD values for EGFR, EGFR^MET15^, and EGFR^Erlotinib^ were 3.08 Å, 2.93 Å, and 5.42 Å, respectively. The average RMSF values for EGFR, EGFR^MET15^, and EGFR^Erlotinib^ were 1.72 Å, 1.40 Å, and 2.93 Å, respectively. EGFR^Erlotinib^ exhibited pronounced RMSD drift and elevated RMSF fluctuations, particularly toward the C-terminal region. In contrast, EGFR^MET15^ showed substantially lower RMSD and reduced RMSF fluctuations, closely mimicking the stability of the apo. MET 15 demonstrated superior conformational restraint compared to the clinical standard (Figure 11D). The average RMSD values for MTOR, MTOR^MET 15^, and MTOR^Torin2^ were 3.65 Å, 3.87 Å, and 2.98 Å, respectively. MTOR^Torin2^ maintained the lowest RMSD trajectories, while MTOR^MET 15^ showed moderate deviation but stable trajectories without abrupt conformational transitions, except for the first 40 ns. The average RMSF values for MTOR, MTOR^MET 15^, and MTOR^Torin2^ were 1.63 Å, 1.77 Å, and 1.75 Å, respectively. Comparable fluctuation patterns were observed across the three systems, with localized peaks corresponding to the flexible loop regions (Figure 11E). The average RMSD values for AKT1, AKT1^MET 15^, and AKT1^EX4^ were 2.02 Å, 2.08 Å, and 2.31 Å, respectively. AKT1^MET 15^ and apo exhibited nearly identical RMSD trajectories and remained stable throughout the simulation. In contrast, AKT1^EX4^ exhibited profound increased RMSD drifts, particularly from 180 to 200 ns. The average RMSF values for AKT1, AKT1^MET 15^, and AKT1^EX4^ were 0.93 Å, 1.07 Å, and 1.12 Å, respectively. AKT1^EX4^ showed elevated RMSF peaks, particularly at the C-terminus region, compared to AKT1^MET 15^ and apo (Figure 11F).

#### 2.9.2. BFE Thermodynamic Profile of Hub Targets

BFE calculations quantified the thermodynamic stability of MET 15 in complex with MAPK1, PIK3CA, MAPK3, EGFR, MTOR, and AKT1 compared to their respective reference inhibitors (Table 5). ΔG_bind_ was decomposed into ΔG_gas_ and ΔG_solv_. For MAPK1 binding, MET 15 exhibited a ΔG_bind_ of −33.66 ± 0.16 kcal/mol, driven by favorable ΔE_vdW_ (−36.95 ± 0.11 kcal/mol) and ΔE_elec_ (−43.08 ± 0.19 kcal/mol) interactions. VTX-11e showed a stronger ΔG_bind_ (−56.08 ± 0.18 kcal/mol), associated with a more favorable ΔG_gas_ (−96.90 ± 0.35 kcal/mol) despite a significant ∆G_solv_ (40.82 ± 0.21 kcal/mol). In the PIK3CA system, MET 15 achieved ΔG_bind_ of −39.03 ± 0.14 kcal/mol, with dominant contributions from ΔE_vdW_ (−50.54 ± 0.08 kcal/mol). In contrast, RLY2608 displayed a substantially stronger ΔG_bind_ of −69.32 ± 0.10 kcal/mol, reflecting the enhanced ΔG_gas_ (−110.69 ± 0.14 kcal/mol). For MAPK3 binding, MET 15 produced a ΔG_bind_ of −25.61 ± 0.14 kcal/mol. Although both ΔE_vdW_ and ΔE_elec_ were favorable, the ΔG_bind_ was weaker than that of SCH772984, which showed a markedly stronger ΔG_bind_ of −88.34 ± 0.14 kcal/mol. In the EGFR complex, MET 15 exhibited a ΔG_bind_ of −31.45 ± 0.10 kcal/mol, primarily supported by ΔE_vdW_ (−44.44 ± 0.08 kcal/mol). Erlotinib demonstrated stronger ΔG_bind_ (−41.90 ± 0.13 kcal/mol), accompanied by higher ΔE_elec_ (−21.40 ± 0.18 kcal/mol) and increased ∆G_solv_ (31.51 ± 0.15 kcal/mol). For MTOR binding, MET 15 recorded a weaker ΔG_bind_ of −21.01 ± 0.16 kcal/mol, with moderate ΔG_gas_ (−45.43 ± 0.26 kcal/mol). Torin2 exhibited a stronger ΔG_bind_ of −34.94 ± 0.11 kcal/mol, consistent with stronger ΔE_vdW_ (−44.73 ± 0.10 kcal/mol) and ΔE_elec_ (−28.16 ± 0.24 kcal/mol). In the AKT1 system, MET 15 displayed the weakest binding among the MET 15 complexes, with ΔG_bind_ of −19.72 ± 0.11 kcal/mol. In contrast, EX4 showed a substantially more favorable ΔG_bind_ of −51.41 ± 0.13 kcal/mol, dominated by a very large ΔE_elec_ (−279.81 ± 0.55 kcal/mol) offset by a high ∆G_solv_ (284.07 ± 0.56 kcal/mol).

## 3. Discussion

This study investigated the therapeutic potential of *Candida albicans* secondary metabolites in modulating HIV pathogenesis through latency dynamics, extending beyond well-known molecules like candidalysin and farnesol to identify novel bioactive scaffolds. Although *C. albicans* has been extensively characterized as an opportunistic pathogen in HIV/AIDS, its metabolites remain largely underexplored as therapeutic agents, despite the emerging interest in fungal metabolite profiling [21,22,23,24,25]. For example, the quorum-sensing secondary metabolite farnesol, which *C. albicans* utilizes in vivo to regulate virulence via immunomodulation, has attracted pharmacological attention for its antimicrobial, anticancer, anti-obesity, anti-inflammatory, and antioxidant properties [26,27]. Notably, Kadian et al. [28] demonstrated that trans, trans-farnesol (TF) alleviated cognitive deficits, oxidative stress, mitochondrial dysfunction, and neuroinflammation in ICV-STZ-induced Alzheimer’s-like rat model. These neuroprotective effects were attributed to TF’s ability to restore antioxidant balance, suppress acetylcholinesterase and TNF-α levels, and normalize mitochondrial enzyme activities, including NADH dehydrogenase and succinate dehydrogenase [28].

In the present study, we employed computational screening, network pharmacology, and molecular modeling to identify *C. albicans* metabolites and their host targets that are implicated in HIV latency regulation. Six candidate metabolites were predicted to exhibit enhanced intracellular permeability and a high synthetic feasibility. Among them, three metabolites (MET 28, MET 176, and MET 181) demonstrated BBB permeability, which is pharmacologically advantageous given the role of CNS reservoirs in HIV persistence [2]. However, two metabolites (MET 28 and MET 119) may present drug–drug interaction (DDI) liabilities when co-administered with antiretroviral therapies metabolized by CYP3A4. MET 119 presents an “Absorption Paradox.” It has low GI absorption but is also soluble. This suggests that MET 119 may be better suited for intravenous (IV) administration rather than oral administration, which is common for intensive HIV reservoir-purging therapies. Toxicological profiling revealed generally favorable safety characteristics, with minimal organ and systemic risks observed, except for mild neurotoxicity alert associated with MET 15. Therefore, targeted delivery or structural optimization strategies can help reduce its toxicity while maintaining efficacy. Overall, the ADMET properties indicated acceptable solubility, drug-likeness, and manageable safety margins for most metabolites. Notably, BBB permeability and neurotoxicity predictions enabled the identification of candidate compounds with the potential to safely penetrate anatomical sanctuaries while maintaining systemic activity against peripheral HIV reservoirs. Apart from farnesol (MET 181), the remaining compounds were isolated from methanolic extracts of *C. albicans* cultures and possess diverse structural scaffolds that merit further exploration. The methanolic extract was found to exhibit broad-spectrum antibacterial potential [21]. These findings suggest that the metabolite repertoire of *C. albicans* could be significantly expanded through solvent variation and optimized culture conditions, consistent with the one strain, many compounds (OSMAC) approach successfully employed in endophytic fungi for the discovery of novel bioactive molecules [29]. Furthermore, high-throughput metabolomics strategies could further unlock the “dark metabolome” of *C. albicans* for HIV research. The metabolites were mapped to 369 human targets, of which 176 overlapped with HIV latency-associated genes. These overlapping targets represent potential mediators through which *C. albicans* metabolites influence viral persistence. Network topology analysis revealed dense interconnectivity, reflecting the coordinated regulation among latency-related targets. This “small-world network” implies that information (or signals) can spread very quickly across the network, which is biologically significant for HIV latency regulation. The comparatively high DC values observed for these metabolites suggest biological significance, as their extensive interactions with multiple target proteins imply a potential role in modulating host–hijacked HIV–signaling dynamics and network stability.

The 20 hub genes identified in the PPI network represent central regulators of cellular signaling and transcriptional control that are intricately linked to HIV latency maintenance and reactivation. They include kinases (AKT1, EGFR, MAPK3, GSK3B, MTOR, ERBB2, JAK2, MAPK1, and PIK3CA), enzymes (PTGS2, MMP9, MDM2, and PARP1), transcription factors (STAT3 and RELA), nuclear receptors (ESR1 and PPARG), epigenetic regulators (SIRT1), and signaling molecules (TNF and CCND1). Their high DC underscores their pivotal role as network regulators that can potentially govern HIV latency modulation. The polypharmacological interaction profile of the metabolites further supports the hypothesis that *C. albicans* secondary metabolites may exert synergistic effects by simultaneously targeting multiple latency-associated proteins. Among the kinases, AKT1 and MTOR serve as pivotal mediators of cellular survival and metabolic homeostasis, influencing both HIV transcription and reservoir viability. Modulation of the AKT/MTOR axis has been shown to alter latent-reservoir stability and reactivation dynamics. Similarly, MAPK1 and MAPK3 (ERK2 and ERK1) regulate transcriptional programs essential for proviral activation, as ERK-mediated signaling modulates activator protein 1 (AP-1) and positive transcription elongation factor b (P-TEFb) activities, thereby affecting chromatin accessibility and elongation of integrated proviruses [30]. Growth factor–related kinases, including EGFR, ERBB2, PIK3CA, and GSK3B, converge on PI3K/AKT signaling nodes that define T-cell activation thresholds and NF-κB signaling balance, which are key determinants of HIV proviral transcription [30,31,32]. Similarly, the JAK/STAT axis influences cytokine-mediated control of latency, and inhibition of JAK2 represses viral reactivation, whereas hyperactivation of STAT3 supports immune dysfunction and the persistence of latent infection [33,34]. Enzymatic hubs, such as MMP9, PTGS2, MDM2, and PARP1, integrate extracellular remodeling, inflammatory signaling, and DNA damage responses. Notably, PARP1 functions as a transcriptional co-activator of NF-κB and interacts with heat shock factor 1 (HSF1) to promote latent proviral reactivation and viral gene expression [35,36]. MDM2, a negative regulator of p53, modulates cell survival and may indirectly sustain latent reservoirs [37]. Transcriptional and epigenetic regulators RELA (NF-κB p65) and SIRT1 directly orchestrate proviral latency control, with SIRT1 deacetylating RELA at Lys310 to dampen NF-κB transcriptional activity and maintain latency [38]. Conversely, TNF serves as a potent latency-reversing cytokine by inducing NF-κB–mediated proviral transcription [39]. Nuclear receptors ESR1 and PPARG modulate host transcriptional and inflammatory pathways that shape HIV transcriptional competence [40,41]. Finally, CCND1, which governs the G_1_/S phase transition via CDK4/6 activation, remains low in quiescent CD4^+^ T cells, the major latent HIV reservoir, thereby reinforcing a non-permissive state for viral transcription [42,43].

GO enrichment highlights functional themes that mechanistically link *Candida albicans* metabolites to HIV latency control. Within the BP category, the enrichment of responses to oxygen- and nitrogen-containing compounds underscores the pivotal role of oxidative stress in NF-κB–dependent HIV reactivation [44,45]. CC enrichment of plasma membrane microdomains, specifically caveolae and lipid rafts, is particularly notable, as these dynamic regions orchestrate signaling assemblies, such as EGFR/PI3K/AKT and MAPK/ERK complexes, that drive transcriptional reprogramming essential for proviral activation and reservoir maintenance [46]. Moreover, HIV-1 exploits lipid rafts during viral entry, assembly, and budding, thereby co-opting the host membrane architecture for persistence [47]. In the MF category, significant enrichment for protein tyrosine kinase and serine/threonine kinase activities reflects the dominance of MAPK and PI3K/AKT/MTOR signaling hubs identified within the PPI network. ERK1/2 (MAPK3/MAPK1) signaling regulates Tat-dependent HIV transcription [48], while MTOR signaling modulates metabolic and translational landscapes that determine reservoir inducibility [49]. Moreover, the enrichment of transcription co-regulator binding and catalytic activity further corroborates the roles of SIRT1, PARP1, and NF-κB as critical regulators of proviral chromatin and transcriptional competence. SIRT1 deacetylates both Tat and RELA, thereby fine-tuning their transactivation potential [38,50], whereas PARP1 contributes to transcriptional regulation and latency dynamics through its co-activator functions [35]. NF-κB also cooperates with AP-1 downstream of T-cell receptor (TCR) signaling to trigger latent proviral reactivation [51].

KEGG pathway enrichment analysis highlights that *Candida albicans* secondary metabolites are predicted to target highly conserved growth and pro-survival signaling circuits that overlap extensively with mechanisms governing HIV latency and reactivation. Among the enriched pathways, the VEGF signaling pathway (hsa04370) was the most overrepresented, underscoring the convergence of angiogenic and viral regulatory networks. VEGF-mediated activation of the PI3K/AKT and MAPK cascades is known to feed into the NF-κB and HIF-1α pathways, both of which regulate HIV-1 transcriptional activation and latent reservoir maintenance [52]. The enrichment of several cancer-related pathways, including non-small cell lung cancer (hsa05223), pancreatic cancer (hsa05212), acute myeloid leukemia (hsa05221), and prostate cancer (hsa05215), further emphasizes the mechanistic parallels between tumorigenic signaling and HIV persistence [53]. These networks converge on MAPK/ERK and PI3K/AKT signaling hubs that fine-tune T-cell activation thresholds, chromatin accessibility, and transcriptional elongation of latent proviruses [54]. Consistent with this, therapeutic modulation of the PI3K/AKT axis has been proposed as a strategy to destabilize latent HIV reservoirs [30]. Ten of the enriched pathways identified in this study have previously been linked to HIV latency regulation, reactivation, or immune evasion. The PI3K–Akt signaling pathway (hsa04151) and pathways in cancer (hsa05200) exhibit the highest gene participation, reinforcing the interconnectedness of oncogenic and viral persistence mechanisms [34,38,54,55]. The Ras signaling (hsa04014) and focal adhesion (hsa04510) pathways interact with the MAPK/ERK cascade to promote transcriptional activation and intercellular viral transmission. Focal adhesions facilitate LFA-1–ICAM–mediated viral spread and contribute to reservoir establishment [56], whereas Ras signaling activates the Raf/MEK/ERK cascade, enhancing HIV transcription through RBF-2–dependent regulation of the LTR [54]. PD-L1 expression and PD-1 checkpoint signaling (hsa05235) also featured prominently, reflecting their central role in maintaining latency via T-cell exhaustion and functional anergy. The engagement of PD-1 suppresses TCR signaling and restricts proviral reactivation, whereas PD-1 blockade enhances latency reversal ex vivo [57,58]. The HIF-1 signaling pathway (hsa04066), downstream of PI3K/AKT and MAPK activation, modulates HIV transcription through metabolic reprogramming under hypoxic conditions: HIF-1α enhances LTR activity, while HIF-2α suppresses viral transcription within hypoxic lymphoid niches [59,60]. Calcium (hsa04020) and cAMP (hsa04024) signaling pathways are canonical regulators of latency reversal. Calcium influx activates the calcineurin–NFAT axis, while elevated cAMP stimulates the PKA/CREB cascade, both of which enhance proviral transcription and have been leveraged in “shock and kill” latency-reversal strategies [61,62]. The enrichment of microRNAs in cancer (hsa05206) and sphingolipid signaling (hsa04071) further underscores the epigenetic and lipid-mediated modulation of HIV persistence, respectively. MicroRNAs such as miR-28, miR-125b, and miR-150 suppress HIV transcripts in resting CD4^+^ T cells [63], while sphingolipid metabolites such as sphingosine-1-phosphate (S1P) activate NF-κB to promote reactivation, and analogs such as FTY720 suppress viral replication [64,65]. Sphingolipids, including sphingosine and sphinganine, are essential structural components of cellular membranes and key intermediates in lipid metabolism pathways. Together with cholesterol, they contribute to the formation of lipid raft microdomains that organize membrane signaling and host–pathogen interactions [66]. Sphingolipid metabolism plays an important role in viral infection by influencing viral attachment, membrane fusion, intracellular trafficking and budding. Several viruses, including HIV, influenza virus, rhinovirus, and SARS-CoV-2, exploit sphingolipid-rich membrane domains to facilitate cellular entry and replication [67]. Mechanistically, sphingosine and sphinganine alter membrane lipid organization and lipid raft stability, affecting virus–receptor interactions and membrane fusion. In addition, sphingosine serves as a precursor for S1P, a signaling lipid that regulates pathways involved in endocytosis, inflammation, and cytoskeletal dynamics, which can influence viral entry and replication [68]. Collectively, these findings suggest that *C. albicans* metabolites may influence HIV latency through multilayered mechanisms, spanning transcriptional regulation, metabolic control, and immune modulation, highlighting their polypharmacological potential for targeting cellular programs that sustain viral persistence. The enrichment of these cancer-related pathways reflects the signaling convergence within multifunctional host pathways that HIV exploits rather than disease-specific oncogenic mechanisms.

The integrated MET–TAR–PATH network delineates a central cell growth and survival signaling module anchored by PIK3CA, MAPK1, MAPK3, AKT1, EGFR, and MTOR, linking *Candida albicans* metabolites to multiple HIV latency-relevant pathways. The prominence of these hubs is mechanistically significant, as PI3K/AKT/MTOR and MAPK/ERK cascades are master regulators of cellular metabolism, transcriptional reprogramming, and survival processes that collectively govern proviral maintenance. While these hub proteins are often described as oncogenic in cancer contexts, they function as key metabolic and signaling regulators in quiescent T-cells. Their modulation in this setting is not aimed at oncogenesis but rather at controlling pathways that influence HIV latency, effectively “flipping the switch” of the latent provirus. Metabolites such as MET 28 appear to exert polypharmacological, multi-node modulation capable of altering the latency–reactivation equilibrium. Lower-degree nodes, including SIRT1, PPARG, PARP1, and TNF, likely represent context-dependent regulatory axes encompassing chromatin remodeling, DNA repair, and cytokine-mediated inflammatory responses, processes that complement but are less central to the canonical KEGG-defined latency framework. The strong multitarget binding affinities observed for MET 15, MET 28, and MET 119 further reinforce their potential as broad-spectrum modulators of latency. Their stable engagement with MAPK1/3, PIK3CA, AKT1, MTOR, and EGFR implies coordinated regulation of both MAPK/ERK and PI3K/AKT/MTOR signaling—two convergent axes that dictate the transcriptional and survival landscape of HIV-infected cells. Sustained PI3K/AKT/MTOR activation maintains latency by promoting cellular survival and repressing viral transcription, whereas MAPK/ERK signaling mediates the transcriptional reactivation of latent proviruses [30,48,49]. Thus, the engagement of these kinases by *C. albicans* metabolites could destabilize latency-supportive signaling, shifting the balance toward proviral reactivation or immune clearance. Notably, the predominance of hydrophobic and π–stacking interactions observed in molecular docking mirrors those exploited by the optimized kinase inhibitors, suggesting favorable druggability and bioavailability profiles. Collectively, the polypharmacological binding patterns of key metabolites, particularly MET 15 and MET 119, underscore their potential to modulate multiple latency-supportive networks concurrently, thereby providing a rational scaffold for the development of next-generation latency-modulating therapeutics.

MD simulations and MM/GBSA analyses provided thermodynamic support for the network-level predictions by demonstrating that MET 15 forms stable complexes with multiple kinase hubs without inducing large-scale structural perturbations. *In silico* studies of kinase–ligand systems have shown that the preservation of native conformational dynamics is a desirable feature for compounds intended to modulate host signaling pathways rather than fully abrogating kinase function [69,70]. The dominance of van der Waals contributions in MET 15 binding, accompanied by moderate electrostatic interactions, is consistent with the binding modes reported for several ATP-competitive kinase inhibitors, in which hydrophobic packing within conserved kinase pockets represents a major energetic driver. This stable binding attests to the excellent shape complementarity of MET 15 with the hydrophobic pockets of these kinases. Importantly, MET 15 exhibited moderate but consistent binding across multiple hubs, supporting its classification as a polypharmacological modulator rather than a high-affinity single-target inhibitor. From a biological perspective, the simultaneous engagement of the PI3K/AKT/MTOR and MAPK/ERK axes is notable, given their documented roles in regulating cellular survival and inducible HIV transcription. Latency is increasingly understood as a network-stabilized state rather than a single-pathway phenomenon, suggesting that moderate multi-node perturbation may be more effective than selective inhibition [38,54]. The key hub genes (PIK3CA, MAPK1/3, EGFR, MTOR, and AKT1) are widely recognized signaling hubs and may appear as high-degree nodes in PPI networks due to study and annotation bias [71,72]. To address this challenge, their prioritization in this study was not based solely on network centrality but also on their intersection with HIV latency-associated host factors, convergence across enriched KEGG pathways, and subsequent structural validation through molecular docking and MD simulation [20]. Collectively, the systems-level coherence strengthens the biological plausibility of fungal secondary metabolites as unconventional but mechanistically useful scaffolds for host-directed HIV latency intervention.

The candidate metabolites identified in this study represent structurally diverse chemical classes, including nitrogen-rich heterocycles, quinoline derivatives, sulfonamides, fatty acids, and terpenoid alcohols. Many compounds containing these pharmacophores have been associated with antiviral activity or with modulation of host signaling pathways that regulate viral latency. For example, MET 15 contains a fused nitrogen-rich heterocyclic system combining pyridazine and pyrrolo motifs. Pyrrolo-pyrimidine analogs have demonstrated antiviral activity against flaviviruses, such as Zika and dengue viruses [73]. In addition, fused nitrogen heterocycles share structural similarities with pharmacophores found in bromodomain and extraterminal domain (BET) inhibitors, which influence HIV transcription through pathways involving BRD4 and P-TEFb [74]. MET 28, a quinoline derivative, belongs to a class of compounds with broad antiviral properties, including activity against the dengue virus, SARS-CoV-2, and HIV [75]. Quinoline-based compounds, such as AV6 derivatives, have also been shown to reactivate latent HIV in cellular models and to synergize with histone deacetylase (HDAC) inhibitors, thereby enhancing viral transcription without inducing excessive T-cell activation [76]. MET 34 contains a pyrrolidine ring and tertiary amide functional groups, structural motifs commonly found in enzyme-binding ligands. Pyrrolidine-containing compounds have been reported to interact with viral enzymes, including proteases and polymerases [77]. MET 119 combines pyrimidine and sulfonamide moieties. Pyrimidine derivatives can interfere with nucleic acid synthesis [78], while sulfonamide-containing molecules interact with epigenetic enzymes such as HDACs [79]. MET 176 is a long-chain unsaturated fatty acid, a class of molecules that can inhibit HIV entry or fusion with host cells [80]. Fatty acids may also modulate host metabolic signaling pathways through nuclear receptors, such as peroxisome proliferator-activated receptors (PPARs), which may influence viral persistence [81]. Finally, MET 181, a sesquiterpene alcohol, has been reported to modulate host inflammatory signaling pathways, such as NF-κB, which plays a key role in HIV transcriptional activation [82]. Collectively, the structural features and previously reported biological activities of these compounds provide a plausible basis for their predicted interactions with host targets involved in HIV latency, thereby supporting their prioritization for further experimental investigation. The identification of latency-modulating metabolites from *Candida albicans* should not be interpreted as implying that fungal colonization or infection provides therapeutic benefits for PLWH. Rather, consistent with established natural-product drug discovery approaches, microorganisms, including opportunistic and/or pathogenic species, produce chemically diverse secondary metabolites for pharmacological development following isolation, structural optimization, and experimental validation [83,84].

### Study Limitations

This study offers an integrative systems-level perspective on the potential of *Candida albicans* metabolites to modulate HIV-1 latency; however, several limitations should be acknowledged. These findings are based on computational predictions from network pharmacology, molecular docking, and dynamics simulations, which, while robust, remain theoretical and require experimental confirmation. *In silico* approaches cannot fully capture the multifactorial complexity of HIV latency regulation, including chromatin remodeling, transcriptional feedback, and immune microenvironmental influences. PPI network outcomes are constrained by existing database annotations and potential knowledge biases. Additionally, pathway enrichment analyses are inherently constrained by database structures and annotation biases. The apparent over-representation of molecular targets within the KEGG pathways in cancer map (hsa05200), for example, largely reflects the aggregation of extensively characterized oncogenic signaling cascades rather than a direct or exclusive association with cancer-specific pathogenic drivers. Many of these pathways, such as PI3K/AKT/MTOR, MAPK/ERK, and EGFR signaling, function as multifunctional host regulatory hubs that are equally central to immune signaling, cellular survival, and HIV-1 latency maintenance. Consequently, pathway enrichment in cancer-associated maps should be interpreted as evidence of signaling convergence and network centrality, rather than disease specificity. Furthermore, the pharmacokinetic behavior, bioavailability, and cytotoxicity profiles of the identified metabolites remain uncharacterized. Despite these limitations, the multi-target and pathway-convergent insights generated in this study provide a rational foundation for future experimental and translational exploration of fungal-derived latency-modulating scaffolds.

## 4. Materials and Methods

### 4.1. Collection of Candida albicans Secondary Metabolites

A comprehensive library of secondary metabolites derived from *Candida albicans* was compiled through an extensive literature review conducted between 3–10 June 2025. Searches were performed across major scientific databases, including PubMed, Scopus, and Google Scholar, using relevant keywords such as “*Candida albicans*,” “secondary metabolites,” “natural products,” and “bioactive compounds.” Only metabolites characterized using gas chromatography-mass spectrometry (GC-MS) or nuclear magnetic resonance (NMR) metabolomics were included to ensure reliable chemical identification [21,22,23,24,25]. A total of 185 unique secondary metabolites were identified. Their chemical structures were retrieved in simplified molecular input line entry system (SMILES) format from the PubChem database (https://pubchem.ncbi.nlm.nih.gov/, accessed on 10 June 2025).

### 4.2. Evaluation of ADMET Properties

To prioritize pharmacologically viable latency-modulating candidates, all 185 curated *Candida albicans* metabolites (MET 1–MET 185) were subjected to ADMET evaluations (Supplementary Table S1). Drug-likeness and pharmacokinetic properties were predicted using SwissADME (http://www.swissadme.ch/, accessed on 11 June 2025). Toxicity assessment was performed using the ProTox-3.0 platform (https://tox.charite.de/protox3/, accessed on 11 June 2025). The screening workflow employed a rigorous multiparametric filter to ensure the selection of compounds with favourable pharmacokinetic and safety profiles. First, compounds were retained if they met the acceptable MW range (200 ≤ MW ≤ 500 g/mol) for OB. Compliance with Lipinski’s Ro5 was strictly enforced, retaining only those compounds with zero violations. Solubility was assessed using the estimated solubility (ESOL) LogS model with a minimum threshold of –6.0, which is indicative of at least moderate aqueous solubility. Lipophilicity was evaluated using the implicit LOGP (iLOGP) model, with a cutoff value of ≤ 5 to indicate acceptable membrane permeability and bioavailability. Additionally, compounds with a TPSA ≤ 140 Å^2^ were considered likely to possess favourable permeability characteristics. GI absorption and BBB permeability are essential criteria for prioritizing compounds capable of targeting both peripheral and central HIV reservoirs. Furthermore, to minimize the risk of nonspecific interactions, metabolites containing PAINS were excluded. Additionally, SA scores were considered, and only compounds with SA < 6 were retained, reflecting the reasonable feasibility of the chemical synthesis [85]. Toxicity profiling included the prediction of LD_50_, toxicity class assignment, organ-specific toxicities (hepatotoxicity, neurotoxicity, nephrotoxicity, respiratory toxicity, cardiotoxicity, and carcinogenicity), and toxicity endpoints such as immunotoxicity, mutagenicity, and cytotoxicity. Compounds are categorized into six toxicity classes based on their predicted LD_50_ values: Classes I (highly toxic), Class II (moderately toxic), Class III (toxic), Class IV (harmful), Class V (may be harmful), and Class VI (non-toxic) [86].

### 4.3. Human Target Prediction of Candida albicans Secondary Metabolites

The six secondary metabolites that passed ADMET screening were subjected to target prediction using SwissTargetPrediction (http://www.swisstargetprediction.ch/, accessed on 13 June 2025), with the species parameter set to “*Homo sapiens*” and a probability threshold > 0. This platform predicts the most probable macromolecular targets of bioactive compounds based on two-dimensional (2D) and three-dimensional (3D) molecular similarity to known ligands with experimentally validated binding data [87]. Its application is particularly relevant to this study, given the limited experimental data available for fungal metabolites in the context of HIV-1 latency. TargetNet (probability ≥ 0.7) was employed to enhance the prediction coverage. TargetNet uses quantitative structure-activity relationship (QSAR) models and machine learning (ML) algorithms to predict high-confidence targets, making it suitable for underexplored natural products. The union of predicted targets from both platforms were cross-referenced with the human proteome using the UniProt Knowledgebase (UniProtKB) (https://www.uniprot.org/) to ensure proper annotation.

### 4.4. Collection of Candida albicans-HIV Latency-Associated Genes

Human genes annotated in HIV latency were retrieved from the GeneCards database (https://www.genecards.org/) using the keyword “HIV latency,” accessed on 14 June 2025. Only Genes with a relevance score > 0 were compiled. GeneCards is a comprehensive bioinformatics resource that integrates genomic, transcriptomic, proteomic, and disease-related data to provide high-confidence gene function associations. Its built-in relevance scoring system facilitates the prioritization of genes that are most strongly linked to HIV latency mechanisms [88]. To identify potential interactions between *Candida albicans* and HIV-latency-related human pathways, overlapping genes were identified using Venny 2.1.0 (https://bioinfogp.cnb.csic.es/tools/venny/, accessed on 14 June 2025). The analysis compared *C. albicans*-related gene targets (derived from prior target mapping) with the HIV latency-associated gene set obtained from the GeneCards database. Overlapping genes were annotated and standardized using UniProtKB (https://www.uniprot.org/) to ensure consistent nomenclature for downstream analyses.

### 4.5. MET-TAR Network Construction

To elucidate the multitarget interactions of *Candida albicans* secondary metabolites with HIV latency-associated host proteins, a MET–TAR network was constructed using Cytoscape v3.10.3 (https://cytoscape.org/, accessed on 15 June 2025). In this network, nodes represent secondary metabolites or the overlapping protein targets, and edges represent their interactions. The topological characteristics of the network were analyzed using the NetworkAnalyzer tool v4.5.0 in Cytoscape to compute metrics such as DC, BC, and CC [89].

### 4.6. PPI Network Analyses

To investigate the functional interactions among HIV-latency-associated host targets modulated by *Candida albicans* metabolites, a PPI network was constructed. Overlapping genes between the predicted targets of the secondary metabolites and HIV latency-related gene targets were retrieved from the STRING database v12.0 (https://string-db.org/, accessed on 16 June 2025), with the search restricted to “*Homo sapiens*.” A minimum interaction confidence score of 0.400 was applied, and associations with FDR > 5% were excluded to enhance network reliability. The resulting interaction network was visualized and analyzed in Cytoscape v3.10.3 [89]. To prioritize key regulatory genes, hub nodes were identified based on DC using the CytoHubba plugin v0.1 [90]. DC quantifies the number of direct connections each node has within the network. Nodes with higher degree values were considered more influential, as they represent highly connected proteins capable of exerting broad control over latency-associated signaling. The top 20 hub genes with the highest degree scores were selected as candidate targets for therapeutic exploration in the present study.

### 4.7. GO and KEGG Pathway Annotations

To comprehensively explore the biological roles and signaling pathways associated with HIV latency-related targets modulated by *Candida albicans* metabolites, functional enrichment analysis was conducted using ShinyGO v0.82 (https://bioinformatics.sdstate.edu/go/, accessed on 17 June 2025) [91]. Analysis was performed for overlapping genes between the predicted metabolite targets and HIV latency-associated host genes. All analyses were carried out under the species parameter “*Homo sapiens*.” GO analysis categorized functional annotations into BP, CC, and MF. KEGG enrichment analysis was used to identify the signaling pathways that are potentially modulated by these genes. To ensure robustness, the results were filtered using an FDR of < 0.05. Enriched terms were ranked by FDR-adjusted *p*-values and FE, and the top 30 terms from each GO category and KEGG pathway dataset were visualized as bubble plots, representing the gene count and statistical significance (−log_10_[FDR]).

### 4.8. Construction of MET–TAR–PATH Network

To delineate the multiscale regulatory interactions between *Candida albicans* metabolites, host target genes, and HIV latency-associated signaling pathways, a composite MET–TAR–PATH network was constructed using Cytoscape v3.10.3. This network was generated by integrating the MET–TAR associations with target–pathway (TAR–PATH) relationships using the six candidate secondary metabolites of *C. albicans*, the top 20 hub genes, and the top 30 KEGG pathways. In the network, nodes correspond to either metabolites, target genes, or pathways, and edges represent the predicted interactions or functional associations. Topological characteristics were evaluated using the NetworkAnalyzer plugin v4.5.0 in Cytoscape, with DC as the primary metric to quantify node connectivity and identify key hubs [89].

### 4.9. Molecular Docking Calculations

Molecular docking calculations were performed to evaluate the binding potential of the six candidate *Candida albicans* secondary metabolites against 11 key targets with topological importance from MET–TAR–PATH analysis. 3D structures of the target proteins were obtained from the RCSB Protein Data Bank (PDB) (https://www.rcsb.org/, accessed on 20 June 2025). The selection criteria included: (i) X-ray crystal structures resolved at ≤ 3.0 Å, (ii) origin from *Homo sapiens*, (iii) presence of co-crystallized ligands, and (iv) selection of the highest-resolution structure when multiple entries existed. RELA and CCND1 structures lack co-crystallized ligands; therefore, their binding sites were predicted using CASTp 3.0 (http://sts.bioe.uic.edu/castp/, accessed on 20 June 2025), and validated using published structural evidence [92]. 3D ligand structures were retrieved from the PubChem database, energy-minimized using the General AMBER Force Field (GAFF) v1.19, assigned Gasteiger partial charges, and saved in PDBQT format using UCSF Chimera v1.19. Protein structures were also prepared using UCSF Chimera v1.19, which included the removal of water molecules, separation of non-essential heteroatoms, addition of polar hydrogens, and modeling of missing side chains using Modeller v10.5 [93]. Docking simulations were performed using AutoDock Vina v1.1.2 [94]. For protocol validation, the redocking of native ligands was performed to assess docking accuracy by calculating the RMSD values between the docked and experimental poses (Supplementary Figure S3). Binding sites were defined based on the coordinates of the co-crystallized ligands or the predicted active pockets of the apo structure. Docked poses were analyzed using the protein-ligand interaction profiler (PLIP), which details non-covalent interactions [95].

### 4.10. MD Simulations

To assess the structural stability and dynamic behavior of the top protein–ligand complexes, MD simulations were performed using the AMBER18 package (University of California, San Francisco [UCSF], USA) with the PMEMD.CUDA module for GPU-accelerated computation [96]. This simulation approach enables the evaluation of atomic motion, intermolecular interactions, and conformational changes over time. The ligands were parameterized using ANTECHAMBER with RESP-generated charges and GAFF. The protein structures were preprocessed using pdb4amber, and each complex was solvated in a TIP3P water box with a 12 Å buffer and neutralized with Na^+^ or Cl^−^ counterions [97]. Energy minimization was first conducted using restrained minimization (500 kcal/mol/Å^2^ restraint) for 2500 steps, followed by unrestrained minimization for 200 steps. Heating was carried out from 0 to 310 K over 50 ps under the NVT ensemble using a Langevin thermostat, with 10 kcal/mol/Å^2^ positional restraints on the solute. This was followed by pressure equilibration under the NPT ensemble at 1 atm using the Berendsen barostat. SHAKE constraints were applied to all hydrogen-containing bonds, and production runs were performed for 200 ns with a 2 fs time step under periodic boundary conditions. Post-simulation analysis was performed using the CPPTRAJ module to compute the RMSD and RMSF. Trajectories were visualized and inspected using UCSF Chimera v1.19 and PyMOL v3.1.4, and plots were generated using Origin 2018 software [20,98].

### 4.11. BFE Calculations

BFE calculations were performed using the molecular mechanics/Generalized Born surface area (MM/GBSA) method [99]. This approach was implemented using the MMPBSA.py module in the AMBER18 software suite, which couples molecular mechanics with an implicit solvent model to provide dynamic trajectory-based estimations of binding affinities that are superior to static docking scores [100,101]. For each protein–ligand complex, 50,000 frames were uniformly sampled from 200 ns MD trajectories to ensure robust averaging. The BFE (ΔG_bind_) was computed using the following thermodynamic cycle:

(1) $${∆G}_{bind}=G_{complex}-G_{receptor}-G_{ligand}$$

The total energy is further decomposed into the following components:

(2) $${∆G}_{bind}=E_{gas}+G_{sol}-T∆S$$

(3) $$E_{gas}=E_{int}+E_{vdw}+E_{ele}$$

(4) $$G_{sol}=G_{GB}+G_{SA}$$

(5) $$G_{SA}=\gamma SASA$$

The gas-phase energy (E_gas_) was contributed by the internal (E_int_), van der Waals (E_vdw_), and electrostatic (E_ele_) terms. The solvation free energy (G_sol_) was contributed by the polar term (G_GB_) from the Generalized Born (GB) model and the nonpolar term (G_SA_) from the solvent-accessible surface area (SA) model, with the surface tension constant (γ) set at 0.0072 kcal/mol/Å^2^. The entropic contribution (T∆S) was calculated using normal mode analysis.

## 5. Conclusions

This study provides the first integrative systems-level insight into how *Candida albicans* secondary metabolites may influence HIV-1 latency through multitarget engagement of interconnected signaling networks. Through network pharmacology, molecular docking, and dynamics interaction analyses, the metabolites MET 15, MET 28, and MET 119 were identified as promising polypharmacological modulators targeting key latency-associated hubs, such as PIK3CA, MAPK1, MAPK3, AKT1, EGFR, and MTOR. The enrichment of KEGG pathways, including PI3K/AKT, MAPK, PD-1/PD-L1 checkpoint, and HIF-1 signaling, underscores the mechanistic plausibility of these metabolites in modulating cellular survival and transcriptional programs that sustain proviral persistence. By uncovering the potential intersections between fungal metabolism and HIV latency regulation, we identified MET 15 as a top-ranked metabolite and an unexplored reservoir of latency-modulating scaffolds with potential translation in HIV cure research.

The dual nature of *C. albicans* as both an opportunistic pathogen and a reservoir of bioactive secondary metabolites presents a unique paradox for antiviral drug development. Our findings suggest that metabolites such as MET 181 (farnesol) are not merely passive byproducts of fungal metabolism but may actively shape the host’s signaling landscape. By modulating key pathways, including the PI3K/AKT/MTOR and MAPK/ERK axes, these compounds could potentially be leveraged as either “block and lock” agents to reinforce HIV latency or as LRAs to support “shock and kill” strategies, depending on their specific agonistic or antagonistic activities.

Future studies should experimentally validate the identified metabolites using latency models such as J-Lat or primary CD4^+^ T-cell systems to confirm their effects on proviral transcription. Mechanistic assays integrating transcriptomic and proteomic profiling are recommended to delineate the signaling nodes through which these metabolites influence the PI3K/AKT, MAPK, and NF-κB pathways. In addition, structure–activity relationship (SAR) studies and rational analog design can optimize their potency, selectivity, and pharmacokinetic properties. Finally, combining metabolomics with host–pathogen interaction datasets may clarify whether these fungal metabolites act synergistically with known LRAs or immune modulators.

## Figures and Tables

**Table 1:** Drug-likeness, pharmacokinetic, and toxicity profiles of the six candidate *Candida albicans* secondary metabolites predicted using SwissADME (http://www.swissadme.ch/, accessed on 11 June 2025) and ProTox-3.0 (https://tox.charite.de/protox3/, accessed on 11 June 2025).

Parameter	MET 15	MET 28	MET 34	MET 119	MET 176	MET 181
Chemical Name	2,7-Diphenyl-1,6-dioxopyridazino [4,5:2′,3′]pyrrolo[4′,5′-d]pyridazine	5-Bromo-8-[(4-hydroxybenzylidene)amino]quinoline	Acetamide, N-methyl-N-[4-[2-acetoxymethyl-1-pyrrolidyl]-2-butynyl]-	N-(4,6-Dimethyl-2-pyrimidinyl)-4-(4-nitrobenzylideneamino)benzenesulfonamide	Z-8-Methyl-9-tetradecenoic acid	Farnesol
PubChem CID	135833395	582017	580233	542809	5364410	3327
Canonical SMILES	O=c1n(ncc2c1[nH]c1c2c(=O)n(nc1)c1ccccc1)c1ccccc1	Oc1ccc(cc1)C=Nc1ccc(c2c1nccc2)Br	CC(=O)OCC1CCCN1CC#CCN(C(=O)C)C	Cc1nc(nc(c1)C)NS(=O)(=O)c1ccc(cc1)/N=C/c1ccc(cc1)N(=O)=O	CCCC/C=C\C(CCCCCCC(=O)O)C	OCC=C(CCC=C(CCC=C(C)C)C)C
Formula	C_20_H_13_N_5_O_2_	C_16_H_11_BrN_2_O	C_14_H_22_N_2_O_3_	C_19_H_17_N_5_O_4_S	C_15_H_28_O_2_	C_15_H_26_O
Molecular Weight	355.35	327.18	266.34	411.43	240.38	222.37
H-bond acceptors	4	3	4	7	2	1
H-bond donors	1	1	0	1	1	1
TPSA	85.57	45.48	49.85	138.51	37.30	20.23
iLOGP	2.64	2.58	3.01	2.64	3.45	3.71
ESOL Log S	−4.13	−4.7	−1.27	−3.77	−4.03	−4.17
ESOL Class	Moderately soluble	Moderately soluble	Very soluble	Soluble	Moderately soluble	Moderately soluble
GI absorption	High	High	High	Low	High	High
BBB permeant	No	Yes	No	No	Yes	Yes
Pgp substrate	No	No	No	No	No	No
CYP3A4 inhibitor	No	Yes	No	Yes	No	No
Lipinski violations	0	0	0	0	0	0
Bioavailability Score	0.55	0.55	0.55	0.55	0.85	0.55
PAINS alerts	0	0	0	0	0	0
Synthetic Accessibility	2.66	2.51	3.21	3.26	3.19	3.17
LD_50_ (mg/kg)	1500	2000	41	25,000	48	5000
Toxicity Class	IV	IV	II	VI	II	V
Hepatotoxicity	Active (0.55)	Active (0.58)	Inactive (0.93)	Inactive (0.57)	Active (0.51)	Inactive (0.79)
Neurotoxicity	Active (0.88)	Active (0.67)	Inactive (0.53)	Inactive (0.80)	Inactive (0.90)	Inactive (0.78)
Nephrotoxicity	Inactive (0.66)	Inactive (0.66)	Active (0.59)	Inactive (0.57)	Inactive (0.55)	Inactive (0.74)
Respiratory toxicity	Active (0.52)	Inactive (0.50)	Active (0.52)	Active (0.62)	Inactive (0.79)	Inactive (0.81)
Cardiotoxicity	Inactive (0.91)	Inactive (0.76)	Inactive (0.69)	Inactive (0.83)	Inactive (0.96)	Inactive (0.84)
Carcinogenicity	Active (0.62)	Inactive (0.60)	Inactive (0.50)	Active (0.68)	Inactive (0.66)	Inactive (0.76)
Immunotoxicity	Inactive (0.95)	Inactive (0.70)	Inactive (0.99)	Inactive (0.99)	Inactive (0.99)	Inactive (0.99)
Mutagenicity	Inactive (0.53)	Active (0.60)	Inactive (0.76)	Inactive (0.69)	Inactive (0.99)	Inactive (0.97)
Cytotoxicity	Inactive (0.86)	Inactive (0.61)	Inactive (0.75)	Inactive (0.83)	Inactive (0.72)	Inactive (0.85)

The table summarizes the physicochemical properties (molecular weight [MW], H-bond donors [HBD]/acceptors [HBA], topological polar surface area [TPSA]), drug-likeness indicators (Lipinski violations, bioavailability score [BS], pan-assay interference structures [PAINS] alerts, synthetic accessibility [SA], ADME parameters (Gastrointestinal [GI] absorption, blood–brain barrier [BBB] permeability, P-glycoprotein [Pgp] substrate, Cytochrome P450 3A4 [CYP3A4] inhibition), and toxicity endpoints (median lethal dose [LD_50_], hepatotoxicity, neurotoxicity, immunotoxicity, mutagenicity, cytotoxicity, etc.).

**Table 2:** Topical characteristics of *C. albicans* secondary metabolites in the MET–TAR network.

Secondary Metabolite	Degree Centrality	Betweenness Centrality	Closeness Centrality
MET 28	69	0.454	0.452
MET 34	65	0.427	0.444
MET 176	49	0.305	0.411
MET 181	42	0.229	0.399
MET 119	24	0.093	0.369
MET 15	14	0.040	0.355

The table shows the topological features of the six candidates *C. albicans* secondary metabolites within the metabolite–target (MET–TAR) network in terms of degree centrality (DC), betweenness centrality (BC), and closeness centrality (CC).

**Table 3:** The top 20 hub genes of the six candidate *Candida albicans* secondary metabolite targets involved in HIV latency.

Ensembl Name	Protein Name	Gene	Target Family	Degree	GIFTS	Relevance Score
ENSP00000451828	RAC-alpha serine/threonine-protein kinase	AKT1	Kinase	121	66	11.16784
ENSP00000398698	Tumor necrosis factor	TNF	Other	111	65	27.39799
ENSP00000275493	Epidermal growth factor receptor	EGFR	Kinase	99	68	8.50484
ENSP00000264657	Signal transducer and activator of transcription 3	STAT3	Transcription Factor	96	67	11.83733
ENSP00000263025	Mitogen-activated protein kinase 3	MAPK3	Kinase	80	62	11.06028
ENSP00000405330	Estrogen receptor	ESR1	Nuclear Receptor	80	67	4.92296
ENSP00000324806	Glycogen synthase kinase-3 beta	GSK3B	Kinase	79	64	6.64872
ENSP00000354558	Serine/threonine-protein kinase mTOR	MTOR	Kinase	79	68	5.14138
ENSP00000287820	Peroxisome proliferator-activated receptor gamma	PPARG	Nuclear Receptor	77	65	8.84661
ENSP00000356438	Prostaglandin G/H synthase 2	PTGS2	Enzyme	73	62	7.21239
ENSP00000361405	67 kDa matrix metalloproteinase-9	MMP9	Enzyme	69	66	8.05360
ENSP00000227507	G1/S-specific cyclin-D1	CCND1	Other	68	54	5.01859
ENSP00000269571	Receptor tyrosine-protein kinase erbB-2	ERBB2	Kinase	67	68	2.68417
ENSP00000258149	E3 ubiquitin-protein ligase Mdm2	MDM2	Enzyme	65	67	5.37644
ENSP00000371067	Tyrosine-protein kinase JAK2	JAK2	Kinase	63	66	2.80281
ENSP00000215832	Mitogen-activated protein kinase 1	MAPK1	Kinase	63	65	14.75959
ENSP00000263967	Phosphatidylinositol 4,5-bisphosphate 3-kinase catalytic subunit alpha isoform	PIK3CA	Kinase	62	65	7.64953
ENSP00000212015	NAD-dependent protein deacetylase sirtuin-1	SIRT1	Epigenetic	61	62	8.64687
ENSP00000384273	Transcription factor p65	RELA	Transcription Factor	59	64	15.96309
ENSP00000355759	Poly [ADP-ribose] polymerase 1	PARP1	Enzyme	58	64	8.59058

The table presents the hub nodes prioritized by degree centrality (DC) using CytoHubba v0.1. For each protein, the Ensembl ID, gene name, target family classification, DC score, GeneCards Inferred Functionality Score (GIFTS), and relevance score for HIV latency are provided.

**Table 4:** KEGG pathways potentially enriched by the six candidate *Candida albicans* secondary metabolites with established roles in HIV latency modulation from the literature.

KEGG ID	Pathway	Enrichment FDR	Fold Enrichment	No. of Pathway Genes	No. of Genes	Enriched Genes
hsa05200	Pathways in cancer	8.19 × 10^−47^	13.9028	530	57	RASGRP1, CDK2, CDK4, CREBBP, EDNRA, **EGFR**, **ERBB2**, **AKT1**, **ESR1**, FGFR1, FLT3, FLT4, **MTOR**, **GSK3B**, GSTP1, HDAC1, HDAC2, AR, JAK1, **JAK2**, JAK3, KIT, **MDM2**, MET, MMP1, MMP2, **MMP9**, NOS2, NTRK1, **PIK3CA**, PIK3CB, PIM1, PIK3CD, PPARD, **PPARG**, PRKCA, PRKCB, PRKCG, **MAPK1**, **MAPK3**, MAPK8, MAPK10, MAP2K1, **PTGS2**, PTK2, RAC1, **CCND1**, **RELA**, RET, SHH, BRAF, **STAT3**, TERT, VEGFA, CASP9, CCND2, and CCND3.
hsa04151	PI3K-Akt signaling pathway	1.45 × 10^−35^	15.3373	354	42	CDK2, CDK4, CHRM1, CHRM2, **EGFR**, **ERBB2**, **AKT1**, FGFR1, FLT1, FLT3, FLT4, **MTOR**, **GSK3B**, ITGA4, JAK1, **JAK2**, JAK3, KDR, KIT, MCL1, **MDM2**, MET, NOS3, NTRK1, PDPK1, **PIK3CA**, PIK3CB, PIK3CD, PIK3CG, PRKCA, **MAPK1**, **MAPK3**, MAP2K1, PTK2, RAC1, **CCND1**, **RELA**, SYK, VEGFA, CASP9, CCND2, and CCND3.
hsa05206	MicroRNAs in cancer	4.33 × 10^−31^	24.0878	161	30	CREBBP, DNMT1, **EGFR**, **ERBB2**, **SIRT1**, **MTOR**, HDAC1, HDAC2, MCL1, **MDM2**, MET, **MMP9**, **PIK3CA**, PIK3CB, PIM1, PIK3CD, PRKCA, PRKCB, PRKCE, PRKCG, **MAPK1**, **MAPK3**, MAP2K1, **PTGS2**, **CCND1**, **STAT3**, VEGFA, CCND2, HDAC4, and CDC25A.
hsa04510	Focal adhesion	5.50 × 10^−27^	18.7443	200	29	**EGFR**, **ERBB2**, **AKT1**, FLT1, FLT4, FYN, **GSK3B**, ITGA4, KDR, MET, PDPK1, **PIK3CA**, PIK3CB, PIK3CD, PRKCA, PRKCB, PRKCG, **MAPK1**, **MAPK3**, MAPK8, MAPK10, MAP2K1, PTK2, RAC1, **CCND1**, BRAF, VEGFA, CCND2, and CCND3.
hsa04014	Ras signaling pathway	6.46 × 10^−24^	15.4025	235	28	RASGRP1, **EGFR**, **AKT1**, FGFR1, FLT1, FLT3, FLT4, GRIN2B, KDR, KIT, MET, NTRK1, **PIK3CA**, PIK3CB, PIK3CD, PLA2G2A, PRKCA, PRKCB, PRKCG, **MAPK1**, **MAPK3**, MAPK8, MAPK10, MAP2K1, PTPN11, RAC1, **RELA**, and VEGFA.
hsa05235	PD-L1 expression and PD-1 checkpoint pathway in cancer	7.88 × 10^−23^	29.0497	89	20	RASGRP1, MAPK14, CSNK2A1, **EGFR**, **AKT1**, **MTOR**, JAK1, **JAK2**, LCK, **PIK3CA**, PIK3CB, PIK3CD, TLR9, PRKCQ, **MAPK1**, **MAPK3**, MAP2K1, PTPN11, **RELA**, and **STAT3**.
hsa04020	Calcium signaling pathway	1.77 × 10^−22^	14.5430	240	27	CHRM1, CHRM2, ADORA2A, DRD1, EDNRA, **EGFR**, **ERBB2**, FGFR1, FLT1, FLT4, GRM5, KDR, MET, NOS1, NOS2, NOS3, NTRK1, P2RX7, PRKCA, PRKCB, PRKCG, SPHK2, RET, TACR1, TRHR, VEGFA, and CACNA1B.
hsa04066	HIF-1 signaling pathway	4.18 × 10^−21^	23.7195	109	20	CREBBP, EGFR, **ERBB2**, **AKT1**, FLT1, **MTOR**, NOS2, NOS3, PIK3CA, PIK3CB, PIK3CD, PRKCA, PRKCB, PRKCG, **MAPK1**, **MAPK3**, MAP2K1, **RELA**, **STAT3**, and VEGFA.
hsa04071	Sphingolipid signaling pathway	2.37 × 10^−20^	21.7262	119	20	MAPK14, **AKT1**, FYN, NOS3, PDPK1, PIK3CA, PIK3CB, PIK3CD, PRKCA, PRKCB, PRKCE, PRKCG, **MAPK1**, **MAPK3**, MAPK8, MAPK10, MAP2K1, SPHK2, RAC1, and **RELA**.
hsa04024	cAMP signaling pathway	8.40 × 10^−20^	14.0385	221	24	CHRM1, CHRM2, ADORA2A, CREBBP, DRD1, DRD2, EDNRA, **AKT1**, GRIN2B, HTR1A, LIPE, PDE3B, PDE4A, PIK3CA, PIK3CB, PIK3CD, **MAPK1**, **MAPK3**, MAPK8, MAPK10, MAP2K1, RAC1, **RELA**, and BRAF.

The table shows the Kyoto Encyclopedia of Genes and Genomes identification (KEGG ID), enrichment false discovery rate (FDR), fold enrichment (FE), and number (No.) of pathway genes, No. of enriched genes with their Gene IDs of the overlapping genes of *C. albicans* secondary metabolites and HIV latency analysed using ShinyGO v0.82. Hub genes are shown in bold.

**Table 5:** BFE terms expressed in kcal/mol of MET 15 to hub targets MAPK1, PIK3CA, MAPK3, EGFR, MTOR, and AKT1 compared to their respective reference ligands.

MD System	BFE Terms (kcal/mol)						
	∆E_vdW_	∆E_elec_	∆G_GB_	∆G_SA_	∆G_gas_	∆G_solv_	∆G_bind_
MAPK1^MET 15^	−36.95 ± 0.11	−43.08 ± 0.19	51.27 ± 0.17	−4.90 ± 0.01	−80.03 ± 0.20	46.37 ± 0.16	−33.66 ± 0.16
MAPK1^VTX−11e^	−59.74 ± 0.12	−37.16 ± 0.32	48.20 ± 0.22	−7.38 ± 0.01	−96.90 ± 0.35	40.82 ± 0.21	−56.08 ± 0.18
PIK3CA^MET 15^	−50.54 ± 0.08	−18.61 ± 0.18	36.32 ± 0.12	−6.19 ± 0.01	−69.15 ± 0.20	30.12 ± 0.12	−39.03 ± 0.14
PIK3CA^RLY2608^	−77.60 ± 0.09	−33.09 ± 0.12	50.64 ± 0.10	−9.28 ± 0.01	−110.69 ± 0.14	41.36 ± 0.10	−69.32 ± 0.10
MAPK3^MET 15^	−36.18 ± 0.11	−14.40 ± 0.31	29.27 ± 0.25	−4.29 ± 0.01	−50.59 ± 0.36	24.98 ± 0.24	−25.61 ± 0.14
MAPK3^SCH772984^	−81.65 ± 0.12	−65.00 ± 0.23	67.96 ± 0.19	−9.65 ± 0.01	−146.65 ± 0.23	58.31 ± 0.19	−88.34 ± 0.14
EGFR^MET15^	−44.44 ± 0.08	−3.75 ± 0.14	22.14 ± 0.11	−5.39 ± 0.01	−48.19 ± 0.17	16.74 ± 0.11	−31.45 ± 0.10
EGFR^Erlotinib^	−52.01 ± 0.10	−21.40 ± 0.18	37.95 ± 0.15	−6.44 ± 0.01	−73.41 ± 0.22	31.51 ± 0.15	−41.90 ± 0.13
MTOR^MET 15^	−33.73 ± 0.18	−11.70 ± 0.20	28.31 ± 0.19	−3.89 ± 0.02	−45.43 ± 0.26	24.42 ± 0.19	−21.01 ± 0.16
MTOR^Torin2^	−44.73 ± 0.10	−28.16 ± 0.24	43.71 ± 0.18	−5.75 ± 0.01	−72.90 ± 0.24	37.96 ± 0.18	−34.94 ± 0.11
AKT1^MET 15^	−33.36 ± 0.11	−11.96 ± 0.39	29.60 ± 0.35	−4.00 ± 0.02	−45.32 ± 0.37	25.60 ± 0.34	−19.72 ± 0.11
AKT1^EX4^	−55.68 ± 0.12	−279.81 ± 0.55	291.48 ± 0.56	−7.42 ± 0.01	−335.48 ± 0.55	284.07 ± 0.56	−51.41 ± 0.13

The table shows the decomposition of the binding free energy (∆G_bind_) into the gas-phase energy (∆G_gas_), which comprises the electrostatic energy (∆E_elec_) and van der Waals energy (∆E_vdW_), and the solvation free energy (∆G_solv_), which comprises the polar solvation energy (∆G_GB_) and nonpolar solvation energy (∆G_SA_).

## Data Availability

The original contributions presented in this study are included in the article/Supplementary Materials. Further inquiries can be directed to the corresponding author.

## Supplementary Materials

The following supporting information can be downloaded at: https://www.mdpi.com/article/10.3390/ijms27073125/s1.
