# Supplementary material for: Network Pharmacology-Guided Identification of Candida albicans Secondary Metabolites as Modulators of HIV Latency via Oncogenic Signaling Pathways
Source: Int J Mol Sci. 2026 Mar 30;27(7):3125. doi: 10.3390/ijms27073125 (PMC13073563; doi:10.3390/ijms27073125)
Supplement: Supplementary file 1 [file ijms-27-03125-s001.zip › Supplementary Figures S1-S3.pdf]

Network Pharmacology-Guided Identification of *Candida albicans* Secondary Metabolites as Modulators of HIV Latency via Oncogenic Signaling Pathways

Ernest Oduro-Kwateng [1], Ugochukwu J. Anyaneji [1], Asiphe Fanele [1], Ntokozo Ntanzi [1], Mahmoud E. Soliman [2] and Nompumelelo P. Mkhwanazi [1,*]

[1]HIV Pathogenesis Programme, School of Laboratory Medicine and Medical Sciences, College of Health Science, University of KwaZulu-Natal, South Africa

[2]Molecular Bio-computation and Drug Design Research Group, School of Health Sciences, College of Health Science, University of KwaZulu-Natal, South Africa

*Corresponding author: Dr Nompumelelo P. Mkhwanazi

Email: mkhwanazi@ukzn.ac.za

University of KwaZulu-Natal

College of Health Science

School of Laboratory Medicine and Medical Science

HIV Pathogenesis Programme

DDMRI Building 2nd Floor Room 212

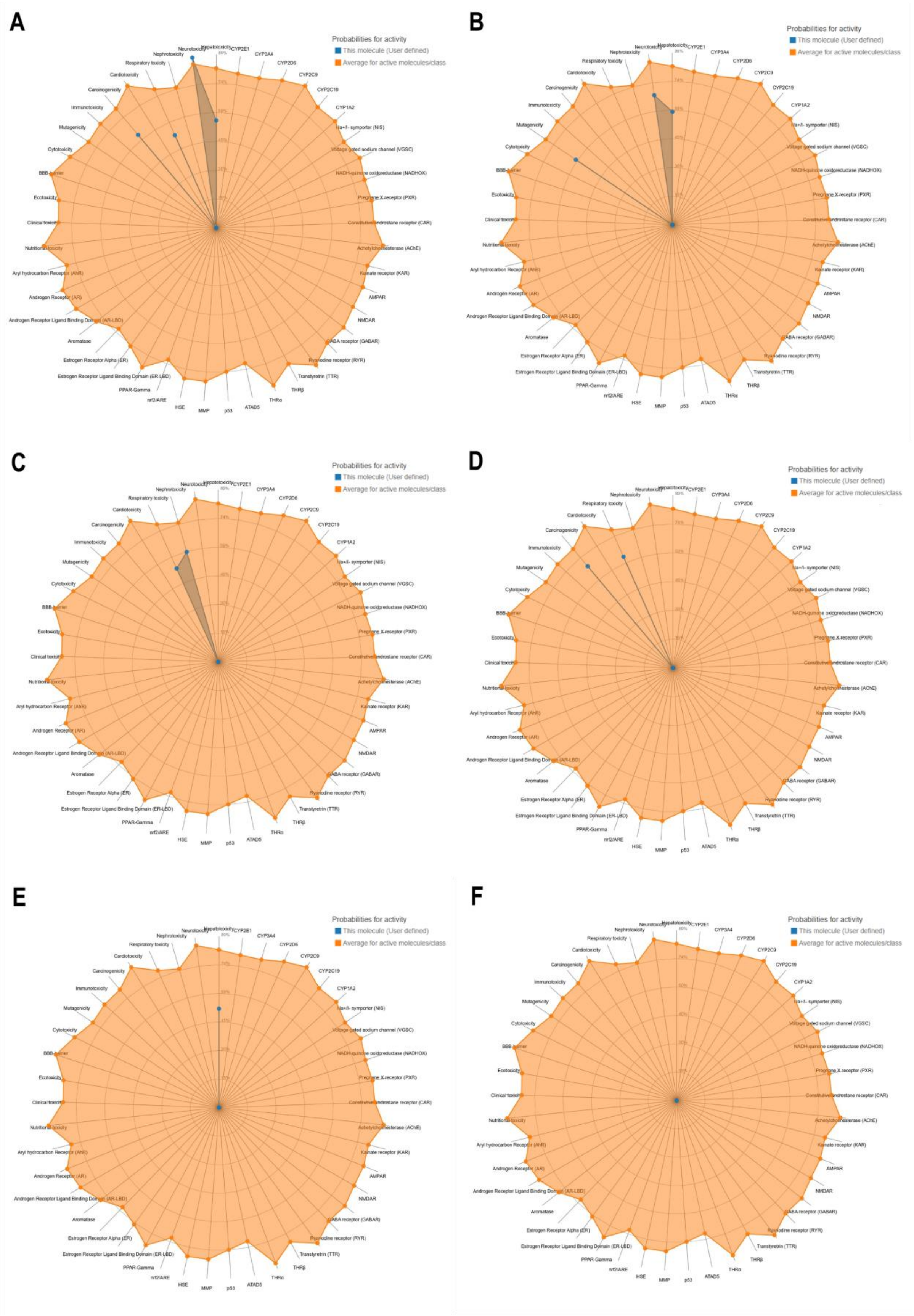

Supplementary Figure S1. Radar chart depicting the toxicity profiles of selected therapeutic metabolites, highlighting variations across multiple toxicity parameters.

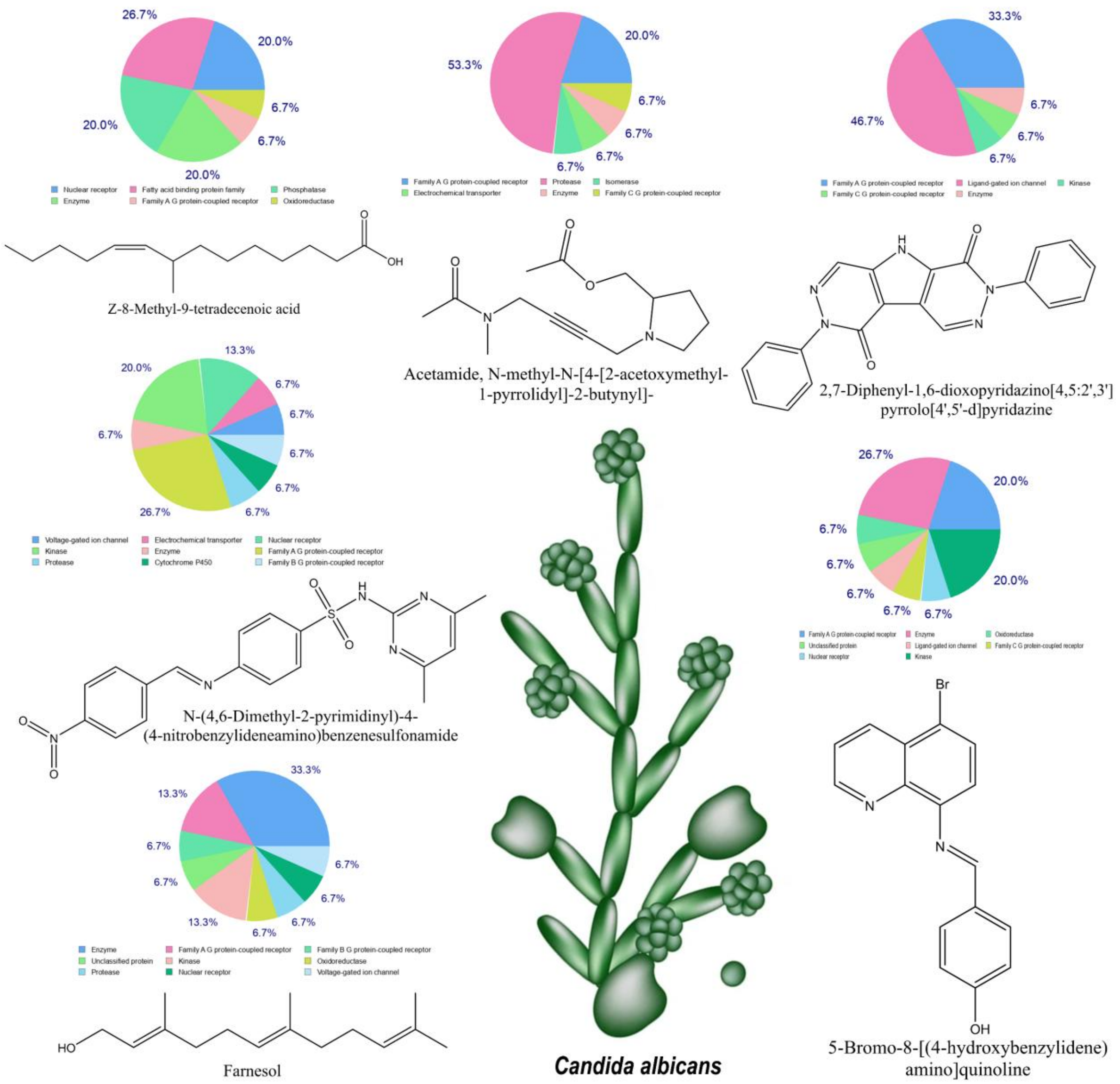

Supplementary Figure S2. 2D chemical structures and target class distribution of the six *Candida albicans* metabolites, 2,7-Diphenyl-1,6-dioxopyridazino[4,5:2',3']pyrrolo[4',5'-d]pyridazine (MET 15), 5-Bromo-8-[(4-hydroxybenzylidene)amino]quinoline (MET 28), acetamide, N-methyl-N-[4-[2-acetoxymethyl-1-pyrrolidyl]-2-butynyl]- (MET 34), N-(4,6-Dimethyl-2-pyrimidinyl)-4-(4-nitrobenzylideneamino)benzenesulfonamide (MET 119), Z-8-Methyl-9 tetradecenoic acid (MET 176), and farnesol (MET 181). Pie charts represent the percentage distribution of predicted human protein targets by class, as identified using SwissTargetPrediction. The target classes include enzymes, kinases, nuclear receptors, G-protein-coupled receptors (GPCRs), transporters, and ion channels. Variability in the target class distribution suggests a differential polypharmacological potential for the metabolites. Centered is a stylized depiction of *C. albicans* in its filamentous hyphal/pseudohyphal form, which is associated with its invasive and pathogenic phenotype.

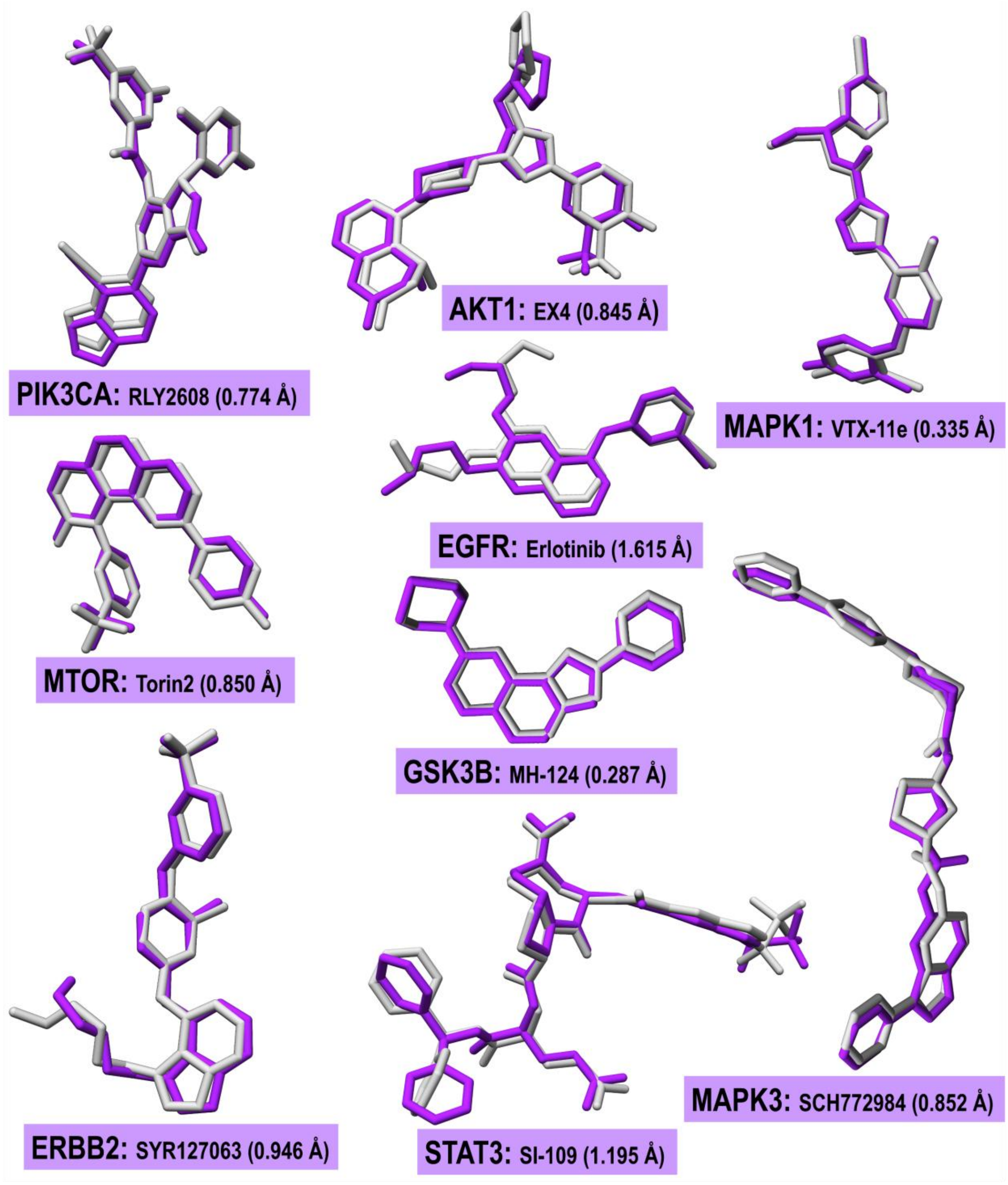

Supplementary Figure S3. Molecular docking protocol validation by redocking of co-crystallized ligands in hub protein binding sites. Superimposition of co-crystallized ligands (purple sticks) and their redocked conformations (grey sticks) demonstrates accurate reproduction of experimental binding poses (RMSD < 2.0 Å).
