# Supplementary material for: Network Pharmacology-Guided Identification of Candida albicans Secondary Metabolites as Modulators of HIV Latency via Oncogenic Signaling Pathways
Source: Int J Mol Sci. 2026 Mar 30;27(7):3125. doi: 10.3390/ijms27073125 (PMC13073563; doi:10.3390/ijms27073125)
Supplement: Supplementary file 1 [file ijms-27-03125-s001.zip › Supplementary Tables S1-S8.pdf]

Network Pharmacology-Guided Identification of *Candida albicans* Secondary Metabolites as Modulators of HIV Latency via Oncogenic Signaling Pathways

Ernest Oduro-Kwateng [1], Ugochukwu J. Anyaneji [1], Asiphe Fanele [1], Ntokozo Ntanzi [1], Mahmoud E. Soliman [2] and Nompumelelo P. Mkhwanazi [1,*]

[1]HIV Pathogenesis Programme, School of Laboratory Medicine and Medical Sciences, College of Health Science, University of KwaZulu-Natal, South Africa

[2]Molecular Bio-computation and Drug Design Research Group, School of Health Sciences, College of Health Science, University of KwaZulu-Natal, South Africa

*Corresponding author: Dr Nompumelelo P. Mkhwanazi

Email: mkhwanazi@ukzn.ac.za

University of KwaZulu-Natal

College of Health Science

School of Laboratory Medicine and Medical Science

HIV Pathogenesis Programme

DDMRI Building 2nd Floor Room 212

Supplementary Table S1. Druglikeness and ADME profiles of reported *Candida albicans* secondary metabolites using SwissADME webserver.

Secondary Metabolite	Name	Pubchem CID	Canonical SMILES	MF	MW	HBA	HBD	TPSA	iLOGP	ESOL Log S	ESOL Class	GI	BBB	Pgp	CYP3A 4	RO5	B.S	PAINS alerts	SA
MET 1	1-(4-Amino-1,2,5-oxadiazol-3-YL)-5-[(dimethyl-amino)methyl]-1H-1,2,3-triazole-4-carboxylic acid	546918	CN(Cc1c(nnn1c1nonc1N)C(=O)O)C	C8H11N7O3	253.22	8	2	136.19	1.54	0.58	Highly soluble	Low	No	No	No	0	0.55	0	3.1
MET 2	Methyl ethyl phthalate	36656	CCOC(=O)c1ccccc1C(=O)OC	C11H12O4	208.21	4	0	52.6	2.08	-2.39	Soluble	High	Yes	No	No	0	0.55	0	1.83
MET 3	Dihydroxyacetone	670	OCC(=O)CO	C3H6O3	90.08	3	2	57.53	0.48	0.59	Highly soluble	High	No	No	No	0	0.55	0	1
MET 4	2,5-Di-tert-butylhydroquinone	2374	CC(c1cc(O)c(cc1O)C(C)(C)C)(C)C	C14H22O2	222.32	2	2	40.46	2.91	-4.23	Moderately soluble	High	Yes	No	No	0	0.55	0	1.46
MET 5	10-Heptadecen-8-ynoic acid, methyl ester, (E)-	5367407	CCCCCC/C=C/C#CCCCCCCC(=O)OC	C18H30O2	278.43	2	0	26.3	4.72	-4.69	Moderately soluble	High	Yes	No	No	1	0.55	0	3.88
MET 6	11-Dodecanolide	543499	CC1CCCCCCCCCC(=O)O1	C12H22O2	198.3	2	0	26.3	2.8	-3.61	Soluble	High	Yes	No	No	0	0.55	0	2.99
MET 7	17-Octadecynoic acid	1449	C#CCCCCCCCCCCCCCCCC(=O)O	C18H32O2	280.45	2	1	37.3	3.98	-5.13	Moderately soluble	High	Yes	No	No	1	0.85	0	3.35
MET 8	1-Docosene	74138	CCCCCCCCCCCCCCCCCCCCC=C	C22H44	308.58	0	0	0	6.02	-8.19	Poorly soluble	Low	No	Yes	No	1	0.55	0	3.28
MET 9	1-Methyl-8-propyl-3,6-diazahomoadamantan-9-ol	551662	CCCC12CN3CCN(C1)CC(C2O)(C3)C	C13H24N2O	224.34	3	1	26.71	2.52	-1.86	Very soluble	High	No	No	No	0	0.55	0	4.44
MET 10	3-Butyldihydrofuran-2(3H)-one	86852	CCCCC1CCOC1=O	C8H14O2	142.2	2	0	26.3	2.09	-1.9	Very soluble	High	Yes	No	No	0	0.55	0	2.19
MET 11	Gamma-undecalactone	134970660	CCCCCCCC1CCC(=O)O1	C11H20O2	184.28	2	0	26.3	2.83	-2.64	Soluble	High	Yes	No	No	0	0.55	0	2.56
MET 12	2,3-Butanediol	262	CC(C(O)C)O	C4H10O2	90.12	2	2	40.46	1.26	0.25	Highly soluble	High	No	No	No	0	0.55	0	1.48
MET 13	2,3-Diaminopropionic acid	364	NC(C(=O)O)CN	C3H8N2O2	104.11	4	3	89.34	-0.13	2.36	Highly soluble	High	No	No	No	0	0.55	0	1.32
MET 14	2,5,5,8a-Tetramethyl-6,7,8,8a-tetrahydro-5H-chromen-8-ol	584153	CC1=CC=C2C(O1)(C)C(O)CCC2(C)C	C13H20O2	208.3	2	1	29.46	2.54	-2.57	Soluble	High	Yes	No	No	0	0.55	0	4.75

MET 15	2,7-Diphenyl-1,6-dioxopyridazino[4,5:2',3']pyrrolo[4',5'-d]pyridazine	135833395	O=c1n(ncc2c1[nH]c1c2c(=O)n(nc1)c1ccccc1)c1ccccc1	C20H13N5O2	355.35	4	1	85.57	2.64	-4.13	Moderately soluble	High	No	No	No	0	0.55	0	2.66
MET 16	2-Aminobutyrate	517460	[O-]C(=O)C([NH3+])CC	C4H9NO2	103.12	2	1	67.77	0.95	1.25	Highly soluble	Low	No	No	No	0	0.55	0	1.1
MET 17	D-2-Deoxyribose	5460005	O[C@H]([C@@H](CO)O)CC=O	C5H10O4	134.13	4	3	77.76	0.66	1.02	Highly soluble	High	No	No	No	0	0.55	0	2.36
MET 18	2-Carbamyl-9-[beta-d-ribofuranosyl]hypoxanthine	14191026	OC[C@H]1O[C@H]([C@@H]([C@@H]1O)O)n1cnc2c1nc(nc2)C(=O)N	C11H13N5O5	295.25	8	4	156.61	0.83	-0.67	Very soluble	Low	No	No	No	0	0.55	0	3.88
MET 19	2-Octanol, 2-methyl-6-methylene-, 2-formate	175155	O=COC(CCCC(=C)CC)(C)C	C11H20O2	184.28	2	0	26.3	2.81	-2.8	Soluble	High	Yes	No	No	0	0.55	0	1.93
MET 20	Alpha-Ketoglutaric Acid	51	OC(=O)CCC(=O)C(=O)O	C5H6O5	146.1	5	2	91.67	-0.09	0.09	Highly soluble	High	No	No	No	0	0.56	0	1.61
MET 21	Ketoleucine	70	CC(CC(=O)C(=O)O)C	C6H10O3	130.14	3	1	54.37	1.06	-1.04	Very soluble	High	Yes	No	No	0	0.85	0	1.04
MET 22	3-Buten-1-ol	69389	OCCC=C	C4H8O	72.11	1	1	20.23	1.47	-0.56	Very soluble	High	No	No	No	0	0.55	0	1.51
MET 23	3-Hydroxykynurenine	89	OC(=O)C(CC(=O)c1cccc(c1N)O)N	C10H12N2O4	224.21	5	4	126.64	0.59	0.36	Highly soluble	High	No	No	No	0	0.55	0	2.06
MET 24	3-Methyl-2-oxovaleric acid	47	CC(C(=O)C(=O)O)CC	C6H10O3	130.14	3	1	54.37	0.93	-1.12	Very soluble	High	Yes	No	No	0	0.85	0	1.49
MET 25	Gamma-Aminobutyric Acid	119	NCCCC(=O)O	C4H9NO2	103.12	3	2	63.32	0.72	1.72	Highly soluble	High	No	No	No	0	0.55	0	1
MET 26	4-Hydroxycyclohexane carboxylic acid	151138	OC1CCC(CC1)C(=O)O	C7H12O3	144.17	3	2	57.53	0.63	-0.88	Very soluble	High	No	No	No	0	0.85	0	2.41
MET 27	5,7-Dodecadiyne-1,12-diol	560878	OCCCCC#CC#CCCCCO	C12H18O2	194.27	2	2	40.46	2.84	-1.69	Very soluble	High	Yes	No	No	0	0.55	0	3.42
MET 28	5-Bromo-8-[(4-hydroxybenzylidene)amino]quinoline	582017	Oc1ccc(cc1)C=Nc1ccc(c2c1nccc2)Br	C16H11BrN2O	327.18	3	1	45.48	2.58	-4.7	Moderately soluble	High	Yes	No	Yes	0	0.55	0	2.51
MET 29	5-Hydroxykynurenine	440745	OC(=O)C(CC(=O)c1cc(O)ccc1N)N	C10H12N2O4	224.21	5	4	126.64	0.17	0.36	Highly soluble	High	No	No	No	0	0.55	0	2.06
MET 30	5'-Methylthioadenosine	439176	CSC[C@H]1O[C@H]([C@@H]([C@@H]1O)O)n1cnc2c1ncnc2N	C11H15N5O3S	297.33	6	3	144.61	1.27	-1.65	Very soluble	Low	No	No	No	0	0.55	0	4.12
MET 31	Petroselaidic acid	5282754	CCCCCCCCCCC/C=C/CCCCC(=O)O	C18H34O2	282.46	2	1	37.3	4.25	-5.41	Moderately soluble	High	No	No	No	1	0.85	0	3.07

MET 32	24,25-Dihydroxycholecalciferol	6434253	O[C@H]1CCC(=C)/C(=C\C=C\2/CCC[C@]3([C@H]2CC[C@@H]3[C@@H](CCC(C(O)(C)C)O)C)C)/C1	C27H44O3	416.64	3	3	60.69	4.25	-5.41	Moderately soluble	High	No	No	No	1	0.85	0	3.07
MET 33	9,12,15-Octadecatrienoic acid, 2-[(trimethylsilyl)oxy]-1-[[(trimethylsilyl)oxy]methyl]ethyl ester, (Z,Z,Z)-	5362857	CC/C=C/C/C=C/C/C=C/CCCCCCCC(=O)OC(CO[Si](C)(C)C)CO[Si](C)(C)C	C27H52O4Si2	496.87	4	0	44.76	7.39	-7.1	Poorly soluble	Low	No	No	Yes	1	0.55	0	5.4
MET 34	Acetamide, N-methyl-N-[4-[2-acetoxymethyl-1-pyrrolidyl]-2-butynyl]-	580233	CC(=O)OCC1CCCN1CC#CCN(C(=O)C)C	C14H22N2O3	266.34	4	0	49.85	3.01	-1.27	Very soluble	High	No	No	No	0	0.55	0	3.21
MET 35	Acetamide, N-methyl-N-[4-(3-hydroxypyrrolidinyl)-2-butynyl]-	536669	OC1CCN(C1)CC#CCN(C(=O)C)C	C11H18N2O2	210.27	3	1	43.78	2.26	-0.52	Very soluble	High	No	No	No	0	0.55	0	3.04
MET 36	Acetate	175	[O-]C(=O)C	C2H3O2-	59.04	2	0	40.13	0.63	-0.07	Very soluble	Low	No	No	No	0	0.85	0	1
MET 37	Cyclohexyl acetate	12146	CC(=O)OC1CCCCC1	C8H14O2	142.2	2	0	26.3	2.02	-2.71	Soluble	High	Yes	No	No	0	0.55	0	1.55
MET 38	Acetoin	179	CC(=O)C(O)C	C4H8O2	88.11	2	1	37.3	0.98	-0.13	Very soluble	High	No	No	No	0	0.55	0	1
MET 39	Acetone	180	CC(=O)C	C3H6O	58.08	1	0	17.07	1.14	-0.17	Very soluble	High	No	No	No	0	0.55	0	1
MET 40	Adenine	190	Nc1ncnc2c1[nH]cn2	C5H5N5	135.13	3	2	80.48	-0.11	-1.29	Very soluble	High	No	No	No	0	0.55	0	1.67
MET 41	Adenosine	60961	OC[C@H]1O[C@H]([C@@H]([C@@H]1O)O)n1cnc2c1ncnc2N	C10H13N5O4	267.24	7	4	139.54	0.61	-1.05	Very soluble	Low	No	No	No	0	0.55	0	3.86
MET 42	Adenosine-5'-diphosphate	6022	O[C@@H]1[C@@H](COP(=O)(OP(=O)(O)O)O)O[C@H]([C@@H]1O)n1cnc2c1ncnc2N	C10H15N5O10P2	427.2	13	6	252.22	-0.53	0.57	Highly soluble	Low	No	No	No	2	0.11	0	4.74
MET 43	Alanine	5950	C[C@@H](C(=O)O)N	C3H7NO2	89.09	3	2	63.32	0.34	1.54	Highly soluble	High	No	No	No	0	0.55	0	1
MET 44	Alloisoleucine, DL-	99288	CC[C@H]([C@@H](C(=O)O)N)C	C6H13NO2	131.17	3	2	63.32	0.86	0.63	Highly soluble	High	No	No	No	0	0.55	0	1.65

MET 45	Adenosine Phosphate	6083	O[C@@H]1[C@H](O)[C@H](O[C@H]1n1cnc2c1ncnc2N)COP(=O)(O)O	C10H14N5O7P	347.22	10	5	195.88	-0.1	0.2	Highly soluble	Low	No	No	No	1	0.11	0	4.35
MET 46	Arabitol	94154	OC[C@H](C([C@@H](CO)O)O)O	C5H12O5	152.15	5	5	101.15	1.18	1.04	Highly soluble	Low	No	No	No	0	0.55	0	2.94
MET 47	Asparagine	6267	NC(=O)C[C@@H](C(=O)O)N	C4H8N2O3	132.12	4	3	106.41	-0.26	1.69	Highly soluble	High	No	No	No	0	0.55	0	1.72
MET 48	Aspartic Acid	5960	OC(=O)C[C@@H](C(=O)O)N	C4H7NO4	133.1	5	3	100.62	-0.09	1.98	Highly soluble	High	No	No	No	0	0.56	0	1.8
MET 49	N,N-Dimethylarginine	123831	OC(=O)[C@H](CCCN=C(N(C)C)N)N	C8H18N4O2	202.25	4	3	104.94	1.17	1.58	Highly soluble	High	No	No	No	0	0.55	0	2.92
MET 50	Benzene, (1-ethyl-1-propenyl)-	5370623	CC/C(=C\C)/c1ccccc1	C11H14	146.23	0	0	0	2.62	-3.53	Soluble	Low	Yes	No	No	1	0.55	0	1.67
MET 51	2-Ethyl-p-xylene	15653	CCc1cc(C)ccc1C	C10H14	134.22	0	0	0	2.5	-3.84	Soluble	Low	Yes	No	No	1	0.55	0	1
MET 52	Pentamethylbenzene	12784	Cc1cc(C)c(c(c1C)C)C	C11H16	148.24	0	0	0	2.6	-4.04	Moderately soluble	Low	Yes	No	No	1	0.55	0	1
MET 53	Durene	7269	Cc1cc(C)c(cc1C)C	C10H14	134.22	0	0	0	2.47	-3.64	Soluble	Low	Yes	No	No	1	0.55	0	1
MET 54	m-Xylene	7929	Cc1cccc(c1)C	C8H10	106.17	0	0	0	2.12	-3.07	Soluble	Low	Yes	No	No	0	0.55	0	1
MET 55	Benzyl Benzoate	2345	O=C(c1ccccc1)OCc1ccccc1	C14H12O2	212.24	2	0	26.3	2.68	-3.95	Soluble	High	Yes	No	No	0	0.55	0	1.44
MET 56	Bis(2-ethylhexyl) phthalate	8343	CCCCC(COC(=O)c1ccccc1C(=O)OCC(CCCC)CC)CC	C24H38O4	390.56	4	0	52.6	4.77	-6.06	Poorly soluble	High	No	Yes	Yes	1	0.55	0	4.12
MET 57	Dimethyl benzyl carbinyl butyrate	24915	CCCC(=O)OC(Cc1ccccc1)(C)C	C14H20O2	220.31	2	0	26.3	3.09	-3.31	Soluble	High	Yes	No	No	0	0.55	0	1.92
MET 58	Carbamic acid, N-methyl-, (6-chloro-2-methyl-1,1-dioxidobenzo[b]thioxan-4-yliden)amino ester	9603472	CNC(=O)O/N=C/1\CC(C)S(=O)(=O)c2c1cc(Cl)cc2	C12H13ClN2O4S	316.76	5	1	93.21	2.31	-3.02	Soluble	High	No	No	No	0	0.55	0	3.61
MET 59	N-(tetradecanoyl)-sphinganine	10255824	CCCCCCCCCCCCCCC[C@H]([C@@H](NC(=O)CCCCCCCCCCCCC)CO)O	C32H65NO3	511.86	3	3	69.56	7	-8.78	Poorly soluble	Low	No	No	No	2	0.17	0	5.1
MET 60	Choline	305	OCC[N+](C)(C)C	C5H14NO+	104.17	1	1	20.23	-2.14	-0.1	Very soluble	Low	No	No	No	0	0.55	0	1

MET 61	Chromone, 5-hydroxy-6,7,8-trimethoxy-2,3-dimethyl-	5377709	COc1c(OC)c(O)c2c(c1OC)oc(c(c2=O)C)C	C14H16O6	280.27	6	1	78.13	3.1	-3.26	Soluble	High	No	No	No	0	0.55	0	3.33
MET 62	Citrate anion	31348	[O-]C(=O)C(CC(=O)[O-])(CC(=O)[O-])O	C6H5O7---	189.1	7	1	140.62	-0.81	0.4	Highly soluble	Low	No	No	No	0	0.56	0	2.09
MET 63	Creatinine	588	O=C1N=C(N(C1)C)N	C4H7N3O	113.12	2	1	58.69	0.46	0.57	Highly soluble	Low	No	No	No	0	0.55	0	2.74
MET 64	Curan-17-oic acid, 19,20-dihydroxy-, methyl ester, (19S)-	546481	COC(=O)C1C2CC3C4(C1Nc1c4cccc1)CCN3CC2(O)C(O)C	C20H26N2O4	358.43	5	3	82.03	2.7	-2.67	Soluble	High	No	Yes	No	0	0.55	0	5.35
MET 65	4-tert-Butylcyclohexyl acetate	36081	CC(=O)OC1CCC(CC1)C(C)(C)C	C12H22O2	198.3	2	0	26.3	2.7	-3.9	Soluble	High	Yes	No	No	0	0.55	0	2.73
MET 66	Cytidine	6175	OC[C@H]1O[C@H]([C@@H]([C@@H]1O)O)n1ccc(nc1=O)N	C9H13N3O5	243.22	6	4	130.83	0.44	-0.14	Very soluble	Low	No	No	No	0	0.55	0	3.84
MET 67	Desulphosinigrin	9601716	C=CC/C(=N\O)/SC1OC(CO)C(C(C1O)O)O	C10H17NO6S	279.31	7	5	148.04	0.68	-0.44	Very soluble	Low	No	Yes	No	0	0.55	0	5.14
MET 68	6-O-Alpha-D-Galactopyranosyl-Alpha-D-Glucopyranose	6602503	OC[C@H]1O[C@H](OC[C@H]2O[C@H](O)[C@@H]([C@H]([C@@H]2O)O)O)[C@@H]([C@H]([C@H]1O)O)O	C12H22O11	342.3	11	8	189.53	-0.91	1.66	Highly soluble	Low	No	Yes	No	2	0.17	0	5.41
MET 69	D-glycero-L-gluco-Heptose	21139463	OC[C@H]([C@@H]([C@@H]([C@H]([C@@H](C=O)O)O)O)O)O	C7H14O7	210.18	7	6	138.45	-0.67	1.5	Highly soluble	Low	No	No	No	1	0.55	0	3.6
MET 70	Diethyl Phthalate	6781	CCOC(=O)c1ccccc1C(=O)OCC	C12H14O4	222.24	4	0	52.6	2.26	-2.62	Soluble	High	Yes	No	No	0	0.55	0	1.93
MET 71	D-Mannose	18950	OC[C@H]1OC(O)[C@H]([C@H]([C@@H]1O)O)O	C6H12O6	180.16	6	5	110.38	0.31	1.15	Highly soluble	Low	No	Yes	No	0	0.55	0	4.08
MET 72	Edulan II	6432428	C[C@H]1CC=C2[C@](O1)(C)C=CCC2(C)C	C13H20O	192.3	1	0	9.23	3.03	-2.9	Soluble	High	Yes	No	No	0	0.55	0	4.21
MET 73	Ergosta-5,22-dien-3 ol,acetate,(3β,22 E)-	13889457	CC(=O)O[C@H]1CC[C@]2(C(=CC[C@@H]3[C@@H]2CC[C@]2([C@	C30H48O2	440.7	2	0	26.3	5.18	-7.59	Poorly soluble	Low	No	No	No	1	0.55	0	6.12

			H]3CC[C@@H]2[C@@H](/C=C/[C@@H](C(C)C)C)C)C)C1)C																
MET 74	Erythritol	222285	OC[C@H]([C@H](CO)O)O	C4H10O4	122.12	4	4	80.92	0.94	1.04	Highly soluble	Low	No	No	No	0	0.55	0	2.42
MET 75	Estra-1,3,5(10)-trien-17-ol	537293	OC1CCC2C1(C)CCC1C2CCc2c1cccc2	C18H24O	256.38	1	1	20.23	2.94	-4.42	Moderately soluble	High	Yes	Yes	No	1	0.55	0	3.37
MET 76	Ethanol	702	CCO	C2H6O	46.07	1	1	20.23	1.1	-0.07	Very soluble	Low	No	No	No	0	0.55	0	1
MET 77	Ethyl acetoacetate ethyleneglycol ketal	80865	CCOC(=O)CC1(C)OCCO1	C8H14O4	174.19	4	0	44.76	2.39	-0.81	Very soluble	High	Yes	No	No	0	0.55	0	2.27
MET 78	Formate	283	[O-]C=O	CHO2-	45.02	2	0	40.13	0.26	0.01	Highly soluble	Low	No	No	No	0	0.85	0	1
MET 79	Fumarate	5460307	[O-]C(=O)/C=C/C(=O)[O-]	C4H2O4--	114.06	4	0	80.26	0.32	-0.2	Very soluble	Low	No	No	No	0	0.56	0	1.75
MET 80	D-Glucose	5793	OC[C@H]1OC(O)[C@@H]([C@H]([C@@H]1O)O)O	C6H12O6	180.16	6	5	110.38	0.35	1.15	Highly soluble	Low	No	Yes	No	0	0.55	0	4.08
MET 81	alpha-D-glucose 1-phosphate	65533	OC[C@H]1O[C@H](OP(=O)(O)O)[C@@H]([C@H]([C@@H]1O)O)O	C6H13O9P	260.14	9	6	166.72	-0.19	1.44	Highly soluble	Low	No	Yes	No	1	0.11	0	4.82
MET 82	Glutamine	5961	NC(=O)CC[C@@H](C(=O)O)N	C5H10N2O3	146.14	4	3	106.41	0.38	1.5	Highly soluble	High	No	No	No	0	0.55	0	1.76
MET 83	Glutathione	124886	SC[C@@H](C(=O)NCC(=O)O)NC(=O)CC[C@@H](C(=O)O)N	C10H17N3O6S	307.32	7	5	197.62	0.32	1.82	Highly soluble	Low	No	No	No	0	0.11	0	3.06
MET 84	Glycerin	753	OCC(CO)O	C3H8O3	92.09	3	3	60.69	0.45	0.83	Highly soluble	High	No	No	No	0	0.55	0	1.31
MET 85	sn-Glycerol 3-phosphate	439162	OC[C@H](COP(=O)(O)O)O	C3H9O6P	172.07	6	4	117.03	-0.45	1.16	Highly soluble	High	No	No	No	0	0.56	0	3.95
MET 86	Choline Alfoscerate	657272	OC[C@H](COP(=O)(OCC[N+](C)(C)C)[O-])O	C8H20NO6P	257.22	6	2	108.86	-3.05	0.52	Highly soluble	High	No	No	No	0	0.55	0	4.38
MET 87	Glycine	750	NCC(=O)O	C2H5NO2	75.07	3	2	63.32	0.37	1.78	Highly soluble	High	No	No	No	0	0.55	0	1
MET 88	Guanosine	135398635	OC[C@H]1O[C@H]([C@@H]([C@@H]1O)O)n1cnc2c1nc(N)[nH]c2=O	C10H13N5O5	283.24	7	5	159.51	-0.22	-0.61	Very soluble	Low	No	No	No	0	0.55	0	3.86

MET 89	Histidine	6274	N[C@H](C(=O)O)Cc1cnc[nH]1	C6H9N3O2	155.15	4	3	92	-0.03	1.09	Highly soluble	High	No	No	No	0	0.55	0	2.29
MET 90	L-Homocitrulline	65072	NC(=O)NCCCC[C@@H](C(=O)O)N	C7H15N3O3	189.21	4	4	118.44	0.67	1.23	Highly soluble	High	No	No	No	0	0.55	0	1.93
MET 91	Homogentisic Acid	780	OC(=O)Cc1cc(O)ccc1O	C8H8O4	168.15	4	3	77.76	0.6	-1.66	Very soluble	High	No	No	No	0	0.56	0	1.41
MET 92	Hydroxyacetone	8299	CC(=O)CO	C3H6O2	74.08	2	1	37.3	0.9	0.21	Highly soluble	High	No	No	No	0	0.55	0	1
MET 93	Hypoxanthine	135398638	O=c1[nH]cnc2c1[nH]cn2	C5H4N4O	136.11	3	2	74.43	-0.17	-1.04	Very soluble	High	No	No	No	0	0.55	0	1.59
MET 94	Inosine	135398641	OC[C@H]1O[C@H]([C@@H]([C@@H]1O)O)n1cnc2c1nc[nH]c2=O	C10H12N4O5	268.23	7	4	133.49	0.25	-0.9	Very soluble	Low	No	No	No	0	0.55	0	3.77
MET 95	Inositol	892	OC1C(O)C(O)C(C(C1O)O)O	C6H12O6	180.16	6	6	121.38	0.31	1.38	Highly soluble	Low	No	Yes	No	1	0.55	0	3.53
MET 96	Isopropyl 9Z-tetradecenoate	56936038	CCCC/C=C\CCCCCCCC(=O)OC(C)C	C17H32O2	268.43	2	0	26.3	4.49	-4.57	Moderately soluble	High	Yes	No	No	1	0.55	0	2.99
MET 97	Isobutyrate	165337	CC(C(=O)[O-])C	C4H7O2-	87.1	2	0	40.13	1.11	-0.91	Very soluble	High	No	No	No	0	0.85	0	1
MET 98	l-Isoleucine	6306	CC[C@@H]([C@@H](C(=O)O)N)C	C6H13NO2	131.17	3	2	63.32	1.29	0.63	Highly soluble	High	No	No	No	0	0.55	0	1.65
MET 99	Lactate	91435	[O-]C(=O)C(O)C	C3H5O3-	89.07	3	1	60.36	0.75	0.13	Highly soluble	Low	No	No	No	0	0.85	0	1.27
MET 100	L-Aspartate 4-semialdehyde	5461143	O=CC[C@@H](C(=O)[O-])N	C4H6NO3-	116.1	4	1	83.22	0.28	2.11	Highly soluble	Low	No	No	No	0	0.55	0	1.46
MET 101	Leucine	6106	N[C@H](C(=O)O)CC(C)C	C6H13NO2	131.17	3	2	63.32	1.15	0.5	Highly soluble	High	No	No	No	0	0.55	0	1.39
MET 102	3,4-Dihydroxy-5-(1,2,3,4-tetrahydroxybutyl)oxolan-2-one	219893	OCC(C(C(C1OC(=O)C(C1O)O)O)O)O	C8H14O8	238.19	8	6	147.68	0.51	1.01	Highly soluble	Low	No	Yes	No	1	0.55	0	3.94
MET 103	Linoleic Acid	5280450	CCCCC/C=C\C/C=C\CCCCCCCC(=O)O	C18H32O2	280.45	2	1	37.3	0.51	1.01	Highly soluble	Low	No	Yes	No	1	0.55	0	3.94
MET 104	Lysine	5962	NCCCC[C@@H](C(=O)O)N	C6H14N2O2	146.19	4	3	89.34	0.97	1.51	Highly soluble	High	No	No	No	0	0.55	0	1.75
MET 105	1-Palmitoyl-sn-glycero-3-phosphocholine	460602	CCCCCCCCCCCCCCCC(=O)OC[C@H](COP(=O)(OCC[N+](C)(C)C)[O-])O	C24H50NO7P	495.63	7	1	114.93	0.4	-4.88	Moderately soluble	Low	No	Yes	Yes	0	0.55	0	5.75

MET 106	1-(9Z-hexadecenoyl)-sn-glycero-3-phosphocholine	24779461	CCCCCC/C=C\CCCCCCCC(=O)OC[C@H](COP(=O)(OCC[N+](C)(C)C)[O-])O	$C_{24}H_{48}NO_7P$	493.61	7	1	114.93	0.56	-4.34	Moderately soluble	Low	No	Yes	Yes	0	0.55	0	5.69
MET 107	1-Stearoyl-sn-glycero-3-phosphocholine	497299	CCCCCCCCCCCCCCCCCC(=O)OC[C@H](COP(=O)(OCC[N+](C)(C)C)[O-])O	$C_{26}H_{54}NO_7P$	523.68	7	1	114.93	0.92	-5.6	Moderately soluble	Low	No	Yes	Yes	1	0.55	0	6
MET 108	1-(9Z-octadecenoyl)-sn-glycero-3-phosphocholine	16081932	CCCCCCCC/C=C\CCCCCCCC(=O)OC[C@H](COP(=O)(OCC[N+](C)(C)C)[O-])O	$C_{26}H_{52}NO_7P$	521.67	7	1	114.93	0.49	-5.07	Moderately soluble	Low	No	Yes	Yes	1	0.55	0	5.94
MET 109	1-(9Z,12Z-octadecadienoyl)-sn-glycero-3-phosphocholine	11005824	CCCCC/C=C\C/C=C\CCCCCCCC(=O)OC[C@H](COP(=O)(OCC[N+](C)(C)C)[O-])O	$C_{26}H_{50}NO_7P$	519.65	7	1	114.93	0.49	-5.07	Moderately soluble	Low	No	Yes	Yes	1	0.55	0	5.94
MET 110	1-Hexadecanoyl-sn-glycero-3-phosphoethanolamine	9547069	CCCCCCCCCCCCCCCC(=O)OC[C@H](COP(=O)(OCCN)O)O	$C_{21}H_{44}NO_7P$	453.55	8	3	138.12	4.09	-2.62	Soluble	Low	No	Yes	Yes	0	0.55	0	5.47
MET 111	1-(1Z-octadecenyl)-sn-glycero-3-phosphoethanolamine	42607470	CCCCCCCCCCCCCCCC/C=C\OC[C@H](COP(=O)(OCCN)O)O	$C_{23}H_{48}NO_6P$	465.6	7	3	121.05	5.17	-3.66	Soluble	Low	No	Yes	Yes	0	0.55	0	5.94
MET 112	1-(9Z-octadecenoyl)-sn-glycero-3-phosphoethanolamine	9547071	CCCCCCCC/C=C\CCCCCCCC(=O)OC[C@H](COP(=O)(OCCN)O)O	$C_{23}H_{46}NO_7P$	479.59	8	3	138.12	4.32	-2.81	Soluble	Low	No	Yes	Yes	0	0.55	0	5.64
MET 113	1-(9Z,12Z-octadecadienoyl)-glycero-3-phosphoethanolamine	52925130	CCCCC/C=C\C/C=C\CCCCCCCC(=O)OC[C@H](COP(=O)(OCCN)O)O	$C_{23}H_{44}NO_7P$	477.57	8	3	138.12	4.32	-2.81	Soluble	Low	No	Yes	Yes	0	0.55	0	5.64
MET 114	Malic Acid	525	OC(=O)CC(C(=O)O)O	$C_4H_6O_5$	134.09	5	3	94.83	-0.01	0.32	Highly soluble	High	No	No	No	0	0.56	0	2.27
MET 115	D-Mannose	18950	OC[C@H]1OC(O)[C@H]([C@H]([C@@H]1O)O)O	$C_6H_{12}O_6$	180.16	6	5	110.38	0.31	1.15	Highly soluble	Low	No	Yes	No	0	0.55	0	4.08
MET 116	Methanol	887	CO	CH_4O	32.04	1	1	20.23	0.81	0.25	Highly soluble	Low	No	No	No	0	0.55	0	1

MET 117	Methionine	6137	CSCC[C@@H](C(=O)O)N	C5H11NO2S	149.21	3	2	88.62	1.12	0.68	Highly soluble	High	No	No	No	0	0.55	0	2.43
MET 118	Methyl 2-O-benzyl-d-arabinofuranoside	22215008	COC1O[C@@H]([C@H]([C@@H]1OCc1ccccc1)O)CO	C13H18O5	254.28	5	2	68.15	2.29	-1.62	Very soluble	High	No	No	No	0	0.55	0	3.96
MET 119	N-(4,6-Dimethyl-2-pyrimidinyl)-4-(4-nitrobenzylideneamino)benzenesulfonamide	542809	Cc1nc(nc(c1)C)NS(=O)(=O)c1ccc(cc1)/N=C/c1ccc(cc1)N(=O)=O	C19H17N5O4S	411.43	7	1	138.51	2.64	-3.77	Soluble	Low	No	No	Yes	0	0.55	0	3.26
MET 120	N,N-Dimethylglycine	673	CN(CC(=O)O)C	C4H9NO2	103.12	3	1	40.54	1	1.49	Highly soluble	High	No	No	No	0	0.55	0	1
MET 121	N-epsilon-Acetyl-L-lysine	92832	CC(=O)NCCCC[C@@H](C(=O)O)N	C8H16N2O3	188.22	4	3	92.42	1.22	0.88	Highly soluble	High	No	No	No	0	0.55	0	1.79
MET 122	N-epsilon-Acetyl-L-lysine	92832	CC(=O)NCCCC[C@@H](C(=O)O)N	C8H16N2O3	188.22	4	3	92.42	1.22	0.88	Highly soluble	High	No	No	No	0	0.55	0	1.79
MET 123	N-Acetyl-L-Aspartic Acid	65065	OC(=O)C[C@@H](C(=O)O)NC(=O)C	C6H9NO5	175.14	5	3	103.7	0.21	0.63	Highly soluble	High	No	No	No	0	0.56	0	2.11
MET 124	N-Acetyllysine	92907	CC(=O)N[C@H](C(=O)O)CCCCN	C8H16N2O3	188.22	4	3	92.42	0.56	2.26	Highly soluble	High	No	No	No	0	0.55	0	1.95
MET 125	N-Acetylputrescine	122356	NCCCCNC(=O)C	C6H14N2O	130.19	2	2	55.12	1.36	0.12	Highly soluble	High	No	No	No	0	0.55	0	1
MET 126	Nicotinamide-Adenine-Dinucleotide	5893	O[C@@H]1[C@H](O)[C@H](O[C@H]1[n+]1cccc(c1)C(=O)N)COP(=O)(OP(=O)(OC[C@H]1O[C@H]([C@@H]([C@@H]1O)O)n1cnc2c1ncnc2N)O)O	C21H28N7O14P2+	664.43	17	8	337.88	-6.01	0.24	Highly soluble	Low	No	Yes	No	3	0.11	0	6.14
MET 127	2-Phenyl-1,2,3,4-tetrahydronaphthalene	34581	c1ccc(cc1)C1CCc2c(C1)cccc2	C16H16	208.3	0	0	0	2.82	-4.46	Moderately soluble	Low	Yes	No	No	1	0.55	0	2.44
MET 128	N-Formylmethionine	439750	CSCC[C@@H](C(=O)O)NC=O	C6H11NO3S	177.22	3	2	91.7	0.9	-0.66	Very soluble	High	No	No	No	0	0.56	0	2.38
MET 129	Palmitic Acid	985	CCCCCCCCCCCCCCCC(=O)O	C16H32O2	256.42	2	1	37.3	3.85	-5.02	Moderately soluble	High	Yes	No	No	1	0.85	0	2.31
MET 130	Methyl Nicotinate	7151	COC(=O)c1cccnc1	C7H7NO2	137.14	3	0	39.19	1.61	-1.53	Very soluble	High	Yes	No	No	0	0.55	0	1.16

MET 131	Normetanephrine	1237	NCC(c1ccc(c(c1)OC)O)O	C9H13NO3	183.2	4	3	75.71	1.6	-0.46	Very soluble	High	No	No	No	0	0.55	0	2.01
MET 132	Stearic Acid	5281	CCCCCCCCCCCCCCCCCC(=O)O	C18H36O2	284.48	2	1	37.3	4.3	-5.73	Moderately soluble	High	No	No	No	1	0.85	0	2.54
MET 133	alpha-HEXYLCINNAMALDEHYDE	1550884	CCCCCC/C(=C\c1ccccc1)/C=O	C15H20O	216.32	1	0	17.07	3	-4.01	Moderately soluble	High	Yes	No	No	0	0.55	0	2.53
MET 134	Oleic Acid	445639	CCCCCCCC/C=C\CCCCCCCC(=O)O	C18H34O2	282.46	2	1	37.3	4.01	-5.41	Moderately soluble	High	No	No	No	1	0.85	0	3.07
MET 135	N,N,N-trimethyl-2-(phosphonooxy)ethanaminium	1014	OP(=O)(OCC[N+](C)(C)C)O	C5H15NO4P+	184.15	4	2	76.57	-2.54	0.23	Highly soluble	High	No	No	No	0	0.56	0	2.94
MET 136	Ornithine	6262	N[C@H](C(=O)O)CCCN	C5H12N2O2	132.16	4	3	89.34	0.49	2.38	Highly soluble	High	No	No	No	0	0.55	0	1.61
MET 137	Orotate	1492348	O=c1[nH]c(=O)[nH]c(c1)C(=O)[O-]	C5H3N2O4-	155.09	4	2	105.85	0.08	-0.62	Very soluble	Low	No	No	No	0	0.56	0	1.82
MET 138	2-Tyrosine	91482	OC(=O)C(Cc1ccccc1O)N	C9H11NO3	181.19	4	3	83.55	0.81	0	Highly soluble	High	No	No	No	0	0.55	0	1.77
MET 139	Oxime-, methoxy-phenyl-	9602988	CO/C(=N\O)/c1ccccc1	C8H9NO2	151.16	3	1	41.82	1.69	-2.33	Soluble	High	Yes	No	No	0	0.55	0	1.96
MET 140	O-Xylene	7237	Cc1ccccc1C	C8H10	106.17	0	0	0	2.03	-3.02	Soluble	Low	Yes	No	No	0	0.55	0	1
MET 141	Pantothenic Acid	6613	OCC([C@H](C(=O)NCCC(=O)O)O)(C)C	C9H17NO5	219.23	5	4	106.86	0.95	-0.06	Very soluble	High	No	No	No	0	0.56	0	2.44
MET 142	P-Cresol	2879	Cc1ccc(cc1)O	C7H8O	108.14	1	1	20.23	1.48	-2.29	Soluble	High	Yes	No	No	0	0.55	0	1
MET 143	Phenylalanine	6140	N[C@H](C(=O)O)Cc1ccccc1	C9H11NO2	165.19	3	2	63.32	1.08	-0.08	Very soluble	High	No	No	No	0	0.55	0	1.46
MET 144	Phosphate Ion	1061	[O-]P(=O)([O-])[O-]	O4P---	94.97	3	2	96.06	1.08	-0.08	Very soluble	High	No	No	No	0	0.55	0	1.46
MET 145	Phosphonoacetic Acid	546	OC(=O)CP(=O)(O)O	C2H5O5P	140.03	5	3	104.64	-0.79	0.69	Highly soluble	High	No	No	No	0	0.56	0	2.91
MET 146	phytosphingosine-1-P	53481399	CCCCCCCCCCCCCC[C@H]([C@@H]([C@@H](COP(=O)(O)O)N)O)O	C18H40NO6P	397.49	7	5	143.05	2.48	-1.91	Very soluble	Low	No	Yes	No	0	0.56	0	5.31
MET 147	Phytosphingosine	122121	CCCCCCCCCCCCCC[C@H]([C@H]([C@H](CO)N)O)O	C18H39NO3	317.51	4	4	86.71	3.84	-3.68	Soluble	High	No	Yes	No	0	0.55	0	4.01
MET 148	Pipecolic Acid	849	OC(=O)C1CCCCN1	C6H11NO2	129.16	3	2	49.33	1.16	0.88	Highly soluble	High	No	No	No	0	0.55	0	1.69

MET 149	Hydrazinecarboxamide, 2-3-methyl-6-(1-methylethyl)-2-cyclohexen-1-ylidene-	5705136	NC(=O)N/N=C/1\C=C(C)CCC1C(C)C	C11H19N3O	209.29	2	2	67.48	1.5	-1.82	Very soluble	High	Yes	No	No	0	0.55	0	4.18
MET 150	Proline	145742	OC(=O)[C@@H]1CCCN1	C5H9NO2	115.13	3	2	49.33	0.84	1.09	Highly soluble	High	No	No	No	0	0.55	0	1.54
MET 151	Propionate ion	104745	[O-]C(=O)CC	C3H5O2-	73.07	2	0	40.13	0.89	-0.43	Very soluble	High	No	No	No	0	0.85	0	1
MET 152	Purine	1044	c1ncc2c(n1)nc[nH]2	C5H4N4	120.11	3	1	54.46	0.31	-1.09	Very soluble	High	No	No	No	0	0.55	0	1.26
MET 153	Putrescine	1045	NCCCCN	C4H12N2	88.15	2	2	52.04	1.21	0.25	Highly soluble	High	No	No	No	0	0.55	0	1
MET 154	Pyroglutamic Acid	7405	O=C1CC[C@H](N1)C(=O)O	C5H7NO3	129.11	3	2	66.4	0.5	-0.09	Very soluble	High	No	No	No	0	0.85	0	1.67
MET 155	Pyruvate	107735	CC(=O)C(=O)[O-]	C3H3O3-	87.05	3	0	57.2	0.33	-0.11	Very soluble	High	No	No	No	0	0.85	0	1
MET 156	Xylitol	6912	OC[C@H](C([C@H](CO)O)O)O	C5H12O5	152.15	5	5	101.15	0.34	1.04	Highly soluble	Low	No	No	No	0	0.55	0	2.94
MET 157	D-Ribose	10975657	O[C@@H]1COC([C@@H]([C@@H]1O)O)O	C5H10O5	150.13	5	4	90.15	-2.58	1.13	Highly soluble	Low	No	No	No	0	0.55	0	3.8
MET 158	Sarcosine	1088	CNCC(=O)O	C3H7NO2	89.09	3	2	49.33	0.68	1.49	Highly soluble	High	No	No	No	0	0.55	0	1
MET 159	Serine	5951	OC[C@@H](C(=O)O)N	C3H7NO3	105.09	4	3	83.55	0.18	1.57	Highly soluble	High	No	No	No	0	0.55	0	1.51
MET 160	Shikimic Acid	8742	O[C@@H]1CC(=C[C@H]([C@H]1O)O)C(=O)O	C7H10O5	174.15	5	4	97.99	0.51	0.23	Highly soluble	High	No	No	No	0	0.56	0	3.77
MET 161	Spermine	1103	NCCCNCCCCNCCCN	C10H26N4	202.34	4	4	76.1	2.33	0.32	Highly soluble	High	No	No	No	0	0.55	0	1.8
MET 162	Sphinganine	91486	CCCCCCCCCCCCCCC[C@H]([C@H](CO)N)O	C18H39NO2	301.51	3	3	66.48	4.23	-4.31	Moderately soluble	High	Yes	Yes	No	0	0.55	0	3.42
MET 163	Sphingosine	5280335	CCCCCCCCCCCCC/C=C/[C@H]([C@H](CO)N)O	C18H37NO2	299.49	3	3	66.48	4.17	-4.03	Moderately soluble	High	Yes	Yes	No	0	0.55	0	4.46
MET 164	Succinate	160419	[O-]C(=O)CCC(=O)[O-]	C4H4O4--	116.07	4	0	80.26	0.34	0.01	Highly soluble	Low	No	No	No	0	0.56	0	1.23
MET 165	Sucrose	5988	OC[C@H]1O[C@@]([C@H]([C@@H]1O)O)(CO)O[C@H]1O[C@H](CO)[C@H]([C@@	C12H22O11	342.3	11	8	189.53	0.85	0.7	Highly soluble	Low	No	Yes	No	2	0.17	0	5.16

			H]([C@H]1O)O)O																
MET 166	2-Amino-4,4,6,6-tetramethyl-4,6-dihydrothieno(2,3-c)furan-3-carbonitrile	610214	N#Cc1c(N)sc2c1C(C)(C)OC2(C)C	C11H14N2OS	222.31	2	1	87.28	2.39	-2.7	Soluble	High	No	Yes	No	0	0.55	0	3.16
MET 167	Threonine	6288	C[C@H]([C@@H](C(=O)O)N)O	C4H9NO3	119.12	4	3	83.55	0.33	1.41	Highly soluble	High	No	No	No	0	0.55	0	1.79
MET 168	Trehalose	7427	OC[C@H]1O[C@H](O[C@H]2O[C@H](CO)[C@H]([C@@H]([C@H]2O)O)O)[C@@H]([C@H]([C@@H]1O)O)O	C12H22O11	342.3	11	8	189.53	0.98	0.94	Highly soluble	Low	No	Yes	No	2	0.17	0	5.22
MET 169	Tris(2,4-DI-tert-butylphenyl)phosphate	14572930	OC[C@H]1O[C@H](O[C@H]2O[C@H](CO)[C@H]([C@@H]([C@H]2O)O)O)[C@@H]([C@H]([C@@H]1O)O)O	C12H22O11	342.3	11	8	189.53	0.98	0.94	Highly soluble	Low	No	Yes	No	2	0.17	0	5.22
MET 170	Tryptophan	6305	OC(=O)[C@H](Cc1c[nH]c2c1cccc2)N	C11H12N2O2	204.23	3	3	79.11	0.99	-0.68	Very soluble	High	No	No	No	0	0.55	0	2.09
MET 171	Tyrosine	6057	OC(=O)[C@H](Cc1ccc(cc1)O)N	C9H11NO3	181.19	4	3	83.55	0.84	0.32	Highly soluble	High	No	No	No	0	0.55	0	1.54
MET 172	Uridine Monophosphate	6030	O[C@@H]1[C@H](O)[C@H](O[C@H]1n1ccc(=O)[nH]c1=O)COP(=O)(O)O	C9H13N2O9P	324.18	9	5	181.12	-0.4	0.49	Highly soluble	Low	No	No	No	1	0.11	0	4.27
MET 173	Uracil	1174	O=c1cc[nH]c(=O)[nH]1	C4H4N2O2	112.09	2	2	65.72	0.52	-0.42	Very soluble	High	No	No	No	0	0.55	0	1.35
MET 174	Uridine	6029	OC[C@H]1O[C@H]([C@@H]([C@@H]1O)O)n1ccc(=O)[nH]c1=O	C9H12N2O6	244.2	6	4	124.78	0.44	-0.24	Very soluble	Low	No	No	No	0	0.55	0	3.76
MET 175	Valine	6287	N[C@H](C(=O)O)C(C)C	C5H11NO2	117.15	3	2	63.32	1.03	0.99	Highly soluble	High	No	No	No	0	0.55	0	1.19
MET 176	Z-8-Methyl-9-tetradecenoic acid	5364410	CCCC/C=C\C(CCCCCCC(=O)O)C	C15H28O2	240.38	2	1	37.3	3.45	-4.03	Moderately soluble	High	Yes	No	No	0	0.85	0	3.19
MET 177	alpha-D-glucopyranosyl-(1->3)-alpha-D-fructofuranosyl	16058661	OC[C@H]1O[C@H](O[C@H]2[C@H](O)[C@H](O[C@]2(CO)O[C@	C18H32O16	504.44	16	11	268.68	-0.64	1.25	Highly soluble	Low	No	Yes	No	3	0.17	0	6.23

	alpha-D-galactopyranoside		H]2O[C@H](CO)[C@@H]([C@@H]([C@H]2O)O)O)CO)[C@@H]([C@H]([C@@H]1O)O)O																
MET 178	beta-Hydroxyquebrachamine	624530	CCC12CCC(CN2CCc2c(CC1)[nH]c1c2cccc1)O	C19H26N2O	298.42	2	2	39.26	2.8	-4.08	Moderately soluble	High	Yes	Yes	No	0	0.55	1	3.61
MET 179	Gamma-Aminobutyric Acid	119	NCCCC(=O)O	C4H9NO2	103.12	3	2	63.32	0.72	1.72	Highly soluble	High	No	No	No	0	0.55	0	1
MET 180	gamma-Linolenic acid	5280933	CCCCC/C=C\C/C=C\C/C=C\CCCCC(=O)O	C18H30O2	278.43	2	1	37.3	0.72	1.72	Highly soluble	High	No	No	No	0	0.55	0	1
MET 181	Farnesol	3327	OCC=C(CCC=C(CCC=C(C)C)C)C	C15H26O	222.37	1	1	20.23	3.71	-4.17	Moderately soluble	High	Yes	No	No	0	0.55	0	3.17
MET 182	Gliotoxin	6223	OC[C@@]12SS[C@@]3(N(C1=O)[C@@H]1[C@@H](O)C=CC=C1C3)C(=O)N2C	C13H14N2O4S2	326.39	4	2	131.68	2.07	-1.34	Very soluble	High	No	Yes	No	0	0.55	0	5.63
MET 183	Beta-Glucan	439262	OC[C@H]1O[C@@H](OC2[C@@H](CO)O[C@@H]([C@@H]([C@H]2O)O)O)[C@@H]([C@H](C1O[C@@H]1O[C@H](CO)[C@H]([C@@H]([C@H]1O)O)O)O)O	C18H32O16	504.44	16	11	268.68	-1.84	1.83	Highly soluble	Low	No	Yes	No	3	0.17	0	6.45
MET 184	Lanosterol	246983	CC(=CCC[C@H]([C@H]1CC[C@@]2([C@]1(C)CCC1=C2CC[C@@H]2[C@]1(C)CC[C@@H](C2(C)C)O)C)C)C	C30H50O	426.72	1	1	20.23	5.17	-7.83	Poorly soluble	Low	No	No	No	1	0.55	0	6.07
MET 185	Eburicol	9803310	CC(C(=C)CC[C@H]([C@H]1CC[C@@]2([C@]1(C)CCC1=C2CC[C@@H]2[C@]1(C)CC[C@@H](C2(C)C)O)C)C)C	C31H52O	440.74	1	1	20.23	5.25	-8.19	Poorly soluble	Low	No	No	No	1	0.55	0	6.19

Compound Identifier (CID); Simplified Molecular Input Line Entry System (SMILES); Molecular Weight (MW); Hydrogen Bond Acceptor (HBA); Hydrogen Bond Donor (HBD); Topological Polar Surface Area (TPSA); Implicit LOGP (iLOGP); Estimated Solubility (ESOL); Water solubility (log scale); Water solubility classification (ESOL Class); Gastrointestinal Absorption (GI Abs.); Blood-Brain Barrier (BBB); P-glycoprotein (Pgp); Cytochrome P450 3A4 (CYP3A4); Lipinski's Rule of 5 (RO5); Bioavailability Score (BS); Pan Assay Interference Compounds (PAINS); Synthetic Accessibility (SA).

Supplementary Table S2. Ranking of overlapping targets in the MET–TAR network by degree metric.

Rank	Gene	Degree	Rank	Gene	Degree	Rank	Gene	Degree	Rank	Gene	Degree
1	PTGS1	5	45	JAK1	2	89	MAP2K1	1	133	CHRNA4	1
2	PTGS2	5	46	JAK2	2	90	HDAC1	1	134	GRIN2B	1
3	AR	4	47	PIK3CD	2	91	MTOR	1	135	MCL1	1
4	AHR	3	48	PIK3CB	2	92	FLT4	1	136	RAC1	1
5	GSK3B	3	49	PIK3CG	2	93	CCND3	1	137	CACNA1B	1
6	CASP9	3	50	LIPE	2	94	MAPK10	1	138	TNF	1
7	BCL2A1	3	51	CDK9	2	95	FLT1	1	139	TYMS	1
8	GRM5	3	52	TRPA1	2	96	KIT	1	140	PPARG	1
9	ACHE	3	53	PRKCG	2	97	MMP2	1	141	PPARD	1
10	HDAC6	3	54	NOS2	2	98	CCND1	1	142	FABP3	1
11	PGR	3	55	NOS3	2	99	CCND2	1	143	FABP4	1
12	PIK3CA	3	56	DNMT1	2	100	MAOA	1	144	PTPN1	1
13	MMP3	3	57	CNR1	2	101	IDH1	1	145	PTPN2	1
14	MMP9	3	58	PRKCH	2	102	ELANE	1	146	FAAH	1
15	APOBEC3G	3	59	G6PD	2	103	ADAM17	1	147	FABP1	1
16	TLR9	3	60	SIGMAR1	2	104	MMP1	1	148	TERT	1
17	APOBEC3A	3	61	HSD11B2	2	105	P2RX7	1	149	HMGCR	1
18	HDAC4	3	62	IMPDH2	2	106	JAK3	1	150	CDC25A	1
19	ADORA2A	2	63	STS	2	107	CAPN1	1	151	PTPN11	1
20	CDK2	2	64	MDM2	2	108	PTK2	1	152	MAPK3	1
21	PARP1	2	65	GABRA2	1	109	PRKDC	1	153	NR3C2	1
22	NTRK1	2	66	MAPK8	1	110	HCK	1	154	BCHE	1
23	CDK4	2	67	DRD1	1	111	CCR3	1	155	NR1I3	1
24	MIF	2	68	HTT	1	112	PARP4	1	156	EDNRA	1
25	ALOX5	2	69	HTR3A	1	113	LRRK2	1	157	ACE	1
26	DRD2	2	70	PDPK1	1	114	PLAT	1	158	PLA2G2A	1
27	KDR	2	71	VCP	1	115	DRD4	1	159	ANPEP	1

28	EGFR	2	72	AKR1B1	1	116	SYK	1	160	HDAC3	1
29	TRPV1	2	73	DRD3	1	117	AURKA	1	161	HDAC2	1
30	AKT1	2	74	BRAF	1	118	PDE5A	1	162	PTPRC	1
31	HDAC8	2	75	ERBB2	1	119	TACR1	1	163	GPBAR1	1
32	IDO1	2	76	MET	1	120	PDE3B	1	164	PER2	1
33	FYN	2	77	OPRM1	1	121	GSTP1	1	165	PRKCD	1
34	NR3C1	2	78	VEGFA	1	122	HCRTR2	1	166	PRKCA	1
35	ESR1	2	79	FGFR1	1	123	STAT3	1	167	UGT2B7	1
36	MTNR1B	2	80	SPHK2	1	124	CREBBP	1	168	PABPC1	1
37	DYRK1A	2	81	KIF11	1	125	PIM1	1	169	NR1H2	1
38	CDK5	2	82	FLT3	1	126	PDE4A	1	170	PRKCB	1
39	MAOB	2	83	RET	1	127	SHH	1	171	PRKCE	1
40	SIRT1	2	84	LCK	1	128	NAMPT	1	172	PRKCQ	1
41	RELA	2	85	MAPK14	1	129	HTR1A	1	173	RASGRP1	1
42	CHRM2	2	86	TTR	1	130	TRHR	1	174	PSEN1	1
43	CHRM1	2	87	MAPK1	1	131	AURKB	1	175	CSNK2A1	1
44	CMA1	2	88	KCNMA1	1	132	ITGA4	1	176	NOS1	1

Supplementary Table S3. The top 30 GO biological processes (BP) enriched with the overlapping genes between the predicted metabolite targets and HIV latency-associated host genes.

GO ID	BP	Enrichment FDR	nGenes	Pathway Genes	Fold Enrichment	Genes
GO:0010243	Response to organonitrogen compound	1.72E-45	71	1061	8.650569498	GSK3B JAK2 JAK3 DRD2 DRD3 JAK1 VCP STAT3 HTR1A DRD1 LRRK2 PPARG DRD4 P2RX7 SIRT1 AHR ADORA2A MDM2 PARP1 CACNA1B PRKCD HTR3A PTK2 PTPN11 MTOR FYN PTGS2 MMP2 MMP9 PLAT PIK3CG CCND1 OPRM1 MAPK14 CCND3 BCHE TACR1 CNR1 PIK3CA FLT3 PRKCG DNMT1 CDK4 AKT1 EGFR EDNRA CDK5 SYK CHRM1 GRM5 PRKCE RELA PTPN2 HSD11B2 TYMS CHRM2 PTPN1 HDAC2 TRPV1 TNF TLR9 PSEN1 ITGA4 PRKCQ MAPK1 MAPK3 PDPK1 PRKDC PDE3B PRKCB MMP3
GO:1901701	Cellular response to oxygen-containing compound	1.26E-55	85	1272	8.638404754	GSK3B JAK2 JAK3 DRD2 DRD3 JAK1 CHRM1 STAT3 HTR1A CHRM2 DRD1 LRRK2 PPARG SPHK2 DRD4 ESR1 HDAC6 SIRT1 MMP9 TRPA1 AHR MAPK8 MAPK14 MDM2 PARP1 EGFR FABP1 PRKCD NOS3 HTR3A AR PTK2 RELA PTPN11 GPBAR1 MTOR TNF NOS2 PTGS2 ACHE MMP2 CMA1 MAPK1 MAPK3 PLAT PIK3CG PPARD OPRM1 CCND3 PIK3CA DNMT1 CASP9 CDK4 AKT1 SIGMAR1 EDNRA ACE CDK5 PRKCB GRM5 PRKCE PTPN2 PTPN1 HDAC2 TRPV1 TLR9 MIF PSEN1 ITGA4 FYN PRKCQ MET NR3C1 PIM1 PDPK1 PRKCA VCP RET PRKDC GSTP1 PDE3B P2RX7 HCK CDK2 MMP3
GO:1901698	Response to nitrogen compound	7.81E-45	73	1172	8.051874241	GSK3B JAK2 JAK3 DRD2 DRD3 JAK1 VCP CHRM1 STAT3 HTR1A CHRM2 DRD1 LRRK2 PPARG DRD4 P2RX7 SIRT1 AHR ADORA2A MDM2 PARP1 CACNA1B PRKCD HTR3A PTK2 PTPN11 MTOR FYN PTGS2 ACHE MMP2 MAPK1 MMP9 MAPK3 PLAT PIK3CG CCND1 OPRM1 MAPK14 CCND3 BCHE TACR1 CNR1 PIK3CA FLT3 PRKCG DNMT1 CDK4 AKT1 EGFR EDNRA CDK5 SYK GRM5 PRKCE RELA

						PTPN2 HSD11B2 TYMS PTPN1 HDAC2 TRPV1 TNF TLR9 PSEN1 ITGA4 PRKCQ PDPK1 PRKDC PDE3B PRKCB CDK2 MMP3
GO:1901700	Response to oxygen-containing compound	5.13E-62	101	1752	7.452277301	NOS2 GSK3B NOS1 JAK2 JAK3 DRD2 DRD3 JAK1 NOS3 CHRM1 STAT3 HTR1A CHRM2 DRD1 LRRK2 PPARG SPHK2 DRD4 P2RX7 ESR1 HDAC6 SIRT1 MMP9 TRPA1 AHR MAPK8 MAPK14 MDM2 PARP1 EGFR CACNA1B ADAM17 KCNMA1 FABP1 PRKCD HTR3A AR PTK2 RELA PTPN11 GPBAR1 MTOR TNF GRIN2B FYN MAOB PTGS2 ACHE MMP2 CMA1 MAPK1 MAPK3 PLAT PIK3CG CCND1 PPARD OPRM1 CCND3 BCHE TACR1 CNR1 PIK3CA DNMT1 IDO1 CASP9 CDK4 AKT1 SIGMAR1 EDNRA ACE G6PD CDK5 PRKCB GRM5 PRKCE PTPN2 HSD11B2 TYMS PTPN1 HDAC2 TRPV1 ELANE NTRK1 TLR9 MIF PSEN1 ITGA4 PRKCQ MET NR3C1 PIM1 PDPK1 PRKCA VCP RET PRKDC GSTP1 PDE3B HCK CDK2 MMP3
GO:0043067	Reg. of programmed cell death	9.12E-53	90	1627	7.150833915	SPHK2 PSEN1 JAK2 JAK3 MAPK8 MCL1 EGFR TNF PIK3CA MTOR GSK3B GSTP1 HDAC6 SIRT1 VEGFA KDR PIM1 AKT1 BRAF FABP1 AR RELA AURKB LRRK2 HTT MIF PIK3CB HDAC4 PTGS2 PTPRC AKR1B1 MMP2 AURKA P2RX7 ESR1 MMP9 PIK3CG CCND1 PPARD NR3C1 HDAC1 CNR1 PRKCG ADORA2A IDO1 PPARG CASP9 MTNR1B MDM2 BCL2A1 PDPK1 PARP1 DRD3 KIT ACE G6PD PRKCD TERT SHH CDK5 PTK2 PTPN2 PTPN1 TRPV1 NTRK1 PRKDC NOS2 PRKCH FLT4 PRKCQ CSNK2A1 HCK MET ITGA4 CCND2 DNMT1 SIGMAR1 PRKCA KCNMA1 SYK RET PIK3CD LCK ADAM17 NOS3 HDAC2 FGFR1 FLT3 VCP HDAC3
GO:0006468	Protein phosphorylation	1.12E-52	91	1684	6.985556987	PRKCH PRKCQ PTPRC JAK2 CSNK2A1 JAK3 CCND1 CCND3 VEGFA CCND2 CDK2 PRKCG CDK4 CDK9 PIM1 PDPK1 AKT1 PRKCA KIT DYRK1A JAK1 PRKCD CDK5 PRKCB PTK2 PRKCE LRRK2 MTOR AURKB FLT4 DRD4 FGFR1 GSK3B GSTP1 AURKA SIRT1 MAPK1 FLT1 MAPK3

						PIK3CG MAPK8 KDR PPARG ERBB2 EGFR ADAM17 BRAF SYK MAP2K1 HDAC3 RASGRP1 PTPN2 LCK PTPN1 NTRK1 TNF TLR9 MIF PRKDC FYN PIK3CB PTGS2 PSEN1 P2RX7 HDAC6 HCK MET MAPK10 MAPK14 HMGCR PIK3CA FLT3 ADORA2A PDE5A DRD2 EDNRA ACE RET GRM5 FABP4 DRD1 HDAC2 NOS1 MMP9 RAC1 PTPN11 HTT PIK3CD ELANE LIPE CDC25A
GO:0019220	Reg. of phosphate metabolic proc.	2.71E-44	79	1478	6.909623632	NOS2 FLT4 FGFR1 PTPRC NOS1 FLT1 MET CCND1 CCND3 VEGFA CCND2 FLT3 KDR ERBB2 EGFR KIT NOS3 RET NTRK1 FABP3 AURKB LRRK2 MTOR DRD4 PSEN1 GSTP1 SIRT1 MAPK1 PIK3CG PPARG PDPK1 AKT1 ADAM17 BRAF PRKCD MAP2K1 HDAC3 RASGRP1 PTPN2 PTPN1 TNF TLR9 MIF FYN PIK3CB SPHK2 HDAC4 PTGS2 P2RX7 HDAC6 JAK2 HMGCR PIK3CA ADORA2A IDH1 PDE5A DRD2 ACE CDK5 SYK STAT3 GRM5 PTK2 FABP4 PRKCE DRD1 HDAC2 PRKDC MMP9 MAPK3 RAC1 PARP1 VCP AR PTPN11 HTT ELANE GSK3B CDC25A
GO:0051174	Reg. of phosphorus metabolic proc.	2.75E-44	79	1479	6.904951811	NOS2 FLT4 FGFR1 PTPRC NOS1 FLT1 MET CCND1 CCND3 VEGFA CCND2 FLT3 KDR ERBB2 EGFR KIT NOS3 RET NTRK1 FABP3 AURKB LRRK2 MTOR DRD4 PSEN1 GSTP1 SIRT1 MAPK1 PIK3CG PPARG PDPK1 AKT1 ADAM17 BRAF PRKCD MAP2K1 HDAC3 RASGRP1 PTPN2 PTPN1 TNF TLR9 MIF FYN PIK3CB SPHK2 HDAC4 PTGS2 P2RX7 HDAC6 JAK2 HMGCR PIK3CA ADORA2A IDH1 PDE5A DRD2 ACE CDK5 SYK STAT3 GRM5 PTK2 FABP4 PRKCE DRD1 HDAC2 PRKDC MMP9 MAPK3 RAC1 PARP1 VCP AR PTPN11 HTT ELANE GSK3B CDC25A
GO:0042981	Reg. of apoptotic proc.	3.65E-48	85	1594	6.893381962	SPHK2 PSEN1 JAK2 JAK3 MAPK8 MCL1 EGFR TNF PIK3CA MTOR GSK3B GSTP1 SIRT1 VEGFA KDR PIM1 AKT1 BRAF FABP1 AR RELA AURKB HTT MIF PIK3CB HDAC4 PTGS2 PTPRC AKR1B1 MMP2 AURKA P2RX7 ESR1 MMP9 PIK3CG CCND1 PPARD NR3C1 HDAC1 CNR1 PRKCG ADORA2A IDO1 PPARG CASP9 MTNR1B MDM2

						BCL2A1 PDPK1 PARP1 DRD3 ACE G6PD PRKCD TERT SHH CDK5 PTK2 PTPN2 PTPN1 TRPV1 NTRK1 PRKDC PRKCH FLT4 PRKCQ CSNK2A1 HCK ITGA4 CCND2 DNMT1 SIGMAR1 PRKCA KCNMA1 RET PIK3CD LCK LRRK2 ADAM17 NOS3 HDAC2 FGFR1 FLT3 VCP HDAC3
GO:0009719	Response to endogenous stimulus	6.25E-49	87	1660	6.775056157	NOS2 PGR GSK3B NOS1 ESR1 JAK2 JAK3 PPARD NR3C1 PPARG DRD2 DRD3 NR3C2 JAK1 NOS3 STAT3 AR HTR1A DRD1 LRRK2 NTRK1 HDAC1 HDAC2 DRD4 FGFR1 SIRT1 MAPK1 MAPK3 AHR CCND1 MDM2 PDPK1 PARP1 PRKCD HTR3A PTK2 PTPN11 MTOR TNF FYN HDAC4 MAOB PTGS2 MMP2 PLAT PIK3CG OPRM1 MAPK14 CCND3 BCHE TACR1 PIK3CA FLT3 KDR DNMT1 CASP9 CDK4 IDH1 AKT1 EGFR EDNRA KIT ACE CDK5 SYK CHRM1 GRM5 PRKCE PTPN2 HSD11B2 TYMS CHRM2 PTPN1 TRPV1 TLR9 PSEN1 ITGA4 CREBBP PRKCQ ERBB2 ADAM17 RELA PRKDC PDE3B PRKCB P2RX7 HDAC6
GO:0016310	Phosphorylation	7.81E-51	95	1994	6.15885793	PRKCH FLT4 SPHK2 PRKCQ FGFR1 PTPRC JAK2 CSNK2A1 FLT1 JAK3 MET CCND1 CCND3 VEGFA CCND2 FLT3 CDK2 PRKCG KDR CDK4 CDK9 PIM1 PDPK1 ERBB2 AKT1 EGFR PRKCA KIT DYRK1A JAK1 PRKCD CDK5 RET PRKCB PTK2 PRKCE LRRK2 NTRK1 MTOR AURKB PIK3CB DRD4 PSEN1 GSK3B GSTP1 AURKA SIRT1 MAPK1 MAPK3 PIK3CG MAPK8 PIK3CA PPARG ADAM17 BRAF SYK MAP2K1 PIK3CD HDAC3 RASGRP1 PTPN2 LCK PTPN1 TNF TLR9 MIF PRKDC FYN HDAC4 PTGS2 P2RX7 HDAC6 HCK MAPK10 MAPK14 HMGCR ADORA2A PDE5A DRD2 EDNRA ACE STAT3 GRM5 FABP4 DRD1 HDAC2 NOS1 MMP9 RAC1 AR PTPN11 HTT ELANE LIPE CDC25A
GO:0042127	Reg. of cell population proliferation	1.62E-44	86	1835	6.058486122	VEGFA CDK2 ERBB2 EGFR SYK STAT3 PTK2 JAK2 ACE FGFR1 CSNK2A1 AHR KDR PPARG PIM1 AKT1 ADAM17 SHH MAP2K1 AR RELA ELANE NTRK1 TNF TLR9 MIF NOS2 ALOX5 FLT4 SPHK2 PRKCQ HDAC4 PTGS2 PTPRC PGR MMP2 ESR1 PTGS1 FLT1 JAK3 CCND1 PPARD

						MAPK14 CCND3 BCHE TACR1 HDAC1 PIK3CA FLT3 ADORA2A DNMT1 IDO1 CDK4 PDE5A DRD2 DRD3 KIT BRAF TERT NOS3 GPBAR1 CCR3 HDAC2 MAPK1 MAPK3 PIK3CD PRKCH SIRT1 MMP9 HCK ITGA4 CCND2 PDPK1 PRKCA PTPN2 LRRK2 PRKDC GSTP1 PER2 CAPN1 NAMPT OPRM1 FABP3 MDM2 CHRM1 HTR1A
GO:0012501	Programmed cell death	1.63E-51	98	2127	5.956077231	SPHK2 PSEN1 GSK3B JAK2 JAK3 MAPK8 CASP9 BCL2A1 MCL1 EGFR CDK5 TNF PRKDC PIK3CA MTOR GSTP1 HDAC6 SIRT1 VEGFA KDR MDM2 PIM1 AKT1 PARP1 BRAF FABP1 PRKCD AR RELA AURKB LRRK2 HTT MIF PIK3CB HDAC4 PTGS2 PTPRC AKR1B1 MMP2 AURKA P2RX7 ESR1 MAPK1 MMP9 CSNK2A1 MAPK3 PIK3CG CCND1 PPARD MAPK14 NR3C1 HDAC1 CNR1 PRKCG ADORA2A IDO1 PPARG MTNR1B PDPK1 DRD3 EDNRA PRKCA KIT ACE G6PD TERT SHH PRKCB PTK2 PTPN2 PTPN1 TRPV1 NTRK1 NOS2 PRKCH FLT4 PRKCQ HCK MET ITGA4 CCND2 DNMT1 SIGMAR1 KCNMA1 SYK RET PIK3CD LCK ADAM17 NOS3 HDAC2 FGFR1 AHR FLT3 VCP PRKCE HDAC3 ELANE
GO:0008283	Cell population proliferation	2.96E-51	98	2143	5.911608153	VEGFA CDK2 ERBB2 EGFR SYK STAT3 PTK2 JAK2 ACE FGFR1 CSNK2A1 AHR KDR PPARG PIM1 AKT1 ADAM17 SHH MAP2K1 AR RELA ELANE NTRK1 TNF TLR9 MIF NOS2 FYN ALOX5 FLT4 PIK3CB SPHK2 PRKCQ HDAC4 PTGS2 PSEN1 PTPRC PGR MMP2 P2RX7 ESR1 PTGS1 MAPK1 FLT1 JAK3 CCND1 PPARD MAPK14 CCND3 BCHE TACR1 HDAC1 PIK3CA FLT3 ADORA2A DNMT1 IDO1 CDK4 PDE5A DRD2 DRD3 KIT BRAF PRKCD TERT NOS3 IMPDH2 AURKB GPBAR1 CCR3 HDAC2 MAPK3 PIK3CD PRKCH SIRT1 MMP9 HCK ITGA4 CCND2 PDPK1 PRKCA RASGRP1 PTPN2 LRRK2 PRKDC GSTP1 PER2 CAPN1 NAMPT PIK3CG OPRM1 FABP3 MDM2 CDK9 EDNRA CDC25A CHRM1 HTR1A

GO:0006915	Apoptotic proc.	1.67E-46	92	2065	5.75929741	SPHK2 PSEN1 GSK3B JAK2 JAK3 MAPK8 CASP9 BCL2A1 MCL1 EGFR CDK5 TNF PRKDC PIK3CA MTOR GSTP1 SIRT1 VEGFA KDR MDM2 PIM1 AKT1 PARP1 BRAF FABP1 PRKCD AR RELA AURKB HTT MIF PIK3CB HDAC4 PTGS2 PTPRC AKR1B1 MMP2 AURKA P2RX7 ESR1 MAPK1 MMP9 CSNK2A1 MAPK3 PIK3CG CCND1 PPARD MAPK14 NR3C1 HDAC1 CNR1 PRKCG ADORA2A IDO1 PPARG MTNR1B PDPK1 DRD3 EDNRA PRKCA ACE G6PD TERT SHH PRKCB PTK2 PTPN2 PTPN1 TRPV1 NTRK1 PRKCH FLT4 PRKCQ HCK ITGA4 CCND2 DNMT1 SIGMAR1 KCNMA1 RET PIK3CD LCK LRRK2 ADAM17 NOS3 HDAC2 FGFR1 AHR FLT3 VCP PRKCE HDAC3
GO:0008219	Cell death	3.00E-53	103	2320	5.739194915	SPHK2 PSEN1 GSK3B JAK2 JAK3 MAPK8 CASP9 BCL2A1 MCL1 EGFR CDK5 TNF PRKDC PIK3CA MTOR GSTP1 HDAC6 SIRT1 VEGFA KDR MDM2 PIM1 AKT1 PARP1 BRAF FABP1 PRKCD AR RELA AURKB LRRK2 HTT MIF FYN PIK3CB HDAC4 PTGS2 PTPRC AKR1B1 MMP2 AURKA P2RX7 ESR1 MAPK1 MMP9 CSNK2A1 MAPK3 PIK3CG CCND1 PPARD MAPK14 NR3C1 HDAC1 CNR1 PRKCG ADORA2A IDO1 PPARG MTNR1B PDPK1 SIGMAR1 DRD2 DRD3 EDNRA PRKCA KIT ACE G6PD TERT SHH PRKCB PTK2 PTPN2 PTPN1 TRPV1 NTRK1 NOS2 PRKCH FLT4 PRKCQ HCK PARP4 MET ITGA4 CCND2 DNMT1 KCNMA1 SYK RET PIK3CD LCK MMP3 ADAM17 NOS3 HDAC2 GRIN2B FGFR1 AHR FLT3 VCP PRKCE HDAC3 ELANE
GO:0071310	Cellular response to organic substance	9.28E-50	104	2609	5.153010115	PGR GSK3B ESR1 JAK2 JAK3 PPARD VEGFA NR3C1 FLT3 PPARG DRD2 DRD3 NR3C2 KIT JAK1 VCP STAT3 AR HTR1A DRD1 LRRK2 NTRK1 TNF GSTP1 HDAC1 HDAC2 FLT4 SPHK2 DRD4 FGFR1 SIRT1 FLT1 AHR MAPK8 MAPK14 KDR MDM2 CDK9 PDPK1 ERBB2 AKT1 PARP1 EGFR PRKCD HTR3A PTK2 RELA PTPN11 GPBAR1 PTPN1 MTOR NOS2 FYN HDAC4 PTGS2 PTPRC MMP2 P2RX7 CMA1 HDAC6 MAPK1 MAPK3 TRPA1 PLAT PIK3CG

						CCND1 OPRM1 CCND3 ITGA4 PIK3CA DNMT1 CASP9 CDK4 EDNRA ACE NOS3 CDK5 SYK PRKCB CHRM1 GRM5 FABP4 PRKCE PTPN2 IMPDH2 CHRM2 CCR3 TRPV1 TLR9 MIF PIK3CB PSEN1 PIK3CD CREBBP PRKCQ PIM1 ADAM17 PRKCA RET PRKDC NOS1 PDE3B NR1H2 HCK
GO:0010033	Response to organic substance	1.96E-59	123	3269	4.863981625	NOS2 PGR GSK3B NOS1 ESR1 JAK2 JAK3 PPARD VEGFA NR3C1 FLT3 PPARG DRD2 DRD3 NR3C2 KIT JAK1 NOS3 VCP CHRM1 STAT3 AR RELA HTR1A CHRM2 DRD1 LRRK2 NTRK1 TNF GSTP1 HDAC1 HDAC2 FLT4 SPHK2 DRD4 FGFR1 P2RX7 SIRT1 MAPK1 FLT1 MAPK3 AHR MAPK8 CCND1 MAPK14 KDR ADORA2A MDM2 CDK9 PDPK1 ERBB2 AKT1 MCL1 PARP1 EGFR CACNA1B ADAM17 KCNMA1 PRKCD HTR3A PTK2 PTPN11 GPBAR1 PTPN1 MTOR GRIN2B FYN HDAC4 MAOB PTGS2 PTPRC ACHE MMP2 CMA1 HDAC6 MMP9 TRPA1 PLAT PIK3CG OPRM1 CCND3 BCHE ITGA4 TACR1 CNR1 PIK3CA PRKCG DNMT1 IDO1 CASP9 CDK4 IDH1 EDNRA ACE G6PD CDK5 SYK PRKCB GRM5 FABP4 PRKCE PTPN2 HSD11B2 TYMS IMPDH2 CCR3 TRPV1 ELANE TLR9 MIF PIK3CB PSEN1 PIK3CD CREBBP PRKCQ PIM1 PRKCA RET PRKDC PDE3B NR1H2 HCK MMP3
GO:0070887	Cellular response to chemical stimulus	3.92E-59	123	3300	4.818289676	PGR GSK3B ESR1 JAK2 JAK3 PPARD VEGFA NR3C1 FLT3 PPARG DRD2 DRD3 NR3C2 KIT JAK1 VCP CHRM1 STAT3 AR HTR1A CHRM2 CCR3 DRD1 LRRK2 NTRK1 TNF GSTP1 HDAC1 ADAM17 HDAC2 MTOR FLT4 SPHK2 DRD4 FGFR1 AKR1B1 HDAC6 SIRT1 MMP9 FLT1 TRPA1 AHR MAPK8 MAPK14 KDR APOBEC3A MDM2 CDK9 PDPK1 ERBB2 AKT1 PARP1 EGFR BRAF FABP1 PRKCD NOS3 SYK HTR3A PTK2 RELA PTPN11 GPBAR1 PTPN1 MIF NOS2 FYN ALOX5 PIK3CB PDE4A HDAC4 PTGS2 PTPRC ACHE MMP2 P2RX7 CMA1 PTGS1 MAPK1 MAPK3 PLAT PIK3CG CCND1 OPRM1 CCND3 ITGA4 PIK3CA DNMT1 CASP9 CDK4 MCL1 SIGMAR1 EDNRA ACE TERT SHH CDK5 PRKCB GRM5

						FABP4 PRKCE PTPN2 IMPDH2 TRPV1 TLR9 PSEN1 PIK3CD CREBBP PRKCQ MET RAC1 PIM1 PRKCA G6PD RET PRKDC NOS1 MMP3 PDE3B NR1H2 HCK BCHE CDK2
GO:0006796	Phosphate-containing compound metabolic proc.	5.89E-50	110	3005	4.732056742	NOS2 PRKCH FLT4 PIK3CB SPHK2 PRKCQ FGFR1 PTPRC NOS1 JAK2 CSNK2A1 FLT1 JAK3 NAMPT PIK3CG MET CCND1 CCND3 VEGFA CCND2 PIK3CA FLT3 CDK2 PRKCG KDR IDO1 CDK4 CDK9 PIM1 PDPK1 ERBB2 AKT1 EGFR PRKCA KIT DYRK1A G6PD JAK1 PRKCD NOS3 CDK5 RET PRKCB PTK2 PRKCE PIK3CD TYMS IMPDH2 PLA2G2A LRRK2 NTRK1 MTOR FABP3 AURKB PDE4A DRD4 PSEN1 GSK3B GSTP1 AURKA SIRT1 MAPK1 MAPK3 MAPK8 PPARG ADAM17 BRAF SYK MAP2K1 HDAC3 RASGRP1 PTPN2 PTPN11 LCK PTPN1 TNF TLR9 MIF PRKDC FYN HDAC4 PTGS2 P2RX7 HDAC6 HCK MAPK10 PPARD MAPK14 HMGCR ADORA2A NR1H2 IDH1 PDE5A DRD2 EDNRA ACE CDC25A STAT3 GRM5 FABP4 DRD1 HDAC2 MMP9 RAC1 PARP1 VCP AR HTT ELANE LIPE
GO:0032879	Reg. of localization	7.39E-48	107	2945	4.696779949	GSK3B PPARD VEGFA TACR1 PPARG RAC1 DRD2 DRD3 KIT SYK PTK2 LRRK2 DRD4 HDAC6 HDAC1 ADAM17 AURKB DRD1 HDAC2 GRIN2B FGFR1 PTPRC P2RX7 SIRT1 MMP9 FLT1 MAPK8 KDR NR1H2 CDK9 PDPK1 ERBB2 AKT1 EGFR EDNRA PRKCA BRAF TERT SHH NOS3 VCP RET PRKCB AR HDAC3 PTPN11 PTPN1 HTT TNF TLR9 MIF FYN ALOX5 FLT4 PIK3CB SPHK2 HDAC4 MAOB PTGS2 PSEN1 MMP2 JAK2 MAPK1 MAPK3 TRPA1 JAK3 PIK3CG OPRM1 MAPK14 HMGCR ITGA4 CNR1 FABP3 PRKCG ADORA2A MTNR1B PARP1 CACNA1B KCNMA1 ACE G6PD CDK5 CHRM1 STAT3 PRKCE PTPN2 HTR1A CHRM2 TRPV1 ELANE GRM5 PIK3CD NOS2 HCK MET PIM1 HDAC8 PRKCD MTOR NOS1 CHRNA4 PER2 LCK CAPN1 PRKCH ACHE MAP2K1
GO:0006793	Phosphorus metabolic proc.	1.19E-49	110	3030	4.693013369	NOS2 PRKCH FLT4 PIK3CB SPHK2 PRKCQ FGFR1 PTPRC NOS1 JAK2 CSNK2A1 FLT1 JAK3 NAMPT PIK3CG MET

						CCND1 CCND3 VEGFA CCND2 PIK3CA FLT3 CDK2 PRKCG KDR IDO1 CDK4 CDK9 PIM1 PDPK1 ERBB2 AKT1 EGFR PRKCA KIT DYRK1A G6PD JAK1 PRKCD NOS3 CDK5 RET PRKCB PTK2 PRKCE PIK3CD TYMS IMPDH2 PLA2G2A LRRK2 NTRK1 MTOR FABP3 AURKB PDE4A DRD4 PSEN1 GSK3B GSTP1 AURKA SIRT1 MAPK1 MAPK3 MAPK8 PPARG ADAM17 BRAF SYK MAP2K1 HDAC3 RASGRP1 PTPN2 PTPN11 LCK PTPN1 TNF TLR9 MIF PRKDC FYN HDAC4 PTGS2 P2RX7 HDAC6 HCK MAPK10 PPARD MAPK14 HMGCR ADORA2A NR1H2 IDH1 PDE5A DRD2 EDNRA ACE CDC25A STAT3 GRM5 FABP4 DRD1 HDAC2 MMP9 RAC1 PARP1 VCP AR HTT ELANE LIPE
GO:0051239	Reg. of multicellular organismal proc.	9.06E-48	108	3024	4.616828087	FLT1 VEGFA KDR KCNMA1 SYK CHRM2 TLR9 JAK2 PLAT HDAC1 PPARG ACE HDAC2 GRIN2B NOS2 FGFR1 PTPRC GSTP1 P2RX7 SIRT1 MAPK14 RAC1 PIM1 AKT1 DRD2 ADAM17 PRKCA KIT NOS3 ELANE TNF MIF ALOX5 PRKCH FLT4 PIK3CB SPHK2 PRKCQ HDAC4 PTGS2 PSEN1 ACHE MMP2 ESR1 CMA1 MAPK1 MMP9 MAPK3 PIK3CG CCND1 PPARD OPRM1 HMGCR TACR1 FAAH CNR1 PIK3CA ADORA2A IDO1 NR1H2 MTNR1B CDK4 MDM2 CDK9 PDE5A PDPK1 ERBB2 PARP1 DRD3 EDNRA BRAF G6PD JAK1 PRKCD TERT SHH CDK5 RET PRKCB STAT3 GRM5 MAP2K1 AR PTK2 FABP4 PRKCE RASGRP1 RELA PTPN2 HTR1A PTPN11 CCR3 LRRK2 TRPV1 NTRK1 MTOR PRKDC PIK3CD HDAC6 MET EGFR PDE3B NOS1 JAK3 PER2 HCRTR2 HDAC3 GSK3B
GO:0035556	Intracellular signal transduction	1.94E-43	101	2847	4.5860168	NOS2 PRKCH PIK3CB PRKCQ NOS1 JAK2 MAPK1 MAPK3 JAK3 PIK3CG MET MAPK8 MAPK10 MAPK14 PIK3CA PRKCG CASP9 RAC1 BCL2A1 PDPK1 ERBB2 AKT1 MCL1 PDE3B PRKCA KIT BRAF JAK1 PRKCD NOS3 PRKCB MAP2K1 PRKCE PIK3CD RASGRP1 RELA CCR3 LRRK2 NTRK1 MTOR TNF TLR9 PRKDC AURKB FYN DRD4 FGFR1 GSK3B GSTP1 ESR1 SIRT1 FLT1 AHR CCND1

						OPRM1 VEGFA KDR PPARG MDM2 EGFR DRD2 EDNRA SYK RET LCK PLA2G2A MIF FLT4 SPHK2 PDE4A HDAC4 PTGS2 PSEN1 PTPRC P2RX7 PPARD HMGCR HDAC1 MTNR1B PDE5A PARP1 DYRK1A SHH GRM5 AR PTPN2 PTPN11 GPBAR1 PTPN1 GRIN2B CDK2 MMP9 DNMT1 NR3C2 PTK2 HDAC3 HTT DRD3 ELANE AURKA FLT3
GO:0010646	Reg. of cell communication	3.62E-46	114	3602	4.091314618	GSK3B MET ERBB2 MCL1 DRD2 DRD3 PDE3B KIT GRM5 LRRK2 NTRK1 TNF GRIN2B GSTP1 ACHE HDAC1 NOS3 AURKB HDAC2 TLR9 DRD4 FGFR1 ESR1 SIRT1 JAK2 FLT1 PIK3CG OPRM1 VEGFA KDR PPARG MDM2 PDPK1 EGFR CACNA1B ADAM17 BRAF SHH VCP RET AR RELA PTPN2 PTPN11 PLA2G2A PTPN1 HTT MIF FYN ALOX5 FLT4 PIK3CB SPHK2 PDE4A PTGS2 PSEN1 PTPRC P2RX7 MAPK1 MAPK3 PLAT PPARD MAPK14 CCND3 HMGCR BCHE TACR1 CNR1 PRKCG ADORA2A MTNR1B CDK4 PDE5A AKT1 PARP1 DYRK1A ACE PRKCD TERT CDK5 SYK PRKCB STAT3 MAP2K1 PTK2 PRKCE RASGRP1 HTR1A GPBAR1 CHRM2 DRD1 MAOA MTOR PIK3CA PIK3CD CREBBP NOS2 PRKCH PRKCQ HDAC6 MMP9 CSNK2A1 NR3C2 PRKCA HDAC3 LCK PER2 NR1H2 ELANE CAPN1 AURKA JAK3 FLT3 RAC1
GO:0023051	Reg. of signaling	5.04E-46	114	3615	4.07660173	GSK3B MET ERBB2 MCL1 DRD2 DRD3 PDE3B KIT GRM5 LRRK2 NTRK1 TNF GRIN2B GSTP1 ACHE HDAC1 NOS3 AURKB HDAC2 TLR9 DRD4 FGFR1 ESR1 SIRT1 JAK2 FLT1 PIK3CG OPRM1 VEGFA KDR PPARG MDM2 PDPK1 EGFR CACNA1B ADAM17 BRAF SHH VCP RET AR RELA PTPN2 PTPN11 PLA2G2A PTPN1 HTT MIF FYN ALOX5 FLT4 PIK3CB SPHK2 PDE4A PTGS2 PSEN1 PTPRC P2RX7 MAPK1 MAPK3 PLAT PPARD MAPK14 CCND3 HMGCR BCHE TACR1 CNR1 PRKCG ADORA2A MTNR1B CDK4 PDE5A AKT1 PARP1 DYRK1A ACE PRKCD TERT CDK5 SYK PRKCB STAT3 MAP2K1 PTK2 PRKCE RASGRP1 HTR1A GPBAR1 CHRM2 DRD1 MAOA MTOR PIK3CA PIK3CD

						CREBBP NOS2 PRKCH PRKCQ HDAC6 MMP9 CSNK2A1 NR3C2 PRKCA HDAC3 LCK PER2 NR1H2 ELANE CAPN1 AURKA JAK3 FLT3 RAC1
GO:0065008	Reg. of biological quality	4.60E-47	121	4103	3.812287	GSK3B NOS2 PSEN1 ACHE NOS1 JAK2 CHRNA4 JAK3 BCHE RAC1 CACNA1B DRD2 DRD3 GABRA2 KCNMA1 ACE NOS3 SYK HTR3A ANPEP CCR3 LRRK2 TRPV1 GRIN2B DRD4 PLAT DRD1 PABPC1 P2RX7 SIRT1 TRPA1 OPRM1 VEGFA TACR1 KDR MDM2 HCRTR2 PDPK1 HDAC8 EDNRA PRKCA TERT UGT2B7 HDAC3 HTT TNF FYN ALOX5 PIK3CB PRKCQ MAOB PTGS2 LIPE PTPRC AKR1B1 MMP2 ESR1 CMA1 HDAC6 PTGS1 MAPK1 MAPK3 PIK3CG CCND1 PPARD MAPK14 NR3C1 FAAH TTR CNR1 FABP3 PIK3CA PRKCG ADORA2A NR1H2 PPARG MTNR1B PIM1 AKT1 PARP1 KIT BRAF PRKCD SHH CDK5 VCP RET PRKCB CHRM1 STAT3 GRM5 AR PTK2 FABP4 PRKCE PTPN2 HSD11B2 HTR1A PTPN11 CHRM2 MAOA NTRK1 MTOR TLR9 MIF PRKDC LCK CREBBP AURKA HCK MET G6PD PIK3CD FLT3 PER2 ADAM17 PLA2G2A GSTP1 MCL1 ELANE MAPK8
GO:0042221	Response to chemical	2.39E-58	140	4821	3.753985916	NOS2 PGR GSK3B NOS1 ESR1 JAK2 CHRNA4 JAK3 PPARD VEGFA NR3C1 FLT3 PPARG RAC1 DRD2 DRD3 NR3C2 KIT JAK1 NOS3 VCP CHRM1 STAT3 AR RELA HTR1A CHRM2 CCR3 DRD1 LRRK2 NTRK1 TNF GSTP1 HDAC1 ADAM17 HDAC2 MTOR FLT4 SPHK2 DRD4 FGFR1 AKR1B1 P2RX7 HDAC6 SIRT1 MAPK1 MMP9 FLT1 MAPK3 TRPA1 MET AHR MAPK8 CCND1 MAPK14 KDR ADORA2A APOBEC3A MDM2 CDK9 PDPK1 ERBB2 AKT1 MCL1 PARP1 EGFR CACNA1B KCNMA1 BRAF FABP1 PRKCD SYK HTR3A PTK2 PTPN11 GPBAR1 LCK PTPN1 MIF GRIN2B FYN ALOX5 PIK3CB PDE4A HDAC4 MAOB PTGS2 PTPRC ACHE MMP2 CMA1 PTGS1 PLAT PIK3CG OPRM1 CCND3 BCHE ITGA4 TACR1 CNR1 PIK3CA PRKCG DNMT1 IDO1 CASP9 CDK4 IDH1 SIGMAR1 EDNRA ACE G6PD TERT SHH CDK5

						RET PRKCB GRM5 FABP4 PRKCE PIK3CD PTPN2 HSD11B2 TYMS IMPDH2 TRPV1 ELANE TLR9 PSEN1 CREBBP PRKCQ PIM1 PRKCA PRKDC MMP3 PDE3B PARP4 NR1H2 HCK CDK2 MAP2K1
GO:0048583	Reg. of response to stimulus	1.37E-43	120	4338	3.57596643	FYN GSK3B MET PPARD VEGFA PPARG ERBB2 MCL1 PDE3B KIT SYK LCK LRRK2 NTRK1 TNF TLR9 GSTP1 JAK2 PLAT HDAC1 ADAM17 PRKCD NOS3 AURKB HDAC2 GRIN2B ALOX5 DRD4 FGFR1 PTPRC ESR1 CMA1 HDAC6 SIRT1 FLT1 PIK3CG AHR OPRM1 PRKCG KDR MDM2 CDK9 PDPK1 PARP1 EGFR DRD3 BRAF SHH VCP RET AR PTK2 RELA PTPN2 PTPN11 PLA2G2A PTPN1 HTT APOBEC3G MIF PRKDC FLT4 PIK3CB SPHK2 PRKCQ PDE4A HDAC4 PTGS2 PSEN1 P2RX7 MAPK1 MAPK3 MAPK14 CCND3 HMGCR CNR1 FLT3 ADORA2A IDO1 NR1H2 MTNR1B CDK4 PDE5A AKT1 DRD2 DYRK1A ACE G6PD TERT CDK5 PRKCB STAT3 GRM5 MAP2K1 FABP4 PRKCE RASGRP1 GPBAR1 MAOA ELANE MTOR PIK3CA PIK3CD CREBBP PRKCH MMP9 CSNK2A1 CASP9 RAC1 NR3C2 PRKCA HDAC3 JAK3 MMP3 DRD1 CAPN1 AURKA HCK MAPK8 MAPK10

Supplementary Table S4. The top 30 GO cellular components (CC) enriched with the overlapping genes between the predicted metabolite targets and HIV latency-associated host genes.

GO ID	CC	Enrichment FDR	nGenes	Pathway Genes	Fold Enrichment	Genes
GO:0005901	Caveola	4.68E-08	10	91	14.20562488	HDAC6 HCK KCNMA1 NOS3 LRRK2 PTGS2 LIPE JAK2 MAPK1 MAPK3
GO:0044853	Plasma membrane raft	7.57E-07	10	124	10.42509568	HDAC6 HCK KCNMA1 NOS3 LRRK2 PTGS2 LIPE JAK2 MAPK1 MAPK3
GO:0099699	Integral component of synaptic membrane	8.02E-07	11	160	8.887394068	DRD2 PSEN1 OPRM1 CNR1 ADORA2A DRD3 GABRA2 CHRM1 GRM5 CHRM2 DRD1
GO:0045121	Membrane raft	4.48E-13	23	351	8.470761505	PSEN1 LRRK2 FYN HDAC6 HCK KDR EGFR ADAM17 KCNMA1 NOS3 LCK TNF PTGS2 LIPE PTPRC JAK2 MAPK1 MAPK3 OPRM1 CNR1 SHH RET NOS1
GO:0098857	Membrane microdomain	4.48E-13	23	352	8.446696841	PSEN1 LRRK2 FYN HDAC6 HCK KDR EGFR ADAM17 KCNMA1 NOS3 LCK TNF PTGS2 LIPE PTPRC JAK2 MAPK1 MAPK3 OPRM1 CNR1 SHH RET NOS1
GO:0098978	Glutamatergic synapse	5.12E-09	18	343	6.783910659	GSK3B DRD2 RAC1 DRD4 JAK2 PLAT MAPK14 CNR1 ADORA2A DRD3 CDK5 VCP CHRM1 GRM5 RELA CHRM2 DRD1 LRRK2
GO:0045211	Postsynaptic membrane	1.43E-07	15	288	6.732874294	KCNMA1 GRM5 GRIN2B CHRNA4 OPRM1 ADORA2A SIGMAR1 DRD2 DRD3 GABRA2 HTR3A CHRM1 CHRM2 DRD1 TRPV1
GO:0043235	Receptor complex	1.18E-10	22	429	6.629291612	FLT4 FGFR1 FLT1 MET AHR ITGA4 FLT3 KDR ERBB2 EGFR GABRA2 KIT RET NTRK1 GRIN2B JAK2 CHRNA4 PPARG NR3C2 SYK HTR3A GPBAR1
GO:0097060	Synaptic membrane	4.22E-08	18	395	5.890838876	DRD2 KCNMA1 GRM5 GRIN2B PSEN1 CHRNA4 OPRM1 CNR1 PRKCG ADORA2A SIGMAR1 DRD3 GABRA2 HTR3A CHRM1 CHRM2 DRD1 TRPV1
GO:0098794	Postsynapse	4.48E-13	30	664	5.840565652	NOS1 RAC1 GABRA2 KCNMA1 GRM5 PTK2 GRIN2B SIGMAR1 HTT FYN DRD4 GSK3B P2RX7 JAK2 CHRNA4 PLAT

						OPRM1 PRKCG ADORA2A PDPK1 AKT1 DRD2 DRD3 CDK5 HTR3A CHRM1 CHRM2 DRD1 LRRK2 TRPV1
GO:0030425	Dendrite	1.68E-12	29	653	5.74098684	DRD4 PSEN1 RAC1 GABRA2 CHRM1 PTK2 HTR1A CHRM2 LRRK2 HTT FYN NOS1 HDAC6 OPRM1 TACR1 PRKCG ADORA2A DRD2 DYRK1A CDK5 RET GRM5 DRD1 TRPV1 NTRK1 MTOR GSK3B CHRNA4 MAPK8
GO:0097447	Dendritic tree	1.68E-12	29	655	5.72345711	DRD4 PSEN1 RAC1 GABRA2 CHRM1 PTK2 HTR1A CHRM2 LRRK2 HTT FYN NOS1 HDAC6 OPRM1 TACR1 PRKCG ADORA2A DRD2 DYRK1A CDK5 RET GRM5 DRD1 TRPV1 NTRK1 MTOR GSK3B CHRNA4 MAPK8
GO:0036477	Somatodendritic compartment	2.63E-13	35	888	5.095148114	DRD4 PSEN1 RAC1 CACNA1B GABRA2 CHRM1 PTK2 HTR1A CHRM2 LRRK2 HTT FYN AURKA P2RX7 NOS1 HDAC6 OPRM1 ITGA4 TACR1 HDAC1 PRKCG ADORA2A DRD2 DYRK1A CDK5 RET GRM5 DRD1 TRPV1 NTRK1 MTOR TNF GSK3B CHRNA4 MAPK8
GO:0044297	Cell body	2.59E-08	22	588	4.836677044	PSEN1 CACNA1B LRRK2 FYN AURKA P2RX7 HDAC6 OPRM1 ITGA4 TACR1 HDAC1 ADORA2A DRD2 GABRA2 BRAF CDK5 RET CHRM2 TRPV1 NTRK1 TNF CHRNA4
GO:0043025	Neuronal cell body	3.36E-07	19	513	4.78782172	PSEN1 CACNA1B LRRK2 AURKA P2RX7 HDAC6 OPRM1 ITGA4 HDAC1 ADORA2A DRD2 GABRA2 CDK5 RET CHRM2 TRPV1 NTRK1 TNF CHRNA4
GO:0030424	Axon	2.55E-07	22	676	4.207050446	PSEN1 GSK3B RET LRRK2 NTRK1 HTT AURKA HDAC6 OPRM1 ITGA4 CNR1 PRKCG ADORA2A SIGMAR1 DRD2 GABRA2 DYRK1A CDK5 PRKCB CHRM1 CHRM2 MAPK8
GO:0045202	Synapse	2.63E-13	44	1435	3.963715821	GSK3B PSEN1 NOS1 CHRNA4 RAC1 DRD2 GABRA2 KCNMA1 HTR3A CHRM1 GRM5 PTK2 CHRM2 GRIN2B ACHE SIGMAR1 HTT FYN HDAC4 DRD4 P2RX7 JAK2 MAPK1 PLAT MAPK8 OPRM1 MAPK14 CNR1 PRKCG ADORA2A MTNR1B HCRTR2 PDPK1 AKT1 CACNA1B DRD3 CDK5 VCP PRKCB RELA HTR1A DRD1 LRRK2 TRPV1
GO:0070161	Anchoring junction	5.92E-08	27	926	3.769246257	CAPN1 PABPC1 FLT1 ITGA4 RAC1 EGFR JAK1 PTK2 JAK2 VEGFA ADAM17 PRKCH PTPRC P2RX7 MAPK1 HCK MAPK3

						CCND1 OPRM1 PIK3CA PRKCG CDK4 PDPK1 AKT1 KIT PRKCD MAP2K1
GO:0030054	Cell junction	3.33E-18	64	2293	3.608092426	GSK3B CAPN1 PABPC1 FLT1 ITGA4 RAC1 EGFR JAK1 PSEN1 NOS1 CHRNA4 DRD2 GABRA2 KCNMA1 HTR3A CHRM1 GRM5 PTK2 CHRM2 GRIN2B ACHE JAK2 NAMPT VEGFA SIGMAR1 ADAM17 CDK5 HTT FYN PRKCH HDAC4 DRD4 PTPRC P2RX7 MAPK1 HCK MAPK3 PLAT MAPK8 CCND1 OPRM1 MAPK14 CNR1 PIK3CA PRKCG KDR ADORA2A MTNR1B CDK4 HCRTR2 PDPK1 AKT1 CACNA1B DRD3 KIT PRKCD VCP PRKCB RELA HTR1A DRD1 LRRK2 TRPV1 MAP2K1
GO:0043005	Neuron projection	1.60E-10	39	1440	3.501094633	DRD4 PTGS2 PSEN1 GSK3B PTGS1 CHRNA4 OPRM1 RAC1 GABRA2 RET HTR3A CHRM1 PTK2 HTR1A CHRM2 LRRK2 NTRK1 HTT FYN AURKA NOS1 HDAC6 ITGA4 TACR1 CNR1 PRKCG ADORA2A SIGMAR1 DRD2 DYRK1A BRAF CDK5 PRKCB GRM5 DRD1 TRPV1 MTOR MAPK8 GRIN2B
GO:0098590	Plasma membrane region	4.19E-09	35	1332	3.396765409	PSEN1 MET ERBB2 EGFR DRD2 GABRA2 KCNMA1 GRM5 GRIN2B HDAC6 HCK RAC1 ADAM17 NOS3 HTR3A DRD1 LRRK2 TLR9 PDE4A PTGS2 LIPE JAK2 MAPK1 CHRNA4 MAPK3 OPRM1 CNR1 PRKCG ADORA2A SIGMAR1 DRD3 ACE CHRM1 CHRM2 TRPV1
GO:0005887	Integral component of plasma membrane	1.97E-10	45	1894	3.071385106	FLT4 DRD4 FGFR1 PSEN1 CHRNA4 FLT1 MET OPRM1 ITGA4 FLT3 KDR ADORA2A HCRTR2 ERBB2 EGFR DRD2 DRD3 GABRA2 KIT RET HTR3A CHRM1 GRM5 HTR1A GPBAR1 CHRM2 DRD1 TRPV1 NTRK1 GRIN2B P2RX7 JAK2 RAC1 ADAM17 KCNMA1 ACE TNF PTPRC CNR1 TRPA1 TACR1 MTNR1B EDNRA TRHR CCR3
GO:0031226	Intrinsic component of plasma membrane	7.48E-10	45	1978	2.940952169	FLT4 DRD4 FGFR1 PSEN1 CHRNA4 FLT1 MET OPRM1 ITGA4 FLT3 KDR ADORA2A HCRTR2 ERBB2 EGFR DRD2 DRD3 GABRA2 KIT RET HTR3A CHRM1 GRM5 HTR1A GPBAR1 CHRM2 DRD1 TRPV1 NTRK1 GRIN2B P2RX7 JAK2 RAC1 ADAM17 KCNMA1 ACE TNF PTPRC CNR1 TRPA1 TACR1 MTNR1B EDNRA TRHR CCR3

GO:0042995	Cell projection	1.18E-10	53	2477	2.765996319	DRD4 PTGS2 PSEN1 GSK3B PTGS1 CHRNA4 OPRM1 TACR1 RAC1 GABRA2 RET HTR3A CHRM1 PTK2 HTR1A CHRM2 LRRK2 NTRK1 PTPRC AURKA HDAC6 PDPK1 EGFR DRD2 ADAM17 DRD1 HTT FYN PDE4A PABPC1 NOS1 MAPK1 HCK MAPK3 ITGA4 CNR1 PIK3CA PRKCG ADORA2A AKT1 SIGMAR1 DRD3 DYRK1A BRAF ACE CDK5 PRKCB GRM5 TRPV1 MTOR P2RX7 MAPK8 GRIN2B
GO:0120025	Plasma membrane bounded cell projection	5.93E-10	50	2360	2.738796323	DRD4 PTGS2 PSEN1 GSK3B PTGS1 CHRNA4 OPRM1 TACR1 RAC1 GABRA2 RET HTR3A CHRM1 PTK2 HTR1A CHRM2 LRRK2 NTRK1 PTPRC AURKA HDAC6 EGFR DRD2 ADAM17 DRD1 HTT FYN PDE4A PABPC1 NOS1 MAPK1 MAPK3 ITGA4 CNR1 PIK3CA PRKCG ADORA2A AKT1 SIGMAR1 DYRK1A BRAF ACE CDK5 PRKCB GRM5 TRPV1 MTOR P2RX7 MAPK8 GRIN2B
GO:0031410	Cytoplasmic vesicle	1.60E-10	57	2849	2.586331213	RAC1 LRRK2 FYN CMA1 VEGFA CDK2 KDR ERBB2 EGFR GABRA2 ACE PRKCD RET PTPN1 HTT ELANE MTOR FGFR1 PSEN1 MAPK1 HCK FLT1 MAPK3 PLAT OPRM1 PDPK1 SIGMAR1 DRD2 DRD3 KIT SYK PLA2G2A NTRK1 TNF TLR9 GRIN2B ALOX5 CAPN1 PTPRC GSTP1 HDAC6 JAK2 MMP9 JAK3 MAPK14 TTR FLT3 MDM2 IDH1 JAK1 NOS3 VCP ANPEP MAP2K1 IMPDH2 CHRM2 MIF
GO:0097708	Intracellular vesicle	1.60E-10	57	2851	2.584516881	RAC1 LRRK2 FYN CMA1 VEGFA CDK2 KDR ERBB2 EGFR GABRA2 ACE PRKCD RET PTPN1 HTT ELANE MTOR FGFR1 PSEN1 MAPK1 HCK FLT1 MAPK3 PLAT OPRM1 PDPK1 SIGMAR1 DRD2 DRD3 KIT SYK PLA2G2A NTRK1 TNF TLR9 GRIN2B ALOX5 CAPN1 PTPRC GSTP1 HDAC6 JAK2 MMP9 JAK3 MAPK14 TTR FLT3 MDM2 IDH1 JAK1 NOS3 VCP ANPEP MAP2K1 IMPDH2 CHRM2 MIF
GO:0031982	Vesicle	1.18E-10	75	4466	2.170922298	CAPN1 PRKCH PABPC1 PTPRC GSTP1 AKR1B1 PTGS1 MMP9 PARP4 PLAT NAMPT ITGA4 TTR FABP3 RNASE1 RAC1 IDH1 PRKCA ACE G6PD FABP1 PRKCD VCP PRKCB ANPEP FABP4 IMPDH2 LCK PLA2G2A LRRK2 ELANE MIF NOS2 NOS1 NOS3 FYN CMA1 VEGFA CDK2 KDR ERBB2 AKT1 EGFR

						GABRA2 RET PTPN1 HTT MTOR FGFR1 PSEN1 MAPK1 HCK FLT1 MAPK3 OPRM1 PDPK1 SIGMAR1 DRD2 DRD3 KIT SYK NTRK1 TNF TLR9 GRIN2B ALOX5 HDAC6 JAK2 JAK3 MAPK14 FLT3 MDM2 JAK1 MAP2K1 CHRM2
GO:0005654	Nucleoplasm	1.52E-08	71	4581	2.003548185	CREBBP HDAC4 HDAC6 SIRT1 HDAC1 HDAC8 HDAC3 HDAC2 ALOX5 FLT4 PIK3CB SPHK2 PDE4A PSEN1 GSK3B AKR1B1 AURKA NOS1 JAK2 CSNK2A1 PARP4 NAMPT CCND1 MAPK14 CCND3 NR3C1 CCND2 CDK2 APOBEC3A PPARG PER2 CDK4 MDM2 CDK9 PIM1 AKT1 NR1I3 PARP1 NR3C2 FABP1 TERT CDK5 VCP PRKCB STAT3 AR PTPN2 HSD11B2 AURKB HTT MIF PRKDC PGR ESR1 PPARD NR1H2 MCL1 DYRK1A MTOR NOS2 MAPK1 MAPK3 AHR MAPK8 MAPK10 DNMT1 PRKCA PRKCD CDC25A RELA PTPN11
GO:0031981	Nuclear lumen	7.17E-08	73	4973	1.897606397	CREBBP HDAC4 HDAC6 SIRT1 HDAC1 PARP1 HDAC8 HDAC3 HDAC2 ALOX5 FLT4 PIK3CB SPHK2 PDE4A PSEN1 GSK3B AKR1B1 AURKA NOS1 JAK2 CSNK2A1 PARP4 NAMPT CCND1 MAPK14 CCND3 NR3C1 CCND2 CDK2 APOBEC3A PPARG PER2 CDK4 MDM2 CDK9 PIM1 AKT1 NR1I3 NR3C2 KIT FABP1 TERT CDK5 VCP PRKCB STAT3 AR PTPN2 HSD11B2 AURKB HTT MIF PRKDC PGR ESR1 PPARD NR1H2 MCL1 DYRK1A PRKCD TYMS MTOR NOS2 MAPK1 MAPK3 AHR MAPK8 MAPK10 DNMT1 PRKCA CDC25A RELA PTPN11

Supplementary Table S5. The top 30 GO molecular functions (MF) enriched with the overlapping genes between the predicted metabolite targets and HIV latency-associated host genes.

GO ID	MF	Enrichment FDR	nGenes	Pathway Genes	Fold Enrichment	Genes
GO:0004713	Protein tyrosine kinase activity	3.32E-21	23	159	18.69960559	FYN JAK2 HCK KDR JAK1 SYK PTK2 LCK FLT4 FGFR1 FLT1 JAK3 MET FLT3 ERBB2 EGFR KIT RET NTRK1 DYRK1A BRAF PRKCD MAP2K1
GO:0001221	Transcription coregulator binding	1.34E-11	14	120	15.08163842	PER2 AR TERT CREBBP PGR ESR1 HDAC6 AHR PPARD HDAC1 PPARG CDK9 HDAC3 RELA
GO:0004712	Protein serine/threonine/tyrosine kinase activity	8.55E-40	50	470	13.75225388	MAP2K1 PRKCG AKT1 FYN PRKCH FLT4 PRKCQ FGFR1 GSK3B AURKA JAK2 MAPK1 CSNK2A1 HCK FLT1 MAPK3 JAK3 PIK3CG MET MAPK8 MAPK10 MAPK14 PIK3CA FLT3 CDK2 KDR CDK4 CDK9 PIM1 PDPK1 ERBB2 EGFR PRKCA KIT DYRK1A BRAF JAK1 PRKCD CDK5 SYK RET PRKCB PTK2 PRKCE AURKB LCK LRRK2 NTRK1 MTOR PRKDC
GO:0106310	Protein serine kinase activity	2.37E-22	32	376	11.0018031	PIK3CB PIK3CG PRKCD PRKCH PRKCQ GSK3B AURKA MAPK1 CSNK2A1 MAPK3 MAPK8 MAPK10 MAPK14 PIK3CA CDK2 PRKCG CDK4 CDK9 PIM1 PDPK1 AKT1 PRKCA DYRK1A BRAF CDK5 PRKCB MAP2K1 PRKCE AURKB LRRK2 MTOR PRKDC
GO:0004672	Protein kinase activity	2.44E-37	53	632	10.84077988	FYN PRKCQ AURKA JAK2 HCK KDR AKT1 PRKCA JAK1 PRKCD SYK PRKCB PTK2 AURKB LCK LRRK2 MTOR PRKCH FLT4 FGFR1 GSK3B MAPK1 CSNK2A1 FLT1 MAPK3 JAK3 MET MAPK8 MAPK10 MAPK14 FLT3 CDK2 PRKCG CDK4 CDK9 PIM1 PDPK1 ERBB2 EGFR KIT DYRK1A BRAF CDK5 RET MAP2K1 PRKCE NTRK1 PRKDC PIK3CB PIK3CG CCND1 CCND3 PIK3CA
GO:0016773	Phosphotransferase activity alcohol group as acceptor	4.73E-36	55	748	9.505234297	FYN PRKCQ AURKA JAK2 HCK KDR AKT1 PRKCA JAK1 PRKCD SYK PRKCB PTK2 AURKB LCK LRRK2 MTOR PRKCH FLT4 PIK3CB FGFR1 GSK3B

						MAPK1 CSNK2A1 FLT1 MAPK3 JAK3 PIK3CG MET MAPK8 MAPK10 MAPK14 PIK3CA FLT3 CDK2 PRKCG CDK4 CDK9 PIM1 PDPK1 ERBB2 EGFR KIT DYRK1A BRAF CDK5 RET MAP2K1 PRKCE PIK3CD NTRK1 PRKDC SPHK2 CCND1 CCND3
GO:0004674	Protein serine/threonine kinase activity	1.28E-21	34	471	9.331678002	PRKCQ AURKA AKT1 PRKCA PRKCD PRKCB AURKB LRRK2 MTOR PRKCH GSK3B MAPK1 CSNK2A1 MAPK3 MAPK8 MAPK10 MAPK14 CDK2 PRKCG CDK4 CDK9 PIM1 PDPK1 DYRK1A BRAF CDK5 PRKCE PRKDC SYK PIK3CG CCND3 PIK3CA MAP2K1 EGFR
GO:0016301	Kinase activity	2.78E-33	55	849	8.374458486	FYN PRKCQ AURKA JAK2 HCK KDR AKT1 PRKCA JAK1 PRKCD SYK PRKCB PTK2 AURKB LCK LRRK2 MTOR PRKCH FLT4 PIK3CB SPHK2 FGFR1 GSK3B MAPK1 CSNK2A1 FLT1 MAPK3 JAK3 PIK3CG MET MAPK8 MAPK10 MAPK14 PIK3CA FLT3 CDK2 PRKCG CDK4 CDK9 PIM1 PDPK1 ERBB2 EGFR KIT DYRK1A BRAF CDK5 RET MAP2K1 PRKCE PIK3CD NTRK1 PRKDC CCND1 CCND3
GO:0016772	Transferase activity transferring phosphorus-containing groups	1.27E-30	56	1008	7.18173258	FYN PRKCQ AURKA JAK2 HCK KDR AKT1 PRKCA JAK1 PRKCD SYK PRKCB PTK2 AURKB LCK LRRK2 MTOR PRKCH FLT4 PIK3CB SPHK2 FGFR1 GSK3B MAPK1 CSNK2A1 FLT1 MAPK3 JAK3 PIK3CG MET MAPK8 MAPK10 MAPK14 PIK3CA FLT3 CDK2 PRKCG CDK4 CDK9 PIM1 PDPK1 ERBB2 EGFR KIT DYRK1A BRAF TERT CDK5 RET MAP2K1 PRKCE PIK3CD NTRK1 PRKDC CCND1 CCND3
GO:0019901	Protein kinase binding	1.44E-16	35	739	6.12245132	RAC1 PDE3B JAK2 CASP9 PTK2 HDAC4 PTPRC CCND1 CCND3 NR3C1 CDK9 AKT1 CDK5 SYK STAT3 GRM5 RELA PTPN11 GSK3B AURKA ESR1

						SIRT1 MAPK8 MAPK14 CCND2 KIF11 PARP1 ACE PRKCD CDC25A PRKCB PTPN2 LCK PTPN1 GSTP1
GO:0019900	Kinase binding	1.46E-18	39	824	6.118417805	RAC1 PDE3B JAK2 CASP9 PTK2 HDAC4 PTPRC CCND1 CCND3 NR3C1 CDK9 AKT1 CDK5 SYK STAT3 GRM5 RELA PTPN11 NTRK1 GSK3B AURKA ESR1 SIRT1 MAPK8 MAPK14 CCND2 KIF11 PARP1 EGFR ACE PRKCD CDC25A PRKCB PTPN2 AURKB LCK PTPN1 HTT GSTP1
GO:0008134	Transcription factor binding	3.83E-13	29	639	5.86676746	PER2 CREBBP PPARG PRKCB AR MTOR HDAC4 PPARD MAPK14 HDAC1 FLT3 NR1H2 PARP1 TERT PTPN2 PGR GSK3B ESR1 HDAC6 SIRT1 AHR CDK9 PIM1 STAT3 HDAC3 RELA HDAC2 PRKDC HDAC8
GO:0005524	ATP binding	9.19E-22	58	1662	4.511268841	P2RX7 AKT1 PIM1 FYN PRKCH FLT4 PIK3CB SPHK2 PRKCQ FGFR1 GSK3B AURKA JAK2 MAPK1 CSNK2A1 HCK FLT1 MAPK3 JAK3 PIK3CG MET MAPK8 MAPK10 MAPK14 PIK3CA FLT3 CDK2 PRKCG KDR CDK4 CDK9 KIF11 PDPK1 ERBB2 EGFR CACNA1B PRKCA KIT DYRK1A BRAF JAK1 PRKCD CDK5 SYK VCP RET PRKCB MAP2K1 PTK2 PRKCE PIK3CD AURKB LCK LRRK2 TRPV1 NTRK1 MTOR PRKDC
GO:0032559	Adenyl ribonucleotide binding	2.03E-22	60	1729	4.485986805	P2RX7 AKT1 HMGCR PIM1 FYN PRKCH FLT4 PIK3CB SPHK2 PRKCQ FGFR1 GSK3B AURKA JAK2 MAPK1 CSNK2A1 HCK FLT1 MAPK3 JAK3 PIK3CG MET MAPK8 MAPK10 MAPK14 PIK3CA FLT3 CDK2 PRKCG KDR CDK4 CDK9 KIF11 PDPK1 ERBB2 EGFR CACNA1B PRKCA KIT DYRK1A BRAF JAK1 PRKCD CDK5 SYK VCP RET PRKCB MAP2K1 PTK2 PRKCE PIK3CD AURKB LCK LRRK2 TRPV1 NTRK1 MTOR PRKDC PDE4A
GO:0030554	Adenyl nucleotide binding	2.48E-22	60	1741	4.455066735	P2RX7 AKT1 HMGCR PIM1 FYN PRKCH FLT4 PIK3CB SPHK2 PRKCQ FGFR1 GSK3B AURKA JAK2 MAPK1 CSNK2A1 HCK FLT1 MAPK3 JAK3 PIK3CG

						MET MAPK8 MAPK10 MAPK14 PIK3CA FLT3 CDK2 PRKCG KDR CDK4 CDK9 KIF11 PDPK1 ERBB2 EGFR CACNA1B PRKCA KIT DYRK1A BRAF JAK1 PRKCD CDK5 SYK VCP RET PRKCB MAP2K1 PTK2 PRKCE PIK3CD AURKB LCK LRRK2 TRPV1 NTRK1 MTOR PRKDC PDE4A
GO:0019899	Enzyme binding	1.93E-26	73	2237	4.218505414	RAC1 PDE3B PGR ESR1 JAK2 PARP4 CASP9 ERBB2 PRKCA PRKCD AR PTK2 PRKCE PRKCH HDAC4 PTPRC AURKA SIRT1 MAPK1 CCND1 CCND3 NR3C1 PPARG CDK9 AKT1 PARP1 KIT CDK5 SYK VCP STAT3 GRM5 RELA PTPN11 LRRK2 PTPN1 HDAC2 NTRK1 TNF MIF PRKDC FYN FLT4 PTGS2 PSEN1 GSK3B HDAC6 MAPK3 JAK3 MET MAPK8 MAPK14 HDAC1 CCND2 ADORA2A NR1H2 MDM2 KIF11 PDPK1 EGFR ACE JAK1 CDC25A PRKCB HDAC3 PTPN2 AURKB LCK HTT ELANE GSTP1 SPHK2 BCHE
GO:0140096	Catalytic activity acting on a protein	1.27E-30	83	2577	4.16356557	FYN PRKCQ HDAC4 MMP2 AURKA CMA1 SIRT1 JAK2 MMP9 HCK KDR MDM2 AKT1 PRKCA ACE JAK1 PRKCD SYK PRKCB PTK2 PTPN2 AURKB PTPN11 LCK LRRK2 ELANE MTOR CREBBP CAPN1 PRKCH FLT4 FGFR1 PSEN1 PTPRC GSK3B HDAC6 MAPK1 CSNK2A1 FLT1 MAPK3 PLAT JAK3 MET MAPK8 MAPK10 MAPK14 HDAC1 FLT3 CDK2 PRKCG CASP9 CDK4 CDK9 PIM1 PDPK1 ERBB2 PARP1 EGFR HDAC8 MMP3 ADAM17 KIT DYRK1A BRAF CDC25A CDK5 RET ANPEP MAP2K1 PRKCE HDAC3 HDAC2 MMP1 NTRK1 PRKDC PIK3CB PARP4 PIK3CG PTPN1 CCND1 CCND3 PIK3CA SHH
GO:0000166	Nucleotide binding	9.96E-26	74	2381	4.017668121	NOS2 MAOB NOS1 SIRT1 RAC1 NOS3 MAOA P2RX7 AKT1 HMGCR PIM1 G6PD IMPDH2 LRRK2 FYN PRKCH FLT4 PIK3CB SPHK2 PRKCQ FGFR1

						GSK3B AURKA JAK2 MAPK1 CSNK2A1 HCK FLT1 MAPK3 JAK3 PIK3CG MET MAPK8 MAPK10 MAPK14 PIK3CA FLT3 CDK2 PRKCG KDR DNMT1 CDK4 CDK9 KIF11 IDH1 PDE5A PDPK1 ERBB2 PARP1 EGFR CACNA1B PRKCA KIT DYRK1A BRAF JAK1 PRKCD CDK5 SYK VCP RET PRKCB MAP2K1 PTK2 PRKCE PIK3CD HSD11B2 AURKB LCK TRPV1 NTRK1 MTOR PRKDC PDE4A
GO:1901265	Nucleoside phosphate binding	9.96E-26	74	2382	4.015981443	NOS2 MAOB NOS1 SIRT1 RAC1 NOS3 MAOA P2RX7 AKT1 HMGCR PIM1 G6PD IMPDH2 LRRK2 FYN PRKCH FLT4 PIK3CB SPHK2 PRKCQ FGFR1 GSK3B AURKA JAK2 MAPK1 CSNK2A1 HCK FLT1 MAPK3 JAK3 PIK3CG MET MAPK8 MAPK10 MAPK14 PIK3CA FLT3 CDK2 PRKCG KDR DNMT1 CDK4 CDK9 KIF11 IDH1 PDE5A PDPK1 ERBB2 PARP1 EGFR CACNA1B PRKCA KIT DYRK1A BRAF JAK1 PRKCD CDK5 SYK VCP RET PRKCB MAP2K1 PTK2 PRKCE PIK3CD HSD11B2 AURKB LCK TRPV1 NTRK1 MTOR PRKDC PDE4A
GO:0032553	Ribonucleotide binding	9.32E-22	65	2123	3.957902552	NOS2 NOS1 RAC1 NOS3 P2RX7 AKT1 HMGCR PIM1 LRRK2 FYN PRKCH FLT4 PIK3CB SPHK2 PRKCQ FGFR1 GSK3B AURKA JAK2 MAPK1 CSNK2A1 HCK FLT1 MAPK3 JAK3 PIK3CG MET MAPK8 MAPK10 MAPK14 PIK3CA FLT3 CDK2 PRKCG KDR CDK4 CDK9 KIF11 PDE5A PDPK1 ERBB2 EGFR CACNA1B PRKCA KIT DYRK1A BRAF JAK1 PRKCD CDK5 SYK VCP RET PRKCB MAP2K1 PTK2 PRKCE PIK3CD AURKB LCK TRPV1 NTRK1 MTOR PRKDC PDE4A
GO:0043168	Anion binding	4.73E-26	78	2630	3.833898305	NOS2 MAOB NOS1 SIRT1 FABP3 RAC1 FABP1 NOS3 FABP4 MAOA P2RX7 AKT1 TYMS PPARD HMGCR PIM1 ACE RELA LRRK2 GRIN2B FYN PRKCH FLT4 PIK3CB SPHK2 PRKCQ FGFR1 GSK3B

						AURKA JAK2 MAPK1 CSNK2A1 HCK FLT1 MAPK3 JAK3 PIK3CG MET MAPK8 MAPK10 MAPK14 PIK3CA FLT3 CDK2 PRKCG KDR CDK4 CDK9 KIF11 PDE5A PDPK1 ERBB2 EGFR CACNA1B PRKCA KIT DYRK1A BRAF JAK1 PRKCD CDK5 SYK VCP RET PRKCB MAP2K1 PTK2 PRKCE PIK3CD AURKB LCK TRPV1 NTRK1 MTOR PRKDC PDE4A GSTP1 PPARG
GO:0032555	Purine ribonucleotide binding	8.49E-20	62	2106	3.805704444	RAC1 P2RX7 AKT1 HMGCR PIM1 LRRK2 FYN PRKCH FLT4 PIK3CB SPHK2 PRKCQ FGFR1 GSK3B AURKA JAK2 MAPK1 CSNK2A1 HCK FLT1 MAPK3 JAK3 PIK3CG MET MAPK8 MAPK10 MAPK14 PIK3CA FLT3 CDK2 PRKCG KDR CDK4 CDK9 KIF11 PDE5A PDPK1 ERBB2 EGFR CACNA1B PRKCA KIT DYRK1A BRAF JAK1 PRKCD CDK5 SYK VCP RET PRKCB MAP2K1 PTK2 PRKCE PIK3CD AURKB LCK TRPV1 NTRK1 MTOR PRKDC PDE4A
GO:0017076	Purine nucleotide binding	1.14E-19	62	2120	3.780572434	RAC1 P2RX7 AKT1 HMGCR PIM1 LRRK2 FYN PRKCH FLT4 PIK3CB SPHK2 PRKCQ FGFR1 GSK3B AURKA JAK2 MAPK1 CSNK2A1 HCK FLT1 MAPK3 JAK3 PIK3CG MET MAPK8 MAPK10 MAPK14 PIK3CA FLT3 CDK2 PRKCG KDR CDK4 CDK9 KIF11 PDE5A PDPK1 ERBB2 EGFR CACNA1B PRKCA KIT DYRK1A BRAF JAK1 PRKCD CDK5 SYK VCP RET PRKCB MAP2K1 PTK2 PRKCE PIK3CD AURKB LCK TRPV1 NTRK1 MTOR PRKDC PDE4A
GO:0036094	Small molecule binding	2.32E-26	80	2743	3.770213239	NOS2 MAOB NOS1 SIRT1 RAC1 NOS3 MAOA P2RX7 AKT1 TYMS PPARD HMGCR FABP3 PIM1 G6PD IMPDH2 LRRK2 GRIN2B FYN PRKCH FLT4 PIK3CB SPHK2 PRKCQ FGFR1 GSK3B AURKA JAK2 MAPK1 CSNK2A1 HCK FLT1 MAPK3 JAK3 PIK3CG

						MET MAPK8 MAPK10 MAPK14 PIK3CA FLT3 CDK2 PRKCG KDR DNMT1 CDK4 CDK9 KIF11 IDH1 PDE5A PDPK1 ERBB2 PARP1 EGFR CACNA1B PRKCA KIT DYRK1A BRAF JAK1 FABP1 PRKCD CDK5 SYK VCP RET PRKCB MAP2K1 PTK2 PRKCE PIK3CD HSD11B2 AURKB LCK TRPV1 NTRK1 MTOR PRKDC PDE4A PPARG
GO:0035639	Purine ribonucleoside triphosphate binding	1.97E-18	59	2034	3.749754179	RAC1 P2RX7 AKT1 PIM1 LRRK2 FYN PRKCH FLT4 PIK3CB SPHK2 PRKCQ FGFR1 GSK3B AURKA JAK2 MAPK1 CSNK2A1 HCK FLT1 MAPK3 JAK3 PIK3CG MET MAPK8 MAPK10 MAPK14 PIK3CA FLT3 CDK2 PRKCG KDR CDK4 CDK9 KIF11 PDPK1 ERBB2 EGFR CACNA1B PRKCA KIT DYRK1A BRAF JAK1 PRKCD CDK5 SYK VCP RET PRKCB MAP2K1 PTK2 PRKCE PIK3CD AURKB LCK TRPV1 NTRK1 MTOR PRKDC
GO:0042802	Identical protein binding	2.83E-20	66	2342	3.642996714	BCL2A1 MCL1 FLT4 AKT1 TERT RELA NTRK1 TNF NOS2 MAOB PTGS2 ACHE P2RX7 MAPK1 NAMPT NR3C1 FAAH MDM2 DRD2 DYRK1A VCP STAT3 TYMS IMPDH2 LRRK2 TRPV1 FYN HDAC4 DRD4 FGFR1 PGR ESR1 SIRT1 JAK2 MMP9 CSNK2A1 MAPK3 TRPA1 PIK3CG MET VEGFA BCHE TTR CNR1 KDR ADORA2A PPARG CASP9 IDH1 ERBB2 PARP1 EGFR SIGMAR1 KCNMA1 KIT BRAF G6PD GRM5 RASGRP1 LCK HTT MTOR APOBEC3G MIF AHR TLR9
GO:0097367	Carbohydrate derivative binding	8.16E-21	69	2505	3.560763219	NOS2 NOS1 RAC1 NOS3 P2RX7 AKT1 FGFR1 VEGFA HMGCR PIM1 LRRK2 ELANE FYN PRKCH FLT4 PIK3CB SPHK2 PRKCQ PTPRC GSK3B AURKA JAK2 MAPK1 CSNK2A1 HCK FLT1 MAPK3 JAK3 PIK3CG MET MAPK8 MAPK10 MAPK14 PIK3CA FLT3 CDK2 PRKCG KDR CDK4 CDK9 KIF11 PDE5A PDPK1 ERBB2 EGFR CACNA1B PRKCA KIT

						DYRK1A BRAF JAK1 PRKCD SHH CDK5 SYK VCP RET PRKCB MAP2K1 PTK2 PRKCE PIK3CD AURKB LCK TRPV1 NTRK1 MTOR PRKDC PDE4A
GO:0038023	Signaling receptor activity	7.84E-15	52	1908	3.523114096	FLT4 DRD4 FGFR1 PGR ESR1 CHRNA4 FLT1 MET AHR PPARD OPRM1 NR3C1 TACR1 CNR1 FLT3 KDR ADORA2A NR1H2 PPARG MTNR1B HCRTR2 ERBB2 NR1I3 EGFR DRD2 DRD3 NR3C2 GABRA2 KIT RET HTR3A CHRM1 GRM5 AR TRHR HTR1A GPBAR1 CHRM2 CCR3 DRD1 NTRK1 TLR9 GRIN2B P2RX7 STAT3 SIGMAR1 EDNRA TRPV1 SPHK2 PTPRC ITGA4 ANPEP
GO:0060089	Molecular transducer activity	7.84E-15	52	1908	3.523114096	FLT4 DRD4 FGFR1 PGR ESR1 CHRNA4 FLT1 MET AHR PPARD OPRM1 NR3C1 TACR1 CNR1 FLT3 KDR ADORA2A NR1H2 PPARG MTNR1B HCRTR2 ERBB2 NR1I3 EGFR DRD2 DRD3 NR3C2 GABRA2 KIT RET HTR3A CHRM1 GRM5 AR TRHR HTR1A GPBAR1 CHRM2 CCR3 DRD1 NTRK1 TLR9 GRIN2B P2RX7 STAT3 SIGMAR1 EDNRA TRPV1 SPHK2 PTPRC ITGA4 ANPEP
GO:0016740	Transferase activity	5.19E-19	67	2553	3.392545825	CREBBP FYN PRKCQ HDAC4 AURKA JAK2 HCK KDR MDM2 AKT1 PRKCA JAK1 PRKCD SYK PRKCB PTK2 AURKB LCK LRRK2 MTOR PRKCH FLT4 PIK3CB SPHK2 FGFR1 GSK3B GSTP1 MAPK1 CSNK2A1 FLT1 MAPK3 JAK3 NAMPT PIK3CG MET MAPK8 MAPK10 MAPK14 PIK3CA FLT3 CDK2 PRKCG CDK4 CDK9 PIM1 PDPK1 ERBB2 PARP1 EGFR KIT DYRK1A BRAF TERT CDK5 RET MAP2K1 PRKCE PIK3CD TYMS NTRK1 PRKDC SIRT1 PARP4 DNMT1 UGT2B7 CCND1 CCND3

Supplementary Table S6. The top 30 GO Kyoto Encyclopedia of Genes and Genomes (KEGG) pathways enriched with the overlapping genes between the predicted metabolite targets and HIV latency-associated host genes.

KEGG ID	Pathway	Enrichment FDR	nGenes	Pathway Genes	Fold Enrichment	Genes
hsa04370	VEGF signaling pathway	7.06E-25	19	59	41.62970411	MAPK14 AKT1 KDR NOS3 PIK3CA PIK3CB PIK3CD PRKCA PRKCB PRKCG MAPK1 MAPK3 MAP2K1 SPHK2 PTGS2 PTK2 RAC1 VEGFA CASP9
hsa05223	Non-small cell lung cancer	2.72E-26	21	72	37.70409605	CDK4 EGFR ERBB2 AKT1 JAK3 MET PDPK1 PIK3CA PIK3CB PIK3CD PRKCA PRKCB PRKCG MAPK1 MAPK3 MAP2K1 CCND1 RET BRAF STAT3 CASP9
hsa05212	Pancreatic cancer	7.74E-26	21	76	35.71966994	CDK4 EGFR ERBB2 AKT1 MTOR JAK1 PIK3CA PIK3CB PIK3CD MAPK1 MAPK3 MAPK8 MAPK10 MAP2K1 RAC1 CCND1 RELA BRAF STAT3 VEGFA CASP9
hsa05221	Acute myeloid leukemia	3.92E-22	18	67	34.72957248	AKT1 FLT3 MTOR KIT PIK3CA PIK3CB PIM1 PIK3CD PPARD MAPK1 MAPK3 MAP2K1 CCND1 BCL2A1 RELA BRAF STAT3 PER2
hsa01521	EGFR tyrosine kinase inhibitor resistance	1.75E-25	21	79	34.36322678	EGFR ERBB2 AKT1 MTOR GSK3B JAK1 JAK2 KDR MET PIK3CA PIK3CB PIK3CD PRKCA PRKCB PRKCG MAPK1 MAPK3 MAP2K1 BRAF STAT3 VEGFA
hsa05215	Prostate cancer	1.14E-29	25	97	33.31731609	CDK2 CREBBP EGFR ERBB2 AKT1 FGFR1 MTOR GSK3B GSTP1 AR MDM2 MMP3 MMP9 PDPK1 PIK3CA PIK3CB PIK3CD PLAT MAPK1 MAPK3 MAP2K1 CCND1 RELA BRAF CASP9
hsa05230	Central carbon metabolism in cancer	8.56E-22	18	70	33.24116223	EGFR ERBB2 AKT1 FGFR1 FLT3 MTOR G6PD IDH1 KIT MET NTRK1 PIK3CA PIK3CB PIK3CD MAPK1 MAPK3 MAP2K1 RET
hsa04917	Prolactin signaling pathway	2.94E-20	17	70	31.39443099	MAPK14 AKT1 ESR1 GSK3B JAK2 PIK3CA PIK3CB PIK3CD MAPK1 MAPK3 MAPK8 MAPK10 MAP2K1 CCND1 RELA STAT3 CCND2
hsa04933	AGE-RAGE signaling pathway in	3.00E-26	23	100	29.73237288	CDK4 MAPK14 AKT1 JAK2 MMP2 NOS3 PIK3CA PIK3CB PIM1 PIK3CD PRKCA PRKCB PRKCD PRKCE MAPK1 MAPK3 MAPK8 MAPK10 RAC1 CCND1 RELA STAT3 VEGFA

	diabetic complications					
hsa05235	PD-L1 expression and PD-1 checkpoint pathway in cancer	7.88E-23	20	89	29.04970482	RASGRP1 MAPK14 CSNK2A1 EGFR AKT1 MTOR JAK1 JAK2 LCK PIK3CA PIK3CB PIK3CD TLR9 PRKCQ MAPK1 MAPK3 MAP2K1 PTPN11 RELA STAT3
hsa01522	Endocrine resistance	9.24E-24	21	95	28.57573595	CDK4 MAPK14 EGFR ERBB2 AKT1 ESR1 MTOR MDM2 MMP2 MMP9 PIK3CA PIK3CB PIK3CD MAPK1 MAPK3 MAPK8 MAPK10 MAP2K1 PTK2 CCND1 BRAF
hsa04012	ErbB signaling pathway	2.37E-20	18	84	27.70096852	EGFR ERBB2 AKT1 MTOR GSK3B PIK3CA PIK3CB PIK3CD PRKCA PRKCB PRKCG MAPK1 MAPK3 MAPK8 MAPK10 MAP2K1 PTK2 BRAF
hsa05206	MicroRNAs in cancer	4.33E-31	30	161	24.08779872	CREBBP DNMT1 EGFR ERBB2 SIRT1 MTOR HDAC1 HDAC2 MCL1 MDM2 MET MMP9 PIK3CA PIK3CB PIM1 PIK3CD PRKCA PRKCB PRKCE PRKCG MAPK1 MAPK3 MAP2K1 PTGS2 CCND1 STAT3 VEGFA CCND2 HDAC4 CDC25A
hsa04066	HIF-1 signaling pathway	4.18E-21	20	109	23.71948375	CREBBP EGFR ERBB2 AKT1 FLT1 MTOR NOS2 NOS3 PIK3CA PIK3CB PIK3CD PRKCA PRKCB PRKCG MAPK1 MAPK3 MAP2K1 RELA STAT3 VEGFA
hsa04931	Insulin resistance	8.85E-20	19	108	22.74215317	AKT1 MTOR GSK3B NOS3 PDPK1 PIK3CA PIK3CB PIK3CD PRKCB PRKCD PRKCE PRKCQ MAPK8 MAPK10 PTPN1 PTPN11 RELA STAT3 NR1H2
hsa04919	Thyroid hormone signaling pathway	1.44E-21	21	121	22.43549517	CREBBP AKT1 ESR1 MTOR GSK3B HDAC1 HDAC2 MDM2 PDPK1 PIK3CA PIK3CB PIK3CD PRKCA PRKCB PRKCG MAPK1 MAPK3 MAP2K1 CCND1 CASP9 HDAC3
hsa04071	Sphingolipid signaling pathway	2.37E-20	20	119	21.72624982	MAPK14 AKT1 FYN NOS3 PDPK1 PIK3CA PIK3CB PIK3CD PRKCA PRKCB PRKCE PRKCG MAPK1 MAPK3 MAPK8 MAPK10 MAP2K1 SPHK2 RAC1 RELA
hsa05205	Proteoglycans in cancer	3.36E-28	30	202	19.19869106	MAPK14 EGFR ERBB2 AKT1 ESR1 FGFR1 MTOR KDR MDM2 MET MMP2 MMP9 PDPK1 PIK3CA PIK3CB PIK3CD PRKCA

						PRKCB PRKCG MAPK1 MAPK3 MAP2K1 PTK2 PTPN11 RAC1 CCND1 SHH BRAF STAT3 VEGFA
hsa04510	Focal adhesion	5.50E-27	29	200	18.74432203	EGFR ERBB2 AKT1 FLT1 FLT4 FYN GSK3B ITGA4 KDR MET PDPK1 PIK3CA PIK3CB PIK3CD PRKCA PRKCB PRKCG MAPK1 MAPK3 MAPK8 MAPK10 MAP2K1 PTK2 RAC1 CCND1 BRAF VEGFA CCND2 CCND3
hsa05161	Hepatitis B	1.45E-21	23	162	18.35331659	CDK2 CREBBP MAPK14 AKT1 JAK1 JAK2 JAK3 MMP9 PIK3CA PIK3CB PIK3CD PRKCA PRKCB PRKCG MAPK1 MAPK3 MAPK8 MAPK10 MAP2K1 RELA BRAF STAT3 CASP9
hsa05167	Kaposi sarcoma-associated herpesvirus infection	7.36E-25	27	194	17.99135069	CDK4 CCR3 CREBBP MAPK14 AKT1 MTOR GSK3B HCK JAK1 JAK2 PIK3CA PIK3CB PIK3CD PIK3CG MAPK1 MAPK3 MAPK8 MAPK10 MAP2K1 PTGS2 RAC1 CCND1 RELA STAT3 SYK VEGFA CASP9
hsa05207	Chemical carcinogenesis-receptor activation	2.13E-23	26	197	17.06117181	CHRNA4 EGFR AHR AKT1 ESR1 MTOR AR JAK2 PGR PIK3CA PIK3CB PIK3CD PRKCA PRKCB PRKCG MAPK1 MAPK3 MAP2K1 CCND1 RELA STAT3 UGT2B7 VEGFA CACNA1B CCND3 CDC25A
hsa04014	Ras signaling pathway	6.46E-24	28	235	15.40252434	RASGRP1 EGFR AKT1 FGFR1 FLT1 FLT3 FLT4 GRIN2B KDR KIT MET NTRK1 PIK3CA PIK3CB PIK3CD PLA2G2A PRKCA PRKCB PRKCG MAPK1 MAPK3 MAPK8 MAPK10 MAP2K1 PTPN11 RAC1 RELA VEGFA
hsa04015	Rap1 signaling pathway	1.57E-21	25	210	15.38942696	CNR1 ADORA2A MAPK14 DRD2 EGFR AKT1 FGFR1 FLT1 FLT4 GRIN2B KDR KIT MET PIK3CA PIK3CB PIK3CD PRKCA PRKCB PRKCG MAPK1 MAPK3 MAP2K1 RAC1 BRAF VEGFA
hsa05203	Viral carcinogenesis	1.12E-20	24	202	15.35895284	CDK2 CDK4 CCR3 CREBBP HDAC1 HDAC2 JAK1 JAK3 MDM2 PIK3CA PIK3CB PIK3CD HDAC8 MAPK1 MAPK3 RAC1 CCND1 RELA STAT3 SYK HDAC3 CCND2 CCND3 HDAC4
hsa04151	PI3K-Akt signaling pathway	1.45E-35	42	354	15.33725941	CDK2 CDK4 CHRM1 CHRM2 EGFR ERBB2 AKT1 FGFR1 FLT1 FLT3 FLT4 MTOR GSK3B ITGA4 JAK1 JAK2 JAK3 KDR KIT MCL1 MDM2 MET NOS3 NTRK1 PDPK1 PIK3CA PIK3CB PIK3CD PIK3CG PRKCA MAPK1 MAPK3 MAP2K1 PTK2 RAC1 CCND1 RELA SYK VEGFA CASP9 CCND2 CCND3

hsa05163	Human cytomegalovirus infection	4.95E-22	26	224	15.00469128	CDK4 CCR3 MAPK14 EGFR AKT1 MTOR GSK3B JAK1 MDM2 PIK3CA PIK3CB PIK3CD PRKCA PRKCB PRKCG MAPK1 MAPK3 MAP2K1 PTGS2 PTK2 RAC1 CCND1 RELA STAT3 VEGFA CASP9
hsa04020	Calcium signaling pathway	1.77E-22	27	240	14.54300847	CHRM1 CHRM2 ADORA2A DRD1 EDNRA EGFR ERBB2 FGFR1 FLT1 FLT4 GRM5 KDR MET NOS1 NOS2 NOS3 NTRK1 P2RX7 PRKCA PRKCB PRKCG SPHK2 RET TACR1 TRHR VEGFA CACNA1B
hsa04024	cAMP signaling pathway	8.40E-20	24	221	14.03849988	CHRM1 CHRM2 ADORA2A CREBBP DRD1 DRD2 EDNRA AKT1 GRIN2B HTR1A LIPE PDE3B PDE4A PIK3CA PIK3CB PIK3CD MAPK1 MAPK3 MAPK8 MAPK10 MAP2K1 RAC1 RELA BRAF
hsa05200	Pathways in cancer	8.19E-47	57	530	13.90275024	RASGRP1 CDK2 CDK4 CREBBP EDNRA EGFR ERBB2 AKT1 ESR1 FGFR1 FLT3 FLT4 MTOR GSK3B GSTP1 HDAC1 HDAC2 AR JAK1 JAK2 JAK3 KIT MDM2 MET MMP1 MMP2 MMP9 NOS2 NTRK1 PIK3CA PIK3CB PIM1 PIK3CD PPARD PPARG PRKCA PRKCB PRKCG MAPK1 MAPK3 MAPK8 MAPK10 MAP2K1 PTGS2 PTK2 RAC1 CCND1 RELA RET SHH BRAF STAT3 TERT VEGFA CASP9 CCND2 CCND3

Supplementary Table S7. Protein targets and their PDB IDs and resolutions, reference ligands and their PubChem CIDs, and docking coordinates of the hub genes in the BA-TAR-PATH network analysis prioritized for molecular docking calculations.

Hub Target	PDB ID	Resolution (Å)	Co-crystallized/ Reference Ligand	PubChem CID	Docking Coordinates (X, Y, Z)		Docking Scores (kcal/mol)						
					Center	Size	Ref.	MET 15	MET 28	MET 34	MET 119	MET 176	MET 181
PIK3CA	8TSD	2.70	RLY2608	166822065	14.5831, -12.3351, -32.0879	24.1114, 24.0278, 27.6748	-13.7	-10.3	-7.8	-7.0	-9.0	-7.0	-7.4
MAPK3	4QTB	1.40	SCH772984	24866313	34.5091, 52.6406, 49.3583	31.4599, 21.8355, 25.5544	-14.3	-10.3	-9.0	-7.4	-9.9	-6.9	-7.2
MAPK1	4QTE	1.50	VTX-11e	11634725	23.7687, 69.0307, 24.5839	20.2586, 29.4563, 23.1049	-12.2	-10.9	-8.5	-6.5	-9.5	-6.8	-7.6
AKT1	6CCY	2.18	EX4	133054002	-9.3003, 14.8881, -32.4642	26.5325, 22.3934, 22.9615	-10.4	-8.7	-7.2	-5.9	-8.3	-5.7	-6.1
EGFR	1M17	2.60	Erlotinib	176870	24.9697, -0.9466, 53.7101	28.6348, 17.9501, 21.2925	-6.8	-9.3	-7.9	-5.9	-8.1	-5.4	-5.8
RELA	1NFI	2.70	Withaferin A	265237	-13.6064, 85.5298, 111.2110	23.8280, 23.7698, 40.9534	-7.7	-7.9	-6.4	-5.3	-7.6	-4.1	-4.6
MTOR	4JSX	3.50	Torin2	51358113	51.2863, -0.9786, -45.7329	24.8771, 22.3873, 23.9441	-11.6	-9.2	-8.2	-5.8	-9.1	-5.9	-6.5
STAT3	6NJS	2.70	SI-109	139600322	9.43519, 55.1667, 3.1702	21.5095, 34.6193, 23.5683	-9.8	-6.5	-5.8	-5.2	-6.7	-4.4	-4.7
CCND1	2W96	2.30	Fascaplysin	73293	10.0201, 8.1626, 41.8755	54.0211, 43.6759, 41.0356	-7.9	-7.8	-6.7	-5.4	-8.2	-5.2	-6.0
ERBB2	3PP0	2.25	SYR127063	16736274	17.0147, 18.4658, 26.5974	26.7954, 26.6255, 22.0305	-11.4	-10.3	-9.7	-7.6	-10.4	-7.4	-8.1
GSK3B	8AV1	2.15	MH-124	167714242	10.1166, 1.7156, 27.7884	23.4966, 19.7632, 26.6403	-8.5	-9.4	-8.0	-6.4	-9.2	-5.9	-6.1

Supplementary Table S8. Topological parameters of nodes: *Candida albicans* secondary metabolites, hub targets, and KEGG pathways in the BA-TAR-PATH network construction, ranked by degree, closeness, and betweenness centralities.

Node	Degree Centrality	Closeness Centrality	Betweenness Centrality
**Secondary Metabolites**			
MET 28	13	0.52885	0.03887
MET 34	8	0.47414	0.03731
MET 176	6	0.44355	0.01266
MET 119	5	0.45082	0.03905
MET 181	3	0.36667	0.00143
MET 15	3	0.34810	0.00622
**Hub Targets**			
PIK3CA	32	0.67073	0.13347
MAPK3	29	0.59783	0.09319
MAPK1	29	0.59783	0.07711
AKT1	29	0.61111	0.08503
EGFR	21	0.51887	0.05879
RELA	20	0.50926	0.04855
MTOR	19	0.49107	0.02851
STAT3	18	0.49107	0.03098
CCND1	18	0.48246	0.02510
ERBB2	15	0.45833	0.02782
GSK3B	14	0.45833	0.03870
MDM2	11	0.42969	0.02255
JAK2	11	0.42969	0.02140
PTGS2	10	0.43651	0.03944
MMP9	9	0.42969	0.01467
ESR1	8	0.41045	0.00703
SIRT1	3	0.37671	0.00106
PPARG	2	0.37671	0.00047
PARP1	2	0.33537	0.00102
TNF	1	0.31250	0.00000
**KEGG Pathways**			
Pathways in cancer	17	0.57292	0.07100
Human cytomegalovirus infection	12	0.51887	0.01940
PI3K-Akt signaling pathway	12	0.51887	0.01777
Proteoglycans in cancer	12	0.50926	0.01754
MicroRNAs in cancer	12	0.51887	0.03195
Prostate cancer	12	0.51887	0.01749
Chemical carcinogenesis-receptor activation	11	0.50000	0.01296
Kaposi sarcoma-associated herpesvirus infection	11	0.50000	0.01590
Endocrine resistance	11	0.50000	0.01495
Prolactin signaling pathway	10	0.49107	0.01208
EGFR tyrosine kinase inhibitor resistance	10	0.50000	0.01099

Pancreatic cancer	10	0.48246	0.00748
Thyroid hormone signaling pathway	9	0.48246	0.00961
HIF-1 signaling pathway	9	0.47414	0.00608
PD-L1 expression and PD-1 checkpoint pathway in cancer	9	0.48246	0.00708
Hepatitis B	8	0.46610	0.00688
Focal adhesion	8	0.47414	0.00585
ErbB signaling pathway	8	0.47414	0.00570
AGE-RAGE signaling pathway in diabetic complications	8	0.46610	0.00516
Acute myeloid leukemia	8	0.45833	0.00356
Non-small cell lung cancer	8	0.46610	0.00457
Viral carcinogenesis	7	0.45833	0.00437
Central carbon metabolism in cancer	7	0.45833	0.00318
Ras signaling pathway	6	0.45082	0.00201
Insulin resistance	6	0.44355	0.00305
cAMP signaling pathway	5	0.43651	0.00093
Rap1 signaling pathway	5	0.44355	0.00112
Sphingolipid signaling pathway	5	0.43651	0.00093
VEGF signaling pathway	5	0.45082	0.00335
Calcium signaling pathway	2	0.34810	0.00010
